# Evaluation of ChatGPT’s responses to information needs and information seeking of dementia patients

**DOI:** 10.1038/s41598-024-61068-5

**Published:** 2024-05-04

**Authors:** Hamid Reza Saeidnia, Marcin Kozak, Brady D. Lund, Mohammad Hassanzadeh

**Affiliations:** 1 Department of Knowledge and Information Science, Tarbiat Modares University, Tehran, Iran; 2 Department of Media, Journalism and Social Communication, University of Information Technology and Management in Rzeszow, Rzeszow, Poland; 3 Department of Information Science, University of North Texas, Denton, USA

**Keywords:** ChatGPT, Information need, Information seeking, Dementia, Large language model, Health care, Medical research

## Abstract

Many people in the advanced stages of dementia require full-time caregivers, most of whom are family members who provide informal (non-specialized) care. It is important to provide these caregivers with high-quality information to help them understand and manage the symptoms and behaviors of dementia patients. This study aims to evaluate ChatGPT, a chatbot built using the Generative Pre-trained Transformer (GPT) large language model, in responding to information needs and information seeking of such informal caregivers. We identified the information needs of dementia patients based on the relevant literature (22 articles were selected from 2442 retrieved articles). From this analysis, we created a list of 31 items that describe these information needs, and used them to formulate 118 relevant questions. We then asked these questions to ChatGPT and investigated its responses. In the next phase, we asked 15 informal and 15 formal dementia-patient caregivers to analyze and evaluate these ChatGPT responses, using both quantitative (questionnaire) and qualitative (interview) approaches. In the interviews conducted, informal caregivers were more positive towards the use of ChatGPT to obtain non-specialized information about dementia compared to formal caregivers. However, ChatGPT struggled to provide satisfactory responses to more specialized (clinical) inquiries. In the questionnaire study, informal caregivers gave higher ratings to ChatGPT's responsiveness on the 31 items describing information needs, giving an overall mean score of 3.77 (SD 0.98) out of 5; the mean score among formal caregivers was 3.13 (SD 0.65), indicating that formal caregivers showed less trust in ChatGPT's responses compared to informal caregivers. ChatGPT’s responses to non-clinical information needs related to dementia patients were generally satisfactory at this stage. As this tool is still under heavy development, it holds promise for providing even higher-quality information in response to information needs, particularly when developed in collaboration with healthcare professionals. Thus, large language models such as ChatGPT can serve as valuable sources of information for informal caregivers, although they may not fully meet the needs of formal caregivers who seek specialized (clinical) answers. Nevertheless, even in its current state, ChatGPT was able to provide responses to some of the clinical questions related to dementia that were asked.

## Introduction

Providing high-quality information to caregivers of individuals with advanced dementia is crucial for understanding and managing the symptoms and behaviors associated with the disease^1,2^. In the advanced stage of dementia, individuals experience a profound decline in cognitive function, marked by severe memory loss, impaired reasoning, and difficulties with language and communication^3,4^. Behavioral changes become more pronounced, with increased agitation, aggression, and wandering tendencies^4,5^. Informal caregivers, often family members, play a significant role in providing full-time care to dementia patients^6^. To support these caregivers, it is essential to explore innovative approaches that can effectively address their information needs. Large language models have recently exploded onto the scene, with ChatGPT dominating recent discourse on the topic of chatbots and virtual assistants^7,8^. While some studies have begun to look at the feasibility of ChatGPT and similar models for addressing the information needs of patients, there remain substantial gaps in our knowledge of this topic, especially in regard to the needs of informal caregivers. This study aims to address this gap by evaluating the quality of responses supplied by ChatGPT when posed with questions relating to advanced dementia information needs.

## Literature review

### Dementia and information needs

Dementia describes a group of symptoms that affect memory, thinking, and social abilities such that a person's activities of daily living are disrupted^3,9^. This disease is caused by damage or loss of nerve cells and their connections in the brain^10^. Depending on the affected area of the brain, dementia can affect people differently and cause different symptoms^9,10^. At the advanced stages of dementia, patients lose self-care abilities as well as the ability to complete activities of daily living, such as going to the bathroom, bathing, and doing other tasks^11,12^. According to global statistics, nearly 50 million people worldwide suffer from dementia, which is expected to reach 152 million by 2050^1,13^. Apart from the physical, mental, and social effects, the disease results in a costly financial burden, which was $820 billion worldwide in 2015–2016 alone^14^.

Many people in the most advanced stages of dementia need one or more caregivers^2,15^. Most of these caregivers are family members (usually children). Since most of them have no previous knowledge or training in the field of dementia care, they are called *informal caregivers*^1,2^. Such caregivers are constantly seeking information relevant to patient needs, in order to provide high-quality care^1,16^. Unfortunately, many studies show that the majority of informal caregivers do not receive sufficient, relevant and reliable information; this is why the information needs of such caregivers need to be recognized and improved^2,17,18^. Meeting the informational needs of dementia patients should be effective in improving the management of their behavioral and psychological symptoms—and even of the legal and financial issues that can arise when one is misinformed or unsure where to look or who to trust for information^19,20^.

Several recent studies have investigated the information needs of patients with dementia, and most of these studies use technologies such as Internet searches, specialized web systems, or mobile or wearable devices, as approaches to meet such information needs^6,21,22^.

People with dementia and their informal caregivers are constantly seeking new and relevant educational resources. These resources help them summarize and identify their information needs and understand how to manage the disease. They require information that meets their needs, regardless of publication date^12,23^. Currently, the emergence of powerful new technologies such as ChatGPT is a revolution to meet the information needs of the general public^24^. People from around the world can ask ChatGPT their questions in most scientific or non-scientific fields, and they can get their answers immediately^7,24,25^. However, the critical concerns lies in the quality of these answers.

### ChatGPT: large language model to address information needs

ChatGPT is a chatbot launched by OpenAI in November 2022, built using the large language model Generative Pre-trained Transformer (GPT) 4.0^25^. It is an artificial intelligence-based chatbot that, according to its developer (Open AI), can answer virtually any question a user might provide^7,25^. Examples of ChatGPT’s capabilities include solving physics, mathematics, and programming problems, preparing blog contents, composing poetry, and writing stories^7^. In various studies, ChatGPT has been used as a therapeutic consultant or a tool to answer patients' therapeutic and supportive questions^8,26,27^.

Chen et al. (2023) stated that using ChatGPT to answer medical questions is becoming increasingly popular. However, there are significant concerns that the large language model ChatGPT is based upon (GPT-4) and similar models may generate and reinforce medical misinformation^28^. Yee Hui et al. (2023) analyzed the accuracy of ChatGPT responses to questions about knowledge, management, and emotional support in relation to cirrhosis and hepatocellular carcinoma. They concluded that the chatbot can serve as an adjunct tool for patients to obtain information and physicians to improve treatment, but some limitations exist^29^. Hopkins et al. (2023) examined this technology in terms of its accuracy and robustness in answering questions based on basic facts compared to answering complex clinical questions. They stated that ChatGPT can become an important virtual assistant to patients and healthcare providers, but there is an urgent need to involve regulators and healthcare specialists in order to create standards for large language models and artificial-intelligence assistants^30^.

As is evident from this review, many studies have investigated the capability of large language models, particularly the ChatGPT tool, to respond to questions addressing information needs of patients with chronic diseases. In this research, we analyze whether ChatGPT technology can help older adults with dementia address their information needs. We will examine whether ChatGPT, a tool powered by artificial intelligence, can meet information needs of such patients faster and better (provide deeper and more precise information in a clearer way) than other tools and resources. Additionally, we will assess the level of trust that patients and caregivers can place in the information provided by large language models in satisfying information needs.

## Methods

The present study is a descriptive-analytical research endeavor conducted in late 2022 and early 2023. In the beginning, we identified information needs of dementia patients and developed the corresponding questions. Subsequently, we posed these questions to ChatGPT and analyzed the responses provided by the model.

### Identifying information needs of dementia caregivers

To initiate the study, we conducted a literature review in order to identify information needs of dementia patients. This review was partially conducted following the PRISMA-ScR checklist and explanation for preferential reporting of systematic reviews or literature reviews^31^.

For the literature review, we used the following search strategy:

(information-seeking behavior OR help-seeking behavior) AND (assessment of needs OR health service needs OR information needs) AND (caregivers OR spouse caregivers OR family caregivers) AND (dementia OR alzheimer's disease)

Available literature was reviewed in the databases PubMed (MEDLINE), EMBASE, CINAHL, ISI Web of Science, Scopus, OpenGrey, and Ovid. A nested search strategy was utilized. This means that the sources mentioned in the articles that have been selected for the study were also reviewed for relevance.

To expand the search results, a manual search was performed in Google Scholar. Basic inclusion and exclusion criteria included that an article should be original (a research study) and written in English. Therefore, reports, review articles, systematic reviews, meta-analyses, book chapters, correspondence, editorials, mini-reviews, opinions, news, etc. were excluded. Each selected articled needed to address information needs of dementia patients, so studies investigating cognitive impairment in other contexts were excluded. The quality of the studies selected initially was then analyzed using the CASP tool^32,33^. CASP consists of 10 items. If a study included an item, it was given a score of 1, and 0 otherwise. A score of 8 or more, which means achieving at least 8 of the criteria, is considered of high quality; that with a 4–7 score, of medium quality; and that a score below 4, of low quality^33^ (Supplementary file 1).

We included all the studies that had all the required indicators to enter the qualitative assessment by the CASP tool in terms of having a clear statement of the research objectives, appropriate methodology, appropriate research design, recruitment strategy, and data collection method. We analyzed the relationship between the researcher and the participants, the attention to ethical issues, the accuracy of data analysis, the clarity of the findings, and the value of the research. Only studies that scored at least 4 (i.e., having at least 4 items of the CASP tool) were included for further analysis. After identifying the relevant articles, we read and used them to identify what information needs dementia patients and their caregivers would express and how they would most likely structure or word their search. These information needs were then grouped into 31 items, based on which we formulated 118 questions that would be asked to ChatGPT (Supplementary file 2).

### Querying of ChatGPT

The 118 queries formulated through the review process were posed to ChatGPT on April 22, 2023. One researcher entered all of the queries. In order to ensure that the answer to one query did not influence the model’s response to another, a new chat was created for each question. ChatGPT’s response to each query was recorded in a Microsoft Word file, which would then be used for further analysis. This entire process was completed in the course of one day.

### Investigation of ChatGPT responses

The questions posed to ChatGPT and its corresponding answers were evaluated by two distinct groups of caregivers:

- informal caregivers, that is, patients’ family members, and
- formal caregivers, that is, neurologists and expert nurses.

The participants, who were divided into groups for completion of this activity, were tasked with the following:

(i) evaluate the level of correctness of these answers,
(ii) assess whether the responses were in their opinion scientific enough or not, and
(iii) compare the quality of the responses compared to that of the sources of information that they had normally used to find the corresponding information, e.g., the internet, mobile applications, textbooks, medical pamphlets.

Following completion of the questionnaire, participants were asked to participate in a separate, in-person, semi-structured interview conducted by one of the researchers. The interview focused on the following questions related to the caregiver’s experiences with the ChatGPT responses:

- Was the information obtained from ChatGPT complete, clear, correct, and understandable?
- Compare ChatGPT to the previous methods you used. Was it more useful to gather the information needs you were looking for?
- Is ChatGPT a useful tool to collect the information needs you were looking for?
- As ChatGPT evolves, can it be used to meet the informational needs of dementia patients?

In addition, at the end of the interviews, both groups were asked to complete a questionnaire. This questionnaire asked both groups whether ChatGPT met their information needs within 31 categories of Dementia-related information needs. Both groups—formal and informal caregivers—gave their opinion on each of the 31 information needs based on using a 5-point Likert scale (5 = “I agree very much” and 1 = “I don't agree at all”). With this data, we could measure the quantitative view of both groups regarding the response of ChatGPT in addressing information needs (Supplementary file 3). Both the questionnaire and the interviews were conducted in Persian. They and their results were then translated into English for the purposes of analysis.

The number of participants included in this study was chosen based on previous studies^15,34^. It was determined that a sample of 30 people would be sufficient: 15 informal caregivers and 15 formal caregivers. Participants were selected using purposive sampling, a nonprobability sampling method, necessary in order to ensure that sufficient numbers of respondents from the target populations could be acquired^35^. Formal caregivers were expert neurologists and nurses working in several Neurological Research Centers in Tehran, Iran. Informal caregivers were recruited through a call on social networks (Instagram, Facebook and Telegram). We used the following inclusion and exclusion criteria to select participants for these two groups;

- *Patients*: We selected patients who were in an advanced stage of the disease; patients with onset and those with mildly advanced were excluded.
- *Experts*: An expert needed to be either a practicing neurologist or an expert nurse with over five years of experience in working with dementia patients.

We acknowledge that caregivers tending to dementia patients in the advanced stages likely possess greater experience, as comprehensive insights are often acquired through navigating the complexities of later disease stages. This significant factor played a pivotal role in our decision to focus on the advanced stages of dementia care.

To analyze the data obtained from the questionnaire study, we used summary statistics (mean and standard deviation) and visualization methods. Since we used purposive sampling, which a nonprobability sampling method, the sample cannot be analyzed using typical statistical methods. In particular, we could not use formal statistical tests to verify the hypothesis that there is no difference between the formal and informal caregivers. Therefore, we based our interpretation solely on descriptive statistics and visualization methods.

### Ethics approval and consent to participate

Provider participants in the response to the questionnaire provided informed consent. We ensured participants that all their information was kept confidential during the collection phase and respected their privacy. The study protocol was approved by the Ethics Committee of Tarbiat Modares University, Tehran, Iran (IR.MODARES.REC.1402.011). Also, all methods were performed in accordance with the relevant guidelines and regulations by including a Declaration of Helsinki.

## Results

Out of a total of 2442 retrieved articles, 1858 articles were duplicated. Here, “duplicated” means that an article was found in two or more databases, and among them, all but one were marked as duplicated. After removing them, a total of 584 articles remained, of which 346 were excluded based on the inclusion and exclusion criteria. Finally, by reviewing their abstracts and full texts, irrelevant articles were removed—and so, ultimately, we arrived at a collection of 20 relevant articles. In addition, two relevant articles were also found by manually searching Google Scholar and through our nested search strategy.

**Figure 1:**
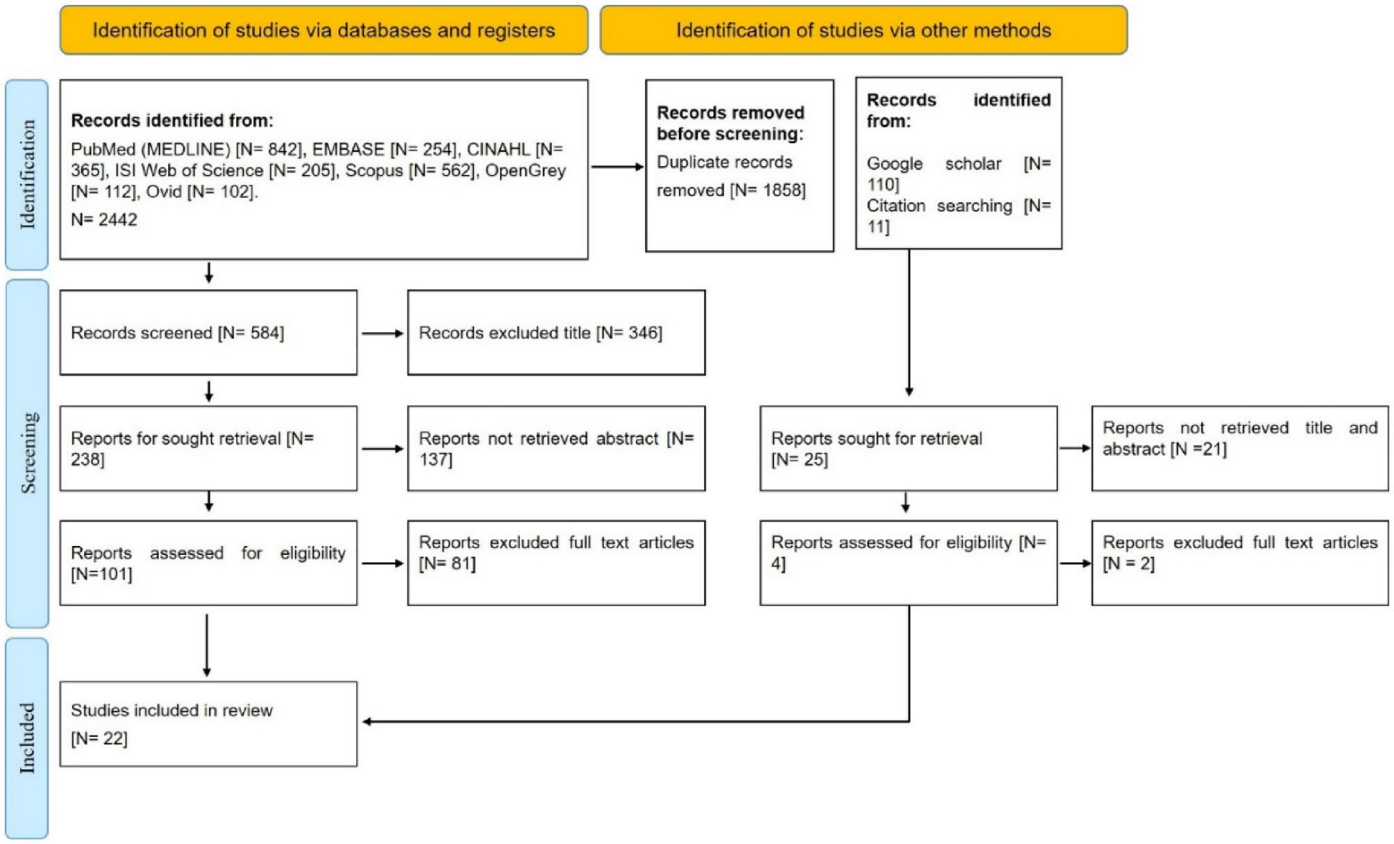
The PRISMA diagram picturing the screening process of the articles included in the study.

Thus, this method led to a final collection of 22 articles (Fig. 1) from which to extract our understanding of the information needs of dementia patients. In these articles, we found 31 important information items related to patient information needs (Table 2). These items guided the formulation of 118 questions, which were then utilized as queries for ChatGPT.

These questions were related to the performance of ChatGPT, and dealt with such issues as.

- completeness, correctness, and comprehensibility of information
- a comparison of ChatGPT to previous methods (e.g., books, healthcare pamphlets, the internet)
- the bot’s ability to meet caregivers’ information needs
- caregivers’ ability to understand the bot’s answers
- the future of ChatGPT in the context of dementia patients and their caregivers

### Interview with formal and informal caregivers

The demographic information of the participants is shown in Table 1. The number of female informal caregivers was much higher than males, as is commonly the case in caregiving situations. But among formal caregivers, the number of men was more than women. The median age range for both groups was 41–50 years old. Also, the median amount of care experience for both groups was between 3 and 5 years (Table 1).

**Table 1:** Demographic information of the study participants.

Informal caregivers				
Sex	Man (3)		Female (12)	
Age (years)	30–40 (3)	41–50 (8)	51–60 (2)	≥ 60 (2)
Experience (years)	1–3 (3)	3–5 (9)		5 < (3)
Formal caregivers				
Sex	Man (11)		Female (4)	
Age (years)	30–40 (4)	41–50 (8)	51–60 (2)	≥ 60 (1)
Specialty (years)	Neurologist (7)	Expert nurse (8)		

### Was the information obtained from ChatGPT complete, clear, correct, and understandable?

Formal and informal caregivers expressed different opinions as to the quality of information retrieved from ChatGPT. Overall, however, these comments show that caregivers consider ChatGPT a powerful tool that expresses its answers completely and clearly. However, to expect a complete and correct answer from ChatGPT, the question must be correct and clearly stated.

Informal caregiver

“*I think it responds very quickly, like a smart guide, very exciting to use.*”

Formal caregiver

“*The answers given by this tool are correct and complete at an appropriate level, I think this tool has the ability to answer more difficult questions, anyway, it provides clear, complete, and understandable answers, which is great.*”

### Is ChatGPT more useful to meet information needs than the previous methods you’ve used?

Caregivers, from both groups, emphasized that ChatGPT collects information quickly and efficiently, and using the ChatGPT platform is simple and straightforward—much more so than with any other tool. ChatGPT can access a wide range of sources, including websites, databases, and other online resources, to provide users with accurate and up-to-date information on virtually any topic. Participants indicated that the only major advantage of the tools they had used previously was that the contents were monitored or approved by a medical expert, which increased their level of confidence in the response.

Informal caregivers

“*I think this tool [ChatGPT] is much easier than searching the internet, it is much better than the mobile apps because they are more like a package that has a narrow scope. But this tool is much better, I mean it answers every question, and has no limitations.*”

*“It is easier than searching on the internet or using telemedicine or anything else, you know, it brings the same content as in Google, but faster and better, as if someone is explaining it to you. I liked it.”*

Formal caregivers

“*The tools I normally use were specifically developed to respond patient needs, and such tools must be tested and reviewed by several experts before they can be used. This increases the accuracy and reliability of the materials provided such tools […]*

“*It is easy to work with, and it is fast in answering. From these aspects, I can say that it is better than the other tools for meeting the need for information, but I have a problem with the scientific level and accuracy of the questions. I think an expert should definitely confirm the quality of the content.*”

### Is ChatGPT a useful tool to meet your information needs (in the context we discuss)?

Participants indicate that a lack of confidence in the quality of responses obtained from ChatGPT affects the usefulness of ChatGPT. Formal caregivers claim that this problem can be solved by adding expert supervision—medical experts who would monitor and, if necessary, improve the quality of the ChatGPT’s responses.

Informal caregiver

“*Yes, I am saying that you can easily and quickly solve your information needs with it, it is useful.*”.

Formal caregivers

“*As a tool that can meet information needs, not yet, this tool (ChatGPT) needs to pass a filter that identifies and validates the clinical information.*”.

“*If we don't ask specialized clinical questions and only general and basic questions about the disease, yes, it can be a useful tool.*”.

### Do you think that if ChatGPT is further developed, it could be used to meet the information needs of dementia patients?

The caregivers from both groups agreed that with the further evolution of ChatGPT focused on providing more scientific and accurate answers, we may expect the reliability of ChatGPT’s responses to improve. Nevertheless, they claim that for this evolution to succeed, it should involve an interdisciplinary approach involving not just computer scientists, but also healthcare providers and caregivers. This could help ensure that the technology matches the needs and preferences of the target population.

Informal caregiver

Definitely, this tool can better meet information needs in its future versions. You know, there is still that feeling of the unreliability of the content. Honestly, after every question I ask, I go to search the internet again.

Formal caregivers

Definitely, I think that in the future, all the information needs of patients will be solved by these artificial intelligence tools.”

This is the issue that I emphasize, I hope that this development will happen in the near future and that this tool will be effective in meeting the information needs of patients even in clinical responses.

### Results of the questionnaire study

All questionnaires were completed in full. Informal caregivers gave higher ratings to ChatGPT's level of responsiveness on 31 items of informational needs, giving an overall average of 3.77 (± 0.98 SD) out of 5 for ChatGPT's level of responsiveness. Formal caregivers were less positive about the tool, with the score of 3.13 (± 0.65 SD) out of 5. This shows that formal caregivers, who have much deeper knowledge about dementia and dealing with it in real life, place less trust in ChatGPT’s responses to information needs than informal caregivers (Table 2).

**Table 2:** Mean evaluation of formal and informal caregivers to the questionnaire (the mean ± SD).

Category of information needs for patients with Dementia	Informal caregivers	Formal caregivers
	Mean ± SD	Mean ± SD
Treatment	3.80 ± 0.86	2.80 ± 0.41
Prognosis	4.06 ± 0.89	2.93 ± 0.45
Current Medication	4.73 ± 0.37	3.00 ± 0.65
Current Research	4.53 ± 0.50	3.46 ± 0.63
Genetic Aspects	4.00 ± 0.86	2.66 ± 0.48
Hygiene	4.20 ± 0.80	3.53 ± 0.51
Controlling Emotions	3.60 ± 0.84	3.33 ± 0.72
Stress Controlling	3.73 ± 1.01	3.26 ± 0.70
Modes of Transportation	4.40 ± 0.51	3.46 ± 0.51
Home Help	4.13 ± 0.83	3.33 ± 0.48
Insurance Issues	3.13 ± 0.86	2.86 ± 0.63
Legal Issues	3.80 ± 0.85	3.26 ± 0.45
Financial Contribution and Services	4.06 ± 1.14	3.26 ± 0.70
Use Services	3.80 ± 1.37	3.13 ± 0.91
Move to the Hospital	2.93 ± 1.09	2.66 ± 0.81
Advocate for Patients with Dementia	3.93 ± 0.98	3.26 ± 0.59
How to deal with the Family and Friends of Patients	3.60 ± 1.12	3.13 ± 0.63
Negative Effects on Family and Community	3.66 ± 0.96	3.20 ± 0.56
Information appropriate to different levels	3.86 ± 1.00	3.13 ± 0.74
Chance of Recovery	4.13 ± 0.95	3.46 ± 0.63
Memory Skills Patients with Dementia	3.86 ± 1.09	3.20 ± 0.67
Food and Nutritional Information	4.40 ± 0.76	3.46 ± 0.51
Behavior and Safety issues	3.46 ± 1.05	3.20 ± 0.77
Coping with hallucinations	2.86 ± 0.75	2.66 ± 0.48
Communication difficulties and how to manage	3.33 ± 0.81	3.13 ± 0.63
Daily Activities for Patients with Dementia	4.40 ± 0.73	3.53 ± 0.51
First aid for Patients with Dementia	3.06 ± 0.79	2.86 ± 0.63
Emergency situations	2.80 ± 0.67	2.66 ± 0.48
Conflict resolution for Patients with Dementia	3.33 ± 0.72	3.06 ± 0.59
Patient Ethics	3.53 ± 0.91	3.00 ± 0.53
Helpful Experiences of Other Caregivers	3.73 ± 1.03	3.20 ± 0.67
Total Mean	3.77 ± 0.98	3.13 ± 0.65

**Figure 2:**
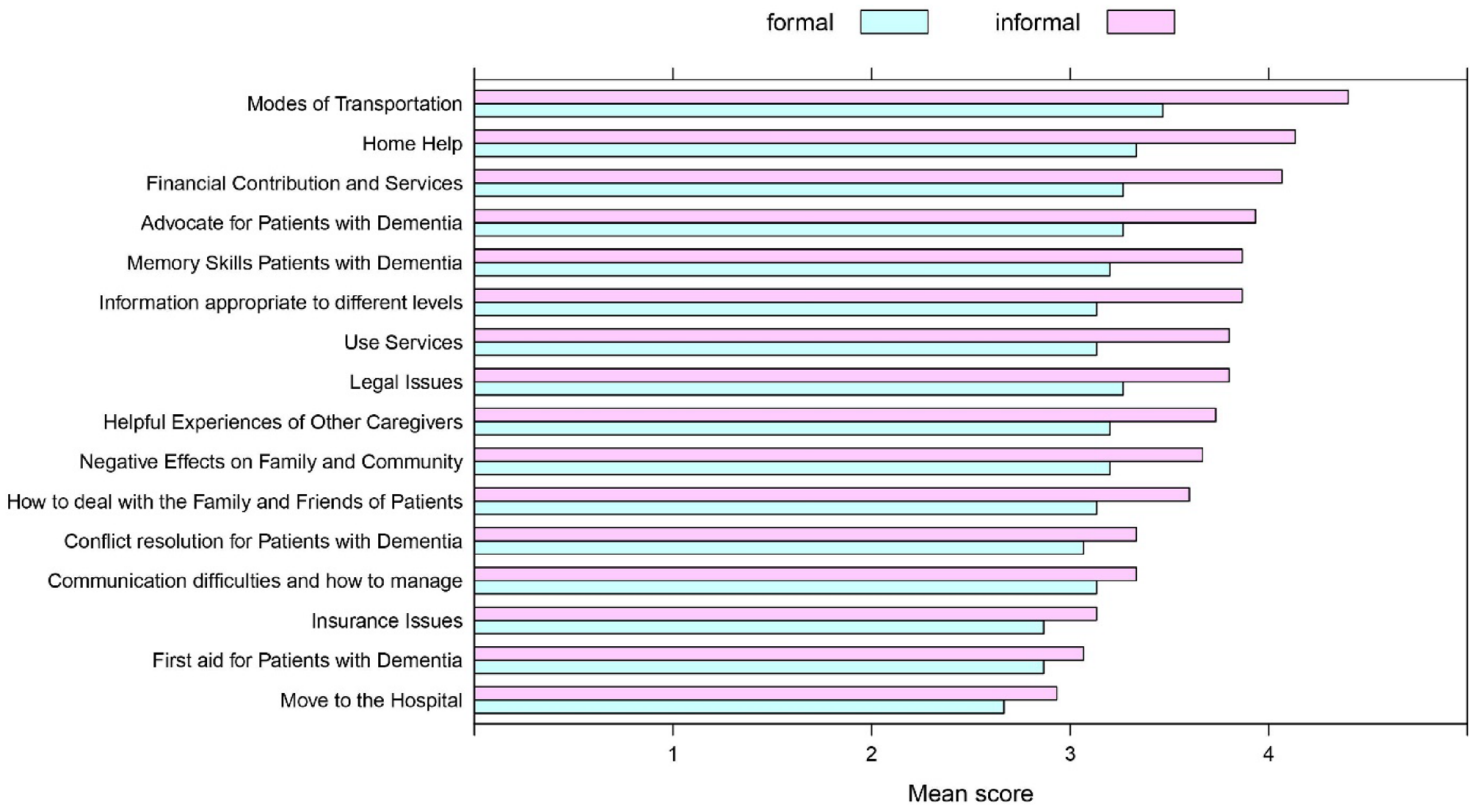
The mean evaluation of ChatGPT’s responses to non-clinical questions, grouped to formal and informal caregivers.

**Figure 3:**
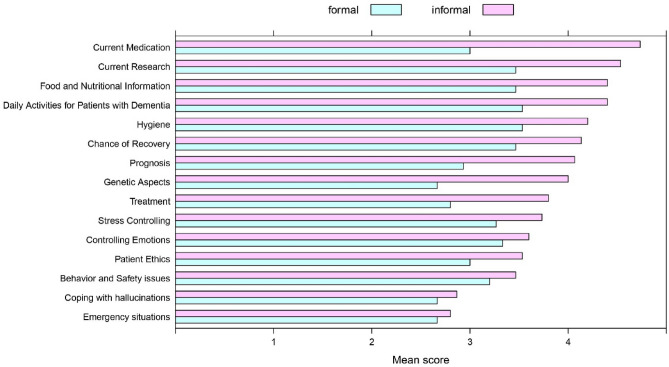
The mean evaluation of ChatGPT’s responses to clinical questions, grouped to formal and informal caregivers.

Out of 31 categories of information items, 17 were related to non-clinical information needs, and 15 to clinical information needs. Figure 2 shows the difference in evaluations by formal and informal caregivers of ChatPGT’s responses to non-clinical (simpler) questions grouped into information items. Figure 3 shows the same for clinical information items.

**Figure 4:**
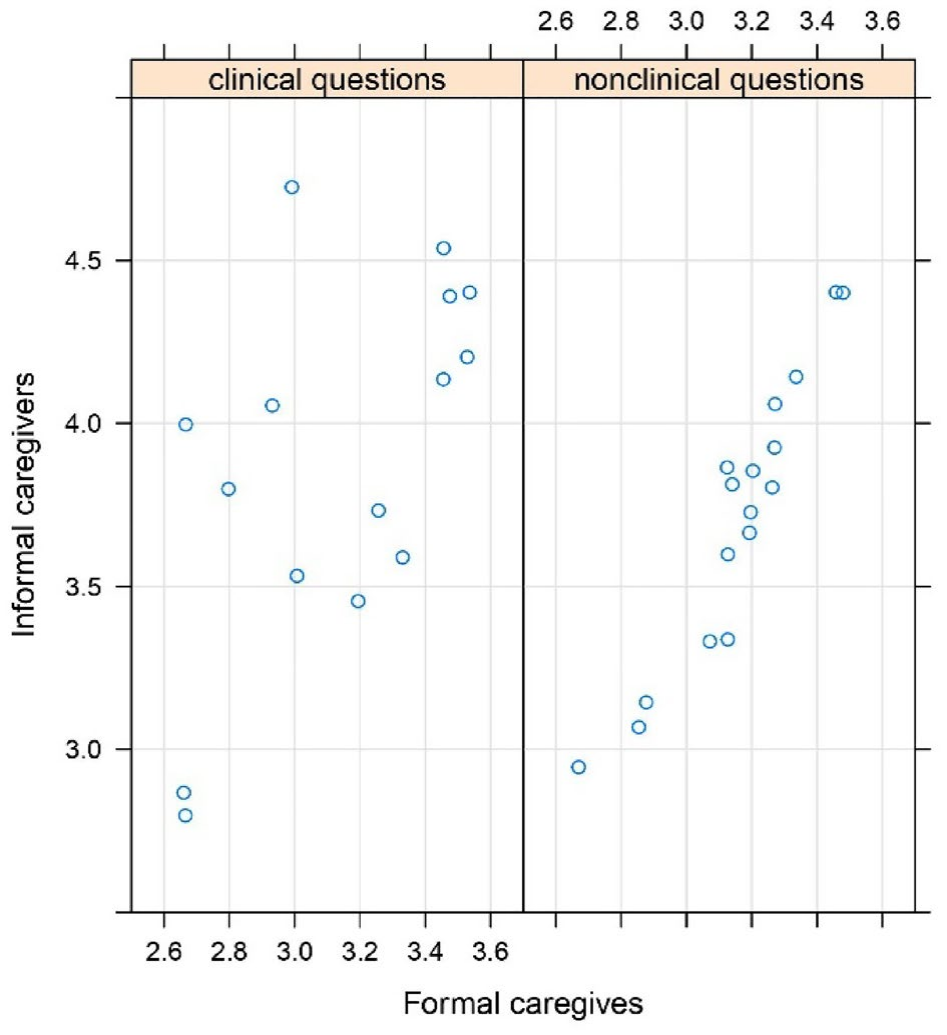
The difference in responses of formal and informal caregivers to questions grouped to clinical and non-clinical items.

Formal caregivers considered the quality of ChatGPT’s responses to be significantly lower than information caregivers did, in both clinical and non-clinical questions. We can see very strong correlation (*r* = 0.93) between both groups’ evaluations of non-clinical questions (Fig. 4), although formal caregivers’ evaluations were much lower than those of informal ones. In the case of clinical questions, the results were different. Although formal caregivers’ evaluations were significantly lower, the correlation between their and informal caregivers’ evaluations was much smaller (*r* = 0.58). This finding, as well as the findings documented in Fig. 4, suggest that informal caregivers could accept the chatbot’s response to a difficult question for which they did not have an answer. Formal caregivers were less willing to accept the responses produced by ChatGPT, possibly due to their deeper knowledge and expertise and reluctance to defer to a chatbot.

## Discussion

### Summary of findings and their significance

The 22 articles selected for inclusion in the study helped us come up with 31 key items in terms of dementia patients and caregivers’ information needs. Based on these information needs, we created 118 questions that reflect the wide range of needs expressed on this topic. These questions were posed to ChatGPT. The received responses were then analyzed by formal caregivers (neurologists and expert nurses), in a semi-structured interview. Despite the widespread recognition of ChatGPT's remarkable capabilities in answering questions across various subjects, it faced limitations when responding to clinical inquiries. In some instances, its responses were incorrect. Formal caregivers, in particular, expressed reservations about relying solely on this tool to meet their information needs. They preferred content that had been either provided or approved by a human expert, such as a medical doctor.

Informal caregivers, however, did see some usefulness in ChatGPT for addressing their information needs. The tool was generally considered to be quite helpful in meeting these individuals’ information needs. Formal caregivers gave a lower mean score (3.13 ± 0.65) to the level of ChatGPT capability than informal caregivers did (3.77 ± 0.98). The difference between the two mean scores was smaller in non-clinical issues (e.g., insurance issues or helpful experiences of other caregivers) than in clinical ones (e.g., treatment, prognosis, current medication, genetic aspects). This suggests that formal caregivers were less satisfied with the answers to more specialized questions than informal caregivers.

### Comparisons with existing literature

Several recent empirical studies have demonstrated that ChatGPT can provide accurate and up-to-date information on any topic, owing to its access to a wide range of data sources in the development of the underlying large language model^36–38^. In the case of medical topics, however, the chatbot appears to not perform as well. This may be because medicine, and especially detailed questions requiring deep medical knowledge, requires both deep medical knowledge and higher order thinking and problem solving, which artificial intelligence has yet to sufficiently master^39^. ChatGPT has knowledge, but does not yet think—at least not in the same way people do.

In their study, Aguirre et al. (2023 ) set out to evaluate the quality of ChatGPT responses provided to dementia caregivers. Recognizing the scarcity of existing evidence on this topic, the researchers conducted an exploratory investigation to fill this knowledge gap. They began by selecting 25 social media posts that reflected the information needs of dementia caregivers regarding daily care for memory loss and confusion. Subsequently, they collected responses generated by ChatGPT and assessed their quality using a 4-item rating scale. Three clinicians, each possessing over 15 years of experience working with dementia caregivers, independently evaluated the responses. Disagreements among the clinicians primarily revolved around the comprehensiveness and specificity of the responses. Following a reconciliation process, all raters concurred that the ChatGPT responses demonstrated a high level of quality^40^.

In a study, Dosso, et al. (2024) conducted a comparative analysis focusing on the quality of information related to Alzheimer’s disease and dementia provided to users of ChatGPT. Their investigation sought to evaluate how this information stacks up against content sourced from reputable non-commercial entities specializing in Alzheimer’s disease and dementia. The primary aim was to replicate the search behavior of a novice user engaging with ChatGPT for the first time and to assess the quality of the material retrieved from the Large Language Model in comparison to publicly available resources from Alzheimer’s disease-related organizations in North America. The study's findings indicated that users interacting with ChatGPT are likely to access accurate yet superficial information concerning dementia^41^.

Indeed, numerous studies evaluating ChatGPT's performance in medical domains have consistently shown its unreliability in providing clinical information, particularly regarding chronic diseases^36–38,41,42^. Consequently, at present, it appears to be more suitable for addressing basic inquiries, such as those unrelated to clinical aspects. This limitation becomes evident when considering diseases beyond common ailments like the flu or a cold, as ChatGPT's effectiveness diminishes when confronted with the need for actual data processing and critical thinking. To illustrate this, ChatGPT performs well when it has access to a comprehensive knowledge base that precisely describes diseases. In such cases, it can effectively assist in enhancing understanding of the disease. However, when confronted with complex data processing tasks or when the available knowledge is indistinguishable from background noise, ChatGPT is unlikely to be of substantial assistance. Skyler et. al.’s (2023) study evaluated the ability of ChatGPT to respond to myths and common misconceptions about cancer. They found that ChatGPT’s responses were more accurate than those they obtained from the National Cancer Institute (NCI)^43^. In other studies, ChatGPT has been considered a tool with the prospect of refining personal medicine and with the ability to improve health literacy by providing accessible and understandable health information to the general public^44–46^. These insights have been further substantiated by the findings of the present study.

In the future, there is a possibility that large language models and the chatbots they power, like ChatGPT, will be capable of functioning independently in medical contexts without human assistance. However, in order to achieve this a quality measure should be implemented that enables the chatbot to assess the reliability and accuracy of its responses. If certainty in the quality of its response is low, the bot would not provide this answer, instead saying something like, “I am sorry, my current knowledge is insufficient to respond to your question. Please ask another one.” It could also be beneficial to allow users to customize and set their own quality measure for the bot's responses. This represents an area for further exploration and development when deploying intelligent bots in sensitive domains like medicine. Another critical issue is the ability of such chatbots to generate false knowledge due to failure to truly understand the meaning of the language it is processing, also known as “stochastic parroting”^41^. Based on one of the authors (MK) experience gained during discussions with ChatGPT on programming and data science, it appears that in some situations, ChatGPT tries to create knowledge. For example, when asked how to code a particular task in Python, the bot produced functions that did *not* exist in Python—although they existed in other programming languages. Initially, the code appeared acceptable, but it ultimately proved to be ineffective and incorrect. Considering the findings from various studies, professional opinions, and discussions, it can be argued that non-professionals, such as informal caregivers of dementia patients, should refrain from using AI bots without consulting a healthcare professional, except for simple questions that do not require clinical knowledge^42,43^. In such cases, the purpose of using the bot becomes questionable. If one needs to consult an expert anyway, why rely on the bot in the first place? Directly seeking information from an expert would likely yield accurate and timely responses, without the need to assess the bot's output. Thus, one might consider abandoning the idea of using the bot altogether.

However, there is still value in exploring and developing these bots further. We are in the early stages of their creation, and the optimal configuration for different scenarios is still unknown^44^. For instance, it might be advisable to configure bots involved in medical discussions, especially those related to complex clinical topics, not to generate new knowledge—at least for now. Although this may change in the future, comprehensive and quasi-clinical experiments are necessary to determine whether enabling bots to create knowledge will result in valuable insights rather than erroneous or misleading information. It is possible that bots should assess their own knowledge based on various criteria and only provide answers when the assessment indicates high confidence. Without such experiments, using such bots in an unrestricted manner would be very risky. Addressing these challenges is more complex than it may appear, requiring the involvement of a formal governing body. The nature of this body—whether it should be national, international, or based on regional entities like the European Union or individual countries—needs careful consideration. Legislation must advance rapidly, but this is only feasible with parallel sociological developments. Our findings indicate that there is a significant difference in the perspectives of formal and informal caregivers concerning clinical and specialized aspects of dementia care, such as treatment, prognosis, current medication, current research, and genetic aspects. While formal caregivers did not find ChatGPT's answers convincing, reliable, or comprehensive in these areas, informal caregivers considered the same responses helpful and of higher quality. In the interviews, specialists emphasized the need for a healthcare consultant to verify ChatGPT's answers. Interestingly, despite this, informal caregivers regarded ChatGPT as superior to previous tools and methods in meeting their information needs, primarily due to its prompt response rate. Overall, this study illustrates that ChatGPT can be a helpful tool for addressing basic information needs related to advanced dementia patients, but it likely should not be used to learn find more specialized and clinical knowledge. ChatGPT, as an artificial intelligence large language model, can handle a wide range of questions and provide quick and accurate answers. Additionally, it is available 24 h a day, 7 days a week, making it a very convenient option for users who need information outside of normal business hours. However, it is important to note that ChatGPT is not infallible and there may be instances where the information provided is not entirely accurate or appropriate^41^. Its answers about medical issues are often imperfect, and in clinical cases, a physician’s expertise and judgment are necessary. Until ChatGPT becomes a much better “thinker”, it should not be used as the only source of information about advanced topics related to dementia and the care of dementia patients.

## Strengths and limitations of this study

In this study, for the first time, answers to patients' questions from ChatGPT are analyzed and evaluated by people (formal and informal caregivers) using both quantitative and qualitative approaches. This evaluation offers valuable insights for enhancing the performance of ChatGPT in this domain. However, it is important to acknowledge the limitations of this study. Firstly, the participant sample size was small, consisting of only 30 caregivers. Additionally, there was limited diversity among the participants, as they were all from Iran and resided in the same large city. This restricts the generalizability of the findings and prevents us from capturing the perspectives of caregivers from small towns and villages. Conducting the study with a larger and more diverse population would yield more reliable and representative results.

In future research, expanding the participant pool to include a larger and more diverse sample would provide a more comprehensive understanding of caregivers' perceptions and experiences with ChatGPT. It would also be beneficial to explore the perspectives of caregivers from different cultural backgrounds and geographical locations, as this could uncover additional insights and potential variations in their assessments of ChatGPT's performance.

## Conclusion

ChatGPT has the potential to be a useful tool to meet the information needs of advanced dementia patients. Its ability to immediately answer questions combined with its access to large volumes of information from a wide variety of sources can help overcome some of the challenges in information needs of patients with dementia. However, it should be stressed that ChatGPT is not a substitute for human interaction and care for dementia patients^45^. While this chatbot can answer some questions with fair accuracy, it cannot replace the personal interactions, emotional support, and physical assistance and care that dementia patients need.

ChatGPT demonstrates a strong ability to address non-clinical questions pertaining to dementia. However, when it comes to more advanced and clinical inquiries, its performance can often be lacking. To enhance ChatGPT’s abilities, involving healthcare professionals in the ongoing development of the underlying model powering the chatbot would be beneficial. The expertise and insights of healthcare professionals can contribute to refining ChatGPT's responses and ensuring their accuracy, reliability, and relevance in clinical contexts. By leveraging the knowledge and guidance of healthcare professionals, the model can be fine-tuned to better meet the information needs of users seeking more advanced and clinical knowledge about dementia. This collaboration between AI technology and human expertise holds great potential for improving the overall performance and usefulness of ChatGPT in providing comprehensive and reliable information in the field of dementia care.

### Supplementary Information

Supplementary Information 1.

Supplementary Information 2.

Supplementary Information 3.

## Data Availability

Please contact the corresponding author if you would like access to the datasets used and/or analyzed during this study.
