# Supplementary material for: Evaluation of ChatGPT’s responses to information needs and information seeking of dementia patients
Source: Sci Rep. 2024 May 4;14:10273. doi: 10.1038/s41598-024-61068-5 (PMC11069588; doi:10.1038/s41598-024-61068-5)
Supplement: Supplementary file 2 — Supplementary Information 2. [file 41598_2024_61068_MOESM2_ESM.docx]

**Supplementary file 2**

***31 items, based on which we formulated 118 questions that would be asked to ChatGPT***

Table 1. 31 items identified from 22 reviewed articles, along with the questions derived from them

| **NO.** | **31 items** | **Articles** | **Questions about** |
| --- | --- | --- | --- |
| 1 | Treatment | (Wackerbarth and Johnson 2002, Edelman, Kuhn et al. 2006, Galvin, Duda et al. 2010, Hirakawa, Kuzuya et al. 2011, Forbes, Finkelstein et al. 2012, Vaingankar, Subramaniam et al. 2013, Ducharme, Kergoat et al. 2014) | 1- What is the best treatment for dementia?  2- Can dementia be curable?  3- What is the first-line treatment for dementia?  4- How can dementia be treated at home? |
| 2 | Prognosis | (Thomas, Chantoin‐Merlet et al. 2002, Georges, Jansen et al. 2008, Hirakawa, Kuzuya et al. 2011, Forbes, Finkelstein et al. 2012, Jensen and Inker 2015, Shreve, Baier et al. 2016) | 1- What are the symptoms of dementia?  2- How quickly does dementia progress?  3- What is the most common cause of death in dementia patients? |
| 3 | Current Medication | (Edelman, Kuhn et al. 2006, Rosa, Lussignoli et al. 2010, Killen, Flynn et al. 2016, Scharett, Madathil et al. 2017, Iribarren, Stonbraker et al. 2019) | 1- What is the latest drug treatment for dementia?  2- What is the new Alzheimer's drug in 2023?  3- What experimental drug slows Alzheimer's disease? |
| 4 | Current Research | (Wackerbarth and Johnson 2002, Farran, Loukissa et al. 2003, Galvin, Duda et al. 2010, Killen, Flynn et al. 2016, Armstrong, Gamez et al. 2021) | 1- What is the latest data on dementia?  2- What is the current research on Alzheimer's disease in 2023?  3- What is the latest research on dementia treatment?  4- Is there any new research on dementia? |
| 5 | Genetic Aspects | (Edelman, Kuhn et al. 2006, Killen, Flynn et al. 2016) | 1- What is the genetic component of dementia?  2- What are the genetic types of dementia?  3- What are the genetic risk factors for dementia?  4- Is dementia caused by gene mutations? |
| 6 | Hygiene | (Edelman, Kuhn et al. 2006, de Jong and Boersma 2009, Rosa, Lussignoli et al. 2010, Koenig, Steiner et al. 2011, Boughtwood, Shanley et al. 2012, Killen, Flynn et al. 2016, Huis in het Veld, Verkaik et al. 2018) | 1- Why is hygiene important for dementia patients?  2- How do you keep a dementia patient clean?  3- How often should dementia patients bathe?  4- What stage of dementia is not showering?  5- How to help someone with dementia with their personal hygiene? |
| 7 | Controlling Emotions | (Rosa, Lussignoli et al. 2010, Koenig, Steiner et al. 2011) | 1- How do you help someone with dementia emotionally?  2- What are 3 typical emotional responses of a person with dementia?  3- What are the coping strategies for dementia patients?  4- Why is emotional support important for dementia patients? |
| 8 | Stress Controlling | (Rosa, Lussignoli et al. 2010, Hirakawa, Kuzuya et al. 2011, Koenig, Steiner et al. 2011) | 1- What are the methods of stress management in the elderly?  2- How to respond when a person with dementia is expressing stress and distress?  3- What activities reduce anxiety for dementia patients?  4- How do you manage stress in patients? |
| 9 | Modes of Transportation | (Farran, Loukissa et al. 2003, Forbes, Finkelstein et al. 2012) | 1- Can you travel abroad if you have dementia?  2- What facility is best for dementia patients?  3- Can people with dementia travel by plane?  4- What services do dementia patients need? |
| 10 | Home Help | (Thomas, Chantoin‐Merlet et al. 2002, Vaingankar, Subramaniam et al. 2013, Shreve, Baier et al. 2016) | 1- What dementia home care services are available?  2- How do I prepare to give dementia care at home? |
| 11 | Insurance Issues | (Wackerbarth and Johnson 2002, Iribarren, Stonbraker et al. 2019) | 1- Does dementia affect life insurance?  2- How does dementia affect travel insurance?  3- What are the actual problems for dementia patients?  4- Can I claim benefits if I have dementia? |
| 12 | Legal Issues | (Wackerbarth and Johnson 2002, Hirakawa, Kuzuya et al. 2011, Forbes, Finkelstein et al. 2012, Scharett, Madathil et al. 2017, Iribarren, Stonbraker et al. 2019) | 1- What are the 4 issues people with dementia have?  2- What is an ethical issue with dementia patients?  3-What are the practical issues affecting patients with dementia?  4- What are the rights of a dementia patient? |
| 13 | Financial Contribution and Services | (Turner and Street 1999, Ducharme, Kergoat et al. 2014, Jensen and Inker 2015, Scharett, Madathil et al. 2017) | 1- How can I help a dementia patient with money?  2- What services can help people with dementia?  3- What is the financial impact of dementia? |
| 14 | Use Services | (Rosa, Lussignoli et al. 2010, Hirakawa, Kuzuya et al. 2011, Forbes, Finkelstein et al. 2012, Ducharme, Kergoat et al. 2014) | 1- What services do dementia patients need?  2- What are dementia care services?  3- Where are most patients with dementia cared for?  4- What is the best way to help someone with dementia? |
| 15 | Move to the Hospital | (Rosa, Lussignoli et al. 2010, Hirakawa, Kuzuya et al. 2011, Ducharme, Kergoat et al. 2014) | 1- When should you take someone to the hospital for dementia?  2- Why do people with dementia go to hospital?  3- Is it better to keep dementia patient at home?  4- What are signs that dementia is getting worse? |
| 16 | Advocate for Patients with Dementia | (Rosa, Lussignoli et al. 2010, Hirakawa, Kuzuya et al. 2011, Forbes, Finkelstein et al. 2012, Ducharme, Kergoat et al. 2014) | 1- What is the role of advocacy in dementia patients?  2- How do we help dementia patients?  3- How do I advocate for Alzheimer's disease? |
| 17 | How to deal with the Family and Friends of Patients | (Edelman, Kuhn et al. 2006, Ducharme, Kergoat et al. 2014) | 1- How can family and friends help someone with dementia?  2- How do you deal with a friend with dementia?  3- How do you deal with family conflict and dementia? |
| 18 | Negative Effects on Family and Community | (Edelman, Kuhn et al. 2006, Hirakawa, Kuzuya et al. 2011, Ducharme, Kergoat et al. 2014) | 1- What is the negative impact on dementia patients?  2- How does dementia affect your social life?  3- What are the impacts of Alzheimer's disease on the family?  4- What are the possible effects of caring for a dementia client on the family of the client? |
| 19 | Information appropriate to different levels | (Rosa, Lussignoli et al. 2010, Vaingankar, Subramaniam et al. 2013) | 1- What information is on the early stages of dementia?  2- What are the different stages of dementia?  3- What is general information about dementia?  4- How do you identify the 7 stages of dementia? |
| 20 | Chance of Recovery | (Rosa, Lussignoli et al. 2010, Hirakawa, Kuzuya et al. 2011, Vaingankar, Subramaniam et al. 2013, Huis in het Veld, Verkaik et al. 2018) | 1- Can you fully recover from dementia?  2- What are the chances of surviving dementia?  3- Is dementia treatable if caught early?  4- How long does dementia take to heal? |
| 21 | Memory Skills Patients with Dementia | (Koenig, Steiner et al. 2011, Killen, Flynn et al. 2016) | 1- What is the 5-word memory test?  2- How to improve memory with dementia?  3- What is the 3-word memory test?  4- What kind of memory does dementia affect? |
| 22 | Food and Nutritional Information | (Rosa, Lussignoli et al. 2010, Koenig, Steiner et al. 2011, Vaingankar, Subramaniam et al. 2013, Huis in het Veld, Verkaik et al. 2018) | 1- What are the nutritional needs of patients with dementia?  2- How to help someone with dementia with nutrition?  3- What are the food restrictions for dementia patients?  4- How do you give food and fluids to patients with dementia? |
| 23 | Behavior and Safety issues | (Koenig, Steiner et al. 2011, Forbes, Finkelstein et al. 2012, Ducharme, Kergoat et al. 2014, Killen, Flynn et al. 2016) | 1- How do you deal with Alzheimer's patients?  2- What not to do with a dementia patient?  3- How can you maintain the safety of dementia patients?  4- How can you help a client with dementia feel safe and secure during care?  5- Why is safety needed for dementia patients? |
| 24 | Coping with hallucinations | (Koenig, Steiner et al. 2011, Killen, Flynn et al. 2016, Huis in het Veld, Verkaik et al. 2018) | 1- How do you deal with someone who hallucinates from dementia?  2- What are the coping strategies for dementia patients?  3- What stage of dementia are hallucinations?  4- How do you calm hallucinations in the elderly? |
| 25 | Communication difficulties and how to manage | (Edelman, Kuhn et al. 2006, Koenig, Steiner et al. 2011, Killen, Flynn et al. 2016, Huis in het Veld, Verkaik et al. 2018) | 1- What are 3 common communication difficulties for people with dementia?  2- What can help improve communication with a patient who has dementia?  3- What types of actions can make communicating with residents with dementia difficult?  4- Why communication is difficult with dementia? |
| 26 | Daily Activities for Patients with Dementia | (Edelman, Kuhn et al. 2006, Galvin, Duda et al. 2010, Iribarren, Stonbraker et al. 2019) | 1- What are daily activities in dementia?  2- Why are activities important for dementia patients?  3- What activities can people with dementia or Alzheimer's do? |
| 27 | First aid for Patients with Dementia | (de Jong and Boersma 2009, Hirakawa, Kuzuya et al. 2011, Killen, Flynn et al. 2016) | 1- What is the first aid for dementia patients?  2- What are the essential coping strategies for dementia?  3- What are the 4 steps when addressing a patient with dementia?  4- What is the medical management of dementia? |
| 28 | Emergency situations | (Galvin, Duda et al. 2010, Vaingankar, Subramaniam et al. 2013, Ducharme, Kergoat et al. 2014) | 1- When is dementia an emergency?  2- What should a caregiver do to help a dementia client in an emergency?  3- What safety precautions should dementia patients take in the hospital?  4- What do you do when a dementia patient is out of control? |
| 29 | Conflict resolution for Patients with Dementia | (Shreve, Baier et al. 2016, Scharett, Madathil et al. 2017) | 1- How do you handle conflict with dementia patients?  2- What strategies can you use to help a person with dementia cope?  3- How do you calm a combative dementia patient?  4- How do you redirect an angry dementia patient? |
| 30 | Patient Ethics | (Shreve, Baier et al. 2016, Scharett, Madathil et al. 2017) | 1- What are the ethics of patient dementia?  2- How cultural and ethical issues impact dementia?  3- What are the ethical issues of Alzheimer's disease?  4- What are ethical considerations when treating patients? |
| 31 | Helpful Experiences of Other Caregivers | (Koenig, Steiner et al. 2011, Killen, Flynn et al. 2016) | 1- What are the experiences of carers of people with dementia?  2- How can we support the caregivers of dementia patients?  3- How do you support carers and others to support an individual with dementia in the final stages of their life?  4- What is the role of carers and others in supporting individuals with dementia? |

**Question about Dementia Treatment:**

1. What is the best treatment for dementia?

As an AI language model, I do not provide medical advice. The treatment for dementia depends on the underlying cause of the condition and the specific needs of the individual patient. It's essential to consult a qualified healthcare professional for an accurate diagnosis and appropriate treatment plan.

That being said, some common approaches to managing dementia may include:

1. Medication: There are various medications that may be prescribed to manage symptoms of dementia, such as cholinesterase inhibitors (e.g., donepezil, rivastigmine) and N-methyl-D-aspartate (NMDA) receptor antagonists (e.g., memantine). These medications may help improve cognitive function, manage behavioral symptoms, and slow down the progression of certain types of dementia.
2. Non-pharmacological interventions: Non-drug interventions may also be beneficial in managing dementia symptoms. These may include cognitive stimulation therapy, reminiscence therapy, music therapy, art therapy, and occupational therapy, among others. These interventions aim to improve cognitive function, enhance quality of life, and manage behavioral symptoms.
3. Supportive care: Creating a supportive and safe environment for individuals with dementia is crucial. This may involve making modifications to the home environment to reduce hazards, providing a structured routine, and ensuring the person's physical, emotional, and social needs are met.
4. Lifestyle changes: Maintaining a healthy lifestyle can be beneficial for managing dementia. This may include regular physical exercise, a balanced diet, adequate sleep, and social engagement. These lifestyle changes may help promote brain health and improve overall well-being.
5. Caregiver support: Caring for someone with dementia can be challenging, and caregivers may benefit from support and resources. This may involve education about dementia, counseling, support groups, respite care, and assistance with managing daily caregiving responsibilities.

The best treatment for dementia will depend on the individual's specific condition, the severity of symptoms, and other relevant factors. It's important to work with a healthcare professional to develop a personalized treatment plan tailored to the needs of the person with dementia.

Top of Form

1. Can dementia be curable?

Dementia is a broad term used to describe a group of symptoms that affect cognitive function, such as memory, thinking, and communication. Dementia is often caused by underlying conditions that damage brain cells, and in some cases, these underlying conditions may be treatable, but dementia itself is generally considered to be irreversible and incurable.

However, it's important to note that the management and treatment of dementia can help alleviate symptoms, slow down disease progression, and improve the quality of life for individuals with dementia and their caregivers. Medications, non-pharmacological interventions, supportive care, lifestyle changes, and caregiver support can all play a significant role in managing dementia and optimizing well-being.

In some cases, the underlying cause of dementia may be treatable. For example, some forms of dementia, such as those caused by vitamin deficiencies, metabolic disorders, or infections, may be reversible with appropriate treatment. It's essential to identify and manage any underlying conditions that may be contributing to dementia symptoms.

Research into dementia is ongoing, and there may be future advancements in treatment options. However, as of now, there is no known cure for dementia. It's important to consult with a qualified healthcare professional for an accurate diagnosis, appropriate treatment plan, and ongoing management of dementia symptoms.

1. Top of Form

3.What is the first-line treatment for dementia?

The first-line treatment for dementia depends on the underlying cause and the specific symptoms of the individual. There are various types of dementia, such as Alzheimer's disease, vascular dementia, Lewy body dementia, frontotemporal dementia, and others, each with its own unique features and management approaches. The first-line treatment for dementia is typically tailored to the individual's needs and may involve a combination of pharmacological and non-pharmacological interventions.

1. Pharmacological interventions: Depending on the type and stage of dementia, medications may be prescribed to manage symptoms and slow down the disease progression. Cholinesterase inhibitors, such as donepezil, rivastigmine, and galantamine, are commonly used for Alzheimer's disease and may be considered as a first-line treatment to improve cognitive function. Memantine, an N-methyl-D-aspartate (NMDA) receptor antagonist, is another medication that may be used to manage moderate to severe Alzheimer's disease or other types of dementia. However, the use of medication and the specific choice of drug should be determined by a qualified healthcare professional based on the individual's condition and needs.
2. Non-pharmacological interventions: Non-drug interventions can also be an important part of first-line treatment for dementia. These may include cognitive stimulation therapy, physical exercise, occupational therapy, speech and language therapy, and other non-pharmacological approaches that aim to improve cognitive function, manage behavioral symptoms, and enhance the individual's quality of life.
3. Supportive care: Creating a supportive and safe environment is crucial in managing dementia. This may involve modifications to the home environment to reduce hazards, establishing a structured routine, and ensuring the person's physical, emotional, and social needs are met. Supportive care also includes caregiver support, education, and resources to help caregivers manage the challenges of caring for someone with dementia.

It's important to note that the first-line treatment for dementia should be determined by a qualified healthcare professional based on a thorough evaluation of the individual's condition, symptoms, and medical history. Treatment plans may need to be adjusted over time as the disease progresses, and ongoing monitoring and management are typically required.

1. How can dementia be treated at home?

Dementia management at home may involve a combination of strategies to provide a safe and supportive environment for individuals with dementia. Here are some general recommendations for managing dementia at home:

1. Safety modifications: Make modifications to the home environment to reduce hazards and promote safety. This may include removing tripping hazards, installing handrails and grab bars, securing cabinets with locks or childproof latches, ensuring proper lighting, and using monitoring devices such as door alarms or motion sensors to prevent wandering.
2. Daily routine: Establishing a consistent daily routine can help provide structure and familiarity for individuals with dementia. This may involve maintaining regular meal times, bedtime routines, and daily activities, which can help reduce confusion and agitation.
3. Communication strategies: Using simple and clear communication can be effective when interacting with individuals with dementia. Speak slowly, use simple words and sentences, and provide visual cues to aid understanding. Avoid arguing or correcting the person if they are confused or agitated, and instead, redirect or distract them.
4. Cognitive stimulation: Engage individuals with dementia in activities that can help stimulate their cognitive function. This may include puzzles, games, reading, listening to music, or engaging in hobbies or activities they enjoy. These activities can help promote cognitive function and enhance quality of life.
5. Medication management: Ensure that individuals with dementia are taking their prescribed medications as directed. It may be helpful to use pill organizers or reminder systems to help with medication management, and involve a healthcare professional or caregiver in monitoring and managing medications.
6. Emotional support: Providing emotional support and reassurance is crucial for individuals with dementia. Show empathy, patience, and understanding, and provide emotional comfort when they are distressed or anxious. Offer companionship, engage in conversation, and provide social interaction to help reduce feelings of isolation or loneliness.
7. Caregiver support: Caring for someone with dementia can be challenging, and caregivers may need support as well. Seek support from healthcare professionals, join caregiver support groups, and consider respite care options to provide caregivers with breaks and self-care opportunities.

It's important to work closely with a healthcare professional to develop a comprehensive care plan for managing dementia at home. Each individual with dementia is unique, and the care plan should be tailored to their specific needs and condition. Regular monitoring and adjustments to the care plan may be necessary as the disease progresses.

**Question about dementia prognosis:**

1. What are the symptoms of dementia?

Dementia is a syndrome that encompasses a range of symptoms that affect cognitive function, behavior, and daily activities. The specific symptoms of dementia can vary depending on the type of dementia and the stage of the disease. Common symptoms of dementia may include:

1. Memory loss: Memory loss is often one of the earliest and most prominent symptoms of dementia. This may involve difficulty remembering recent events, forgetting names of familiar people or objects, repeating questions, and relying on memory aids or notes for daily tasks.
2. Impaired cognitive function: Dementia can affect various cognitive functions, including difficulties with problem-solving, reasoning, judgment, and decision-making. Individuals with dementia may have trouble planning and organizing tasks, making decisions, and understanding abstract concepts.
3. Language and communication problems: Dementia can affect language and communication skills. This may involve difficulty finding the right words, understanding or following conversations, repeating the same words or phrases, and struggling with reading or writing.
4. Changes in mood and behavior: Dementia can cause changes in mood and behavior. This may include mood swings, irritability, agitation, depression, anxiety, and changes in personality or behavior. Individuals with dementia may also exhibit socially inappropriate behaviors, become withdrawn or apathetic, or show signs of paranoia or delusions.
5. Difficulty with daily activities: As dementia progresses, individuals may have difficulty performing everyday activities independently. This may involve difficulties with dressing, bathing, grooming, eating, and managing household tasks, such as cooking or managing finances.
6. Disorientation: Disorientation to time, place, and person is common in dementia. Individuals may become confused about the date, time, and location, and may have difficulty recognizing familiar people, places, and objects.
7. Wandering and getting lost: Individuals with dementia may wander aimlessly and get lost, even in familiar surroundings. This can pose safety risks and require careful monitoring and management.
8. Loss of motivation and interest: Dementia can cause a loss of motivation and interest in previously enjoyed activities or hobbies. Individuals may lose their initiative, become passive, and show decreased engagement in social or recreational activities.

It's important to note that the symptoms of dementia can vary widely depending on the type and stage of the disease, and not all individuals with dementia will experience all of these symptoms. If you or a loved one are experiencing concerning symptoms, it's important to seek evaluation and diagnosis from a qualified healthcare professional for proper assessment and management.

1. How quickly does dementia progress?

The progression of dementia can vary significantly depending on the type of dementia, the individual's overall health, and other factors. Dementia is generally a progressive condition, meaning that symptoms tend to worsen over time. However, the rate of progression can vary widely from person to person. In some cases, dementia may progress slowly over the course of several years, while in other cases, it may progress more rapidly.

In general, Alzheimer's disease, which is the most common type of dementia, tends to progress slowly over several years. It typically starts with mild cognitive impairment (MCI), which may last for several years before progressing to more severe stages of dementia. However, the rate of progression can vary, and some individuals may experience a faster or slower progression.

Other types of dementia, such as vascular dementia, Lewy body dementia, and frontotemporal dementia, may have different rates of progression. Vascular dementia, for example, may progress more rapidly if the underlying vascular conditions worsen. Lewy body dementia, which is characterized by visual hallucinations, fluctuations in cognitive function, and parkinsonism symptoms, may have a variable rate of progression.

It's important to note that dementia progression is highly individual, and it's difficult to predict with certainty how quickly it will progress in any given individual. Factors such as overall health, genetics, lifestyle, and presence of other medical conditions can also impact the rate of dementia progression. Regular monitoring and assessment by a qualified healthcare professional are important to track changes in symptoms and adjust the care plan accordingly.

1. Top of Form
2. What is the most common cause of death in dementia patients?

The most common cause of death in individuals with dementia is often related to complications that arise due to the advanced stages of the disease. These complications can vary depending on the type of dementia, the overall health of the individual, and the presence of other medical conditions. Some common causes of death in dementia patients may include:

1. Infections: Dementia can impair the immune system and increase the risk of infections, such as pneumonia, urinary tract infections, and skin infections. These infections can be severe and may lead to sepsis, a life-threatening condition, especially in advanced stages of dementia.
2. Aspiration pneumonia: Individuals with advanced dementia may have difficulty swallowing and coordinating their oral and pharyngeal muscles, leading to aspiration pneumonia. This occurs when food, saliva, or other fluids are inhaled into the lungs, leading to lung infections.
3. Malnutrition and dehydration: Dementia can affect an individual's ability to eat and drink independently, leading to malnutrition and dehydration. As the disease progresses, individuals may have difficulty recognizing food or drink, remembering to eat or drink, or coordinating the physical act of eating or drinking.
4. Falls and injuries: Dementia can impair balance, coordination, and judgment, increasing the risk of falls and injuries. Falls can result in fractures, head injuries, and other complications, especially in older individuals with weakened bones.
5. Secondary medical conditions: Dementia can increase the risk of developing other medical conditions, such as cardiovascular disease, diabetes, and respiratory problems, which can contribute to mortality in advanced stages of the disease.

It's important to note that dementia itself is a terminal illness, and individuals with advanced dementia are at increased risk of various complications that can contribute to death. Palliative care, which focuses on managing symptoms, maintaining comfort, and improving quality of life, is often an important component of care for individuals with advanced dementia. A comprehensive care plan that addresses the specific needs and complications of the individual with dementia should be developed in consultation with a qualified healthcare professional.

**Question about Current dementia medication:**

- 1. What is the latest drug treatment for dementia?

As an AI language model, I do not have access to real-time data, and my knowledge cutoff date is September 2021. Therefore, I may not have information on the very latest drug treatments for dementia beyond that date. However, I can provide you with some information on recent advancements in drug treatments for dementia up to my knowledge cutoff date.

As of September 2021, some of the latest drug treatments for dementia that have been approved or are being investigated in clinical trials include:

1. Anti-amyloid drugs: Several drugs that target beta-amyloid, a protein implicated in the formation of brain plaques characteristic of Alzheimer's disease, have been investigated in clinical trials. Some of these drugs, such as aducanumab (Aduhelm) and lecanemab (BAN2401), have shown promising results in early trials and have been granted accelerated or conditional approval by regulatory agencies in certain countries. However, the clinical benefits and long-term effectiveness of these drugs are still being debated, and further research is ongoing.
2. Tau protein-targeting drugs: Tau protein is another protein involved in the development of Alzheimer's disease, and drugs that target tau protein are being investigated in clinical trials. These drugs aim to prevent the accumulation and spread of abnormal tau protein in the brain.
3. Anti-inflammatory drugs: Some studies have explored the role of inflammation in dementia and have investigated anti-inflammatory drugs as potential treatments. For example, drugs that target the immune system or inhibit certain inflammatory pathways have been studied in clinical trials for their potential effects on reducing neuroinflammation and improving cognitive function in dementia.
4. Other novel approaches: There are ongoing research and clinical trials investigating other novel approaches for the treatment of dementia, including drugs that target mitochondrial function, brain insulin resistance, and neuroprotection, among others.

It's important to note that the landscape of dementia drug treatments is constantly evolving, and the availability and effectiveness of treatments may change over time as new research emerges. It's recommended to consult with a qualified healthcare professional or stay updated with the latest scientific literature and clinical trial results for the most up-to-date information on the latest drug treatments for dementia.

Top of Form

- 1. What is the new Alzheimer's drug in 2023?

As an AI language model, I do not have real-time information as my knowledge cutoff date is in 2021, and I do not have browsing capabilities. Therefore, I am unable to provide information on any new Alzheimer's drug that may have been developed or approved in 2023.

The landscape of Alzheimer's disease treatment is constantly evolving, and new drugs may undergo clinical trials and gain approval by regulatory agencies such as the U.S. Food and Drug Administration (FDA) or other global health authorities. It is best to refer to reliable and up-to-date sources, such as reputable medical websites, scientific journals, and official regulatory agencies, for the latest and most accurate information on new Alzheimer's drugs in 2023 or beyond. Consulting with a qualified healthcare professional can also provide the most current and appropriate medical advice regarding Alzheimer's disease treatment.

3- What experimental drug slows Alzheimer's disease?

As of my knowledge cutoff date in 2021, there are several experimental drugs that have been studied for their potential to slow down the progression of Alzheimer's disease. It's important to note that while some experimental drugs may show promise in preclinical or early-stage clinical trials, they may not have been approved for widespread use as they are still being investigated for safety and efficacy. Some examples of experimental drugs that have been studied for their potential to slow Alzheimer's disease progression include:

1. Aducanumab: Aducanumab is a monoclonal antibody that targets amyloid beta plaques, which are believed to be a hallmark of Alzheimer's disease. In June 2021, the U.S. Food and Drug Administration (FDA) granted accelerated approval to aducanumab, marketed as Aduhelm, for the treatment of Alzheimer's disease. However, it's worth noting that the approval has been controversial and there are ongoing discussions and debates regarding its effectiveness.
2. BAN2401: BAN2401 is another monoclonal antibody that targets amyloid beta plaques. It has shown promising results in early-phase clinical trials, but further research is needed to determine its long-term safety and efficacy.
3. Lecanemab (BAN2401): Lecanemab is another monoclonal antibody targeting amyloid beta plaques that has shown promise in early-stage clinical trials. It is being studied in ongoing clinical trials to assess its safety and effectiveness in slowing Alzheimer's disease progression.
4. Anti-tau therapies: Tau is another protein that forms abnormal clumps in the brains of individuals with Alzheimer's disease. Several experimental drugs that target tau, such as LMTX, are being studied for their potential to slow Alzheimer's disease progression.

It's important to note that while these experimental drugs may show promise, further research is needed to determine their safety and efficacy, and they may not be widely available for clinical use. It's always best to consult with a qualified healthcare professional for the most up-to-date and accurate information on experimental drugs for Alzheimer's disease or any other medical condition. Clinical trials are typically the primary way to investigate the safety and efficacy of experimental drugs, and participation in such trials should always be done under the supervision of qualified healthcare professionals.

Top of Form

**Question about Current research on dementia:**

1- What is the latest data on dementia?

As an AI language model, I do not have real-time browsing capabilities and my knowledge cutoff date is in 2021. Therefore, I am unable to provide the latest data on dementia. However, as of my knowledge cutoff date, here are some general facts about dementia:

1. Prevalence: Dementia is a global health issue, and its prevalence is increasing as the global population ages. According to the World Health Organization (WHO), an estimated 50 million people worldwide were living with dementia in 2020, and this number is projected to triple by 2050.
2. Types of dementia: Alzheimer's disease is the most common form of dementia, accounting for approximately 60-70% of cases. Other common types of dementia include vascular dementia, which occurs due to reduced blood flow to the brain, and dementia with Lewy bodies, which is characterized by abnormal protein deposits in the brain.
3. Risk factors: Advanced age is a significant risk factor for dementia, with the risk increasing as people get older. Other risk factors include genetics, family history, cardiovascular risk factors such as hypertension, diabetes, smoking, and a sedentary lifestyle, as well as certain modifiable lifestyle factors such as physical inactivity, poor diet, and cognitive inactivity.
4. Impact: Dementia has a significant impact on individuals, families, and society as a whole. It affects memory, thinking, behavior, and the ability to perform daily activities, and can have profound emotional, financial, and caregiving burdens on individuals with dementia and their caregivers.
5. Diagnosis and management: Accurate diagnosis of dementia involves comprehensive medical and cognitive evaluations, including history taking, physical examination, neuropsychological testing, and brain imaging. Although there is no cure for dementia, management strategies may include pharmacological interventions, non-pharmacological interventions such as cognitive stimulation, physical exercise, and caregiver support, and addressing other modifiable risk factors such as cardiovascular risk factors.

It's important to note that dementia is a complex and evolving field, and the latest data and research on dementia may change over time. For the most up-to-date and accurate information on dementia, it's recommended to refer to reputable sources such as national or international health organizations, academic journals, and healthcare professionals specializing in dementia care.

Top of Form

2-What is the current research on Alzheimer's disease in 2023?

As an AI language model, I do not have real-time browsing capabilities and my knowledge cutoff date is in 2021. Therefore, I am unable to provide information on current research on Alzheimer's disease in 2023. However, as of my knowledge cutoff date, here are some general areas of ongoing research and promising directions in the field of Alzheimer's disease:

1. Amyloid beta and tau: Amyloid beta plaques and tau protein tangles are two hallmarks of Alzheimer's disease, and research continues to focus on understanding their roles in the disease and developing interventions to target them. This includes ongoing research into monoclonal antibodies and other drugs that aim to clear or prevent the formation of amyloid beta plaques, as well as strategies to modulate tau protein aggregation.
2. Biomarkers: Researchers are exploring various biomarkers, such as blood tests, cerebrospinal fluid markers, and brain imaging techniques, for early detection and accurate diagnosis of Alzheimer's disease. Biomarkers may also be used to monitor disease progression and response to treatments in clinical trials.
3. Genetics: Genetic factors are known to play a role in Alzheimer's disease, and ongoing research aims to better understand the genetic risk factors and the mechanisms by which they contribute to disease development. This includes studying genes such as APOE, TREM2, and others, as well as investigating gene editing techniques and gene therapies as potential interventions.
4. Lifestyle and modifiable risk factors: There is growing evidence that certain lifestyle factors, such as diet, exercise, sleep, cognitive and social engagement, and cardiovascular health, may impact the risk of developing Alzheimer's disease. Research continues to explore how these modifiable risk factors may be targeted in prevention and management strategies for Alzheimer's disease.
5. Combination therapies: Some researchers are investigating combination therapies that target multiple aspects of Alzheimer's disease, such as targeting both amyloid beta and tau, or combining pharmacological and non-pharmacological interventions for a more comprehensive approach to treatment.
6. Digital and technological interventions: There is growing interest in the use of digital and technological interventions, such as mobile apps, wearable devices, and digital therapeutics, for early detection, monitoring, and management of Alzheimer's disease. These interventions may involve cognitive training, remote monitoring, and other approaches to improve care and outcomes for individuals with Alzheimer's disease.

It's important to note that research in Alzheimer's disease is constantly evolving, and new findings may emerge beyond my knowledge cutoff date. For the most up-to-date and accurate information on current research on Alzheimer's disease, it's recommended to refer to reputable sources such as scientific journals, clinical trial registries, and research institutions, and consult with qualified healthcare professionals specializing in Alzheimer's disease care.

- 1. What is the latest research on dementia treatment?

As an AI language model, my knowledge cutoff date is in 2021, and I do not have real-time browsing capabilities. Therefore, I am unable to provide information on the latest research on dementia treatment beyond that date. However, as of my knowledge cutoff date, here are some general areas of ongoing research and promising directions in dementia treatment:

1. Pharmacological interventions: Researchers continue to investigate various drugs and compounds for the treatment of dementia, including Alzheimer's disease and other types of dementia. This includes medications targeting amyloid beta and tau, as well as other approaches such as anti-inflammatory drugs, antioxidants, and neurotransmitter modulators. Clinical trials are ongoing to test the safety and efficacy of these interventions.
2. Non-pharmacological interventions: Non-pharmacological interventions are also being explored as potential treatments for dementia. These may include cognitive stimulation, physical exercise, social engagement, and other non-drug approaches aimed at improving cognitive function, managing behavioral symptoms, and enhancing overall quality of life for individuals with dementia.
3. Combination therapies: Some research studies are investigating combination therapies that involve both pharmacological and non-pharmacological interventions. These may include multi-domain interventions that target multiple aspects of dementia, such as cognitive training, physical exercise, and dietary interventions, with the goal of improving outcomes and slowing disease progression.
4. Personalized medicine: There is growing interest in personalized medicine approaches for dementia treatment, which take into account an individual's unique genetic, cognitive, and lifestyle factors. This may involve tailoring treatments based on genetic profile, cognitive function, or other individual characteristics to optimize therapeutic outcomes.
5. Digital and technological interventions: Digital and technological interventions, such as virtual reality, computerized cognitive training, and telehealth, are being explored as potential treatments for dementia. These interventions may offer innovative ways to engage individuals with dementia, provide remote monitoring and support, and enhance cognitive function.
6. Caregiver support: Caregiver support interventions are also an important area of research in dementia treatment. These may include interventions aimed at improving caregiver well-being, providing education and skills training for caregivers, and offering support and resources to help caregivers better manage the care of individuals with dementia.

It's important to note that research in dementia treatment is an active and evolving field, and new findings may emerge beyond my knowledge cutoff date. For the most up-to-date and accurate information on the latest research on dementia treatment, it's recommended to refer to reputable sources such as scientific journals, clinical trial registries, and research institutions, and consult with qualified healthcare professionals specializing in dementia care.

- 1. Is there any new research on dementia?

As an AI language model, I do not have real-time browsing capabilities, and my knowledge cutoff date is in 2021. However, dementia is an active area of research, and new findings may have emerged beyond my knowledge cutoff date. Here are some general areas of ongoing research and promising directions in the field of dementia:

1. Biomarkers: Researchers are investigating various biomarkers, such as blood tests, cerebrospinal fluid markers, and brain imaging techniques, to improve early detection and accurate diagnosis of dementia. Biomarkers may also be used to monitor disease progression and response to treatments in clinical trials.
2. Genetics: Genetic factors are known to play a role in several types of dementia, and ongoing research aims to better understand the genetic risk factors and the mechanisms by which they contribute to disease development. This includes studying genes such as APOE, TREM2, and others, as well as investigating gene editing techniques and gene therapies as potential interventions.
3. Brain imaging and neuroimaging: Advances in brain imaging and neuroimaging techniques, such as magnetic resonance imaging (MRI), positron emission tomography (PET), and functional MRI (fMRI), are being used to better understand the underlying changes in the brain associated with dementia. These techniques can provide insights into the structural, functional, and molecular changes in the brain, and may help in early diagnosis and monitoring of dementia.
4. Lifestyle and modifiable risk factors: There is growing evidence that certain lifestyle factors, such as diet, exercise, sleep, cognitive and social engagement, and cardiovascular health, may impact the risk of developing dementia. Research continues to explore how these modifiable risk factors may be targeted in prevention and management strategies for dementia.
5. Artificial intelligence and digital health technologies: Artificial intelligence (AI) and digital health technologies, such as machine learning algorithms, wearable devices, mobile apps, and remote monitoring tools, are being explored for early detection, monitoring, and management of dementia. These technologies may assist in predicting cognitive decline, tracking symptoms, and providing personalized interventions.
6. Novel therapies: Researchers are exploring novel therapeutic approaches for dementia, including immunotherapies, gene therapies, and other innovative interventions. These may involve targeting specific pathways or mechanisms implicated in dementia, such as inflammation, protein misfolding, and synaptic dysfunction, with the goal of developing effective treatments.

It's important to note that dementia research is a rapidly evolving field, and new findings may have emerged since my knowledge cutoff date. For the most up-to-date and accurate information on the latest research on dementia, it's recommended to refer to reputable sources such as scientific journals, clinical trial registries, and research institutions, and consult with qualified healthcare professionals specializing in dementia research and care.

Top of Form

**Questions about Genetic aspects of the dementia:**

- 1. What is the genetic component of dementia?

Genetic factors are known to play a role in several types of dementia, including Alzheimer's disease, frontotemporal dementia, and others. Here are some key aspects of the genetic component of dementia:

1. Familial or early-onset dementia: In some cases, dementia can be caused by genetic mutations that are inherited in an autosomal dominant manner, meaning that if one parent carries the mutation, there is a 50% chance of passing it on to each child. These mutations are relatively rare and are associated with early-onset forms of dementia that typically occur before the age of 65. Examples include mutations in genes such as APP, PSEN1, and PSEN2, which are associated with early-onset Alzheimer's disease.
2. Late-onset dementia: The majority of cases of dementia, including Alzheimer's disease, occur later in life and are believed to involve a complex interplay of genetic and environmental factors. The most well-established genetic risk factor for late-onset dementia is the apolipoprotein E (APOE) gene, with the APOE ε4 allele being associated with increased risk and the APOE ε2 allele being associated with decreased risk of dementia. However, it's important to note that having the APOE ε4 allele does not necessarily mean that an individual will develop dementia, and not having the APOE ε4 allele does not guarantee protection against dementia.
3. Other genes: In addition to the APOE gene, several other genes have been implicated in the risk for dementia. For example, mutations in the MAPT gene are associated with an increased risk of frontotemporal dementia, and mutations in the C9orf72 gene are also associated with frontotemporal dementia as well as amyotrophic lateral sclerosis (ALS), a neurodegenerative disorder. Other genes, such as TREM2, SORL1, and CLU, have been implicated in late-onset Alzheimer's disease, although their exact role in the disease is still being studied.
4. Gene-environment interactions: It's important to note that dementia is likely to involve complex interactions between genetic and environmental factors. Lifestyle factors such as diet, exercise, sleep, cognitive and social engagement, and cardiovascular health may also impact the risk of developing dementia, and these lifestyle factors may interact with an individual's genetic predisposition to influence their overall risk of dementia.
5. Genetic counseling and testing: Genetic counseling and testing may be offered to individuals with a family history of dementia or suspected genetic forms of dementia. This can help individuals and their families better understand their risk of developing dementia, make informed decisions about their healthcare, and engage in appropriate preventive measures or interventions.

It's important to note that genetic factors are just one piece of the puzzle in understanding the complex nature of dementia, and not all cases of dementia are solely due to genetic factors. Environmental, lifestyle, and other factors also play important roles in the development and progression of dementia. If you are concerned about your risk of developing dementia or have a family history of dementia, it's recommended to consult with a qualified healthcare professional or a genetic counselor for personalized evaluation, counseling, and guidance.

Top of Form

- 1. What are the genetic types of dementia?

There are several genetic types of dementia, including:

1. Familial Alzheimer's disease (FAD): This is a rare form of Alzheimer's disease that is caused by genetic mutations that are inherited in an autosomal dominant manner. Mutations in the APP, PSEN1, and PSEN2 genes are known to cause FAD. People with these mutations typically develop Alzheimer's disease at an earlier age, often before the age of 65.
2. Frontotemporal dementia (FTD): FTD is a rare form of dementia that affects the frontotemporal lobes of the brain, leading to changes in personality, behavior, and language. Some cases of FTD are caused by genetic mutations that are inherited in an autosomal dominant manner. Mutations in genes such as MAPT, GRN, and C9orf72 are associated with FTD.
3. Early-onset familial forms of other dementias: There are other rare forms of dementia that can be caused by genetic mutations inherited in an autosomal dominant manner. For example, mutations in the CHMP2B gene are associated with an early-onset form of autosomal dominant frontotemporal dementia with inclusion body myopathy, a rare subtype of FTD.
4. Down syndrome: People with Down syndrome, a genetic disorder caused by an extra copy of chromosome 21, have an increased risk of developing Alzheimer's disease later in life. This is believed to be related to the presence of the APP gene on chromosome 21, which is involved in the production of amyloid beta, a protein that accumulates in the brains of people with Alzheimer's disease.
5. Genetic risk factors for late-onset dementia: Late-onset forms of dementia, including Alzheimer's disease, are believed to involve a complex interplay of genetic and environmental factors. The most well-established genetic risk factor for late-onset dementia is the apolipoprotein E (APOE) gene, with the APOE ε4 allele being associated with increased risk and the APOE ε2 allele being associated with decreased risk of dementia. However, it's important to note that having the APOE ε4 allele does not necessarily mean that an individual will develop dementia, and not having the APOE ε4 allele does not guarantee protection against dementia.

It's important to note that genetic factors are just one piece of the puzzle in understanding the complex nature of dementia, and not all cases of dementia are solely due to genetic factors. Environmental, lifestyle, and other factors also play important roles in the development and progression of dementia. If you have concerns about your risk of developing dementia or have a family history of dementia, it's recommended to consult with a qualified healthcare professional or a genetic counselor for personalized evaluation, counseling, and guidance.

- 1. What are the genetic risk factors for dementia?

Genetic risk factors for dementia refer to specific genetic variants or mutations that are associated with an increased or decreased risk of developing dementia. Here are some of the well-established genetic risk factors for dementia:

1. Apolipoprotein E (APOE) gene: The APOE gene has three common variants or alleles - APOE ε2, APOE ε3, and APOE ε4. The APOE ε4 allele is associated with an increased risk of developing dementia, including Alzheimer's disease. People who inherit one copy of the APOE ε4 allele from one parent have about 3 to 4 times increased risk of developing dementia, while those who inherit two copies (one from each parent) have an even higher risk. On the other hand, the APOE ε2 allele is associated with a decreased risk of dementia.
2. Other genes associated with Alzheimer's disease: Besides APOE, several other genes have been implicated in Alzheimer's disease, although their contribution to overall risk is smaller compared to APOE. For example, variants in genes such as TREM2, SORL1, and PICALM have been associated with increased risk of Alzheimer's disease, while variants in ABCA7 and CR1 have been associated with both increased and decreased risk, depending on the specific variant and population studied.
3. Other genes associated with frontotemporal dementia (FTD) and other dementias: Genetic mutations in genes such as MAPT, GRN, C9orf72, and others have been associated with an increased risk of frontotemporal dementia (FTD) and other forms of dementia, particularly in familial cases where the disease tends to run in families.
4. Down syndrome: People with Down syndrome, a genetic disorder caused by an extra copy of chromosome 21, have an increased risk of developing Alzheimer's disease later in life. This is believed to be related to the presence of the APP gene on chromosome 21, which is involved in the production of amyloid beta, a protein that accumulates in the brains of people with Alzheimer's disease.

It's important to note that having genetic risk factors for dementia does not necessarily mean that an individual will develop dementia, as other factors such as lifestyle, environmental, and other genetic factors also play important roles. Additionally, not all cases of dementia are solely due to genetic factors, and the exact mechanisms by which these genetic variants contribute to dementia risk are still being studied. Genetic testing for dementia risk is available, but it's typically done in specialized settings and is often accompanied by genetic counseling to help individuals understand the implications of their results. If you have concerns about your risk of developing dementia, it's recommended to consult with a qualified healthcare professional or a genetic counselor for personalized evaluation, counseling, and guidance.

- 1. Is dementia caused by gene mutations?

1. Dementia can be caused by gene mutations in certain cases, but it is not solely caused by gene mutations. Dementia is a complex condition that can have multiple causes, including genetic, environmental, and lifestyle factors.
2. There are some rare forms of dementia, known as familial or early-onset dementia, that are caused by specific gene mutations. These gene mutations are typically inherited in an autosomal dominant manner, meaning that if an individual inherits the mutated gene from one parent, they have a 50% chance of developing dementia. Examples of gene mutations that can cause familial dementia include mutations in genes such as APP, PSEN1, PSEN2, MAPT, and GRN, among others.
3. However, the majority of dementia cases, including the most common form of dementia, Alzheimer's disease, are believed to have a complex interplay of genetic and environmental factors. Genetic risk factors, such as the APOE ε4 allele mentioned earlier, can increase the risk of developing dementia, but they are not deterministic, meaning that not everyone with these risk factors will develop dementia, and individuals without these risk factors can still develop dementia.
4. Other factors, such as age, gender, education level, lifestyle (including diet, exercise, and cognitive activity), vascular health, and environmental exposures, can also influence an individual's risk of developing dementia. It's important to note that dementia is a multifactorial condition, and the exact causes and mechanisms are still not fully understood. Ongoing research is continuously uncovering new insights into the complex interplay of genetic and environmental factors in dementia, which may help improve our understanding of the condition and potentially lead to new prevention and treatment strategies in the future.
5. Top of Form

**Questions about the negative impact of Dementia on family and Community:**

- 1. What is the negative impact on dementia patients?

Dementia can have a significant negative impact on individuals who are affected by the condition, as well as on their caregivers, families, and society as a whole. Some of the common negative impacts of dementia include:

1. Cognitive decline: Dementia is characterized by progressive cognitive decline, including memory loss, impaired thinking, problem-solving difficulties, language and communication difficulties, and changes in behavior and personality. These cognitive impairments can significantly impact an individual's ability to perform everyday activities, maintain independence, and participate in social and recreational activities.
2. Functional impairment: Dementia can cause difficulties with activities of daily living (ADLs) such as bathing, dressing, eating, toileting, and mobility. As the condition progresses, individuals with dementia may require increasing levels of assistance and care, which can significantly impact their quality of life and independence.
3. Emotional and psychological distress: Dementia can cause emotional and psychological distress for both the individuals with dementia and their caregivers. Individuals with dementia may experience depression, anxiety, agitation, confusion, and frustration, which can further exacerbate their cognitive decline and impair their overall well-being. Caregivers, on the other hand, may experience increased stress, burden, and emotional strain as they provide care for their loved ones with dementia, which can impact their mental health and quality of life.
4. Social isolation and decreased quality of life: Dementia can lead to social isolation, as individuals may face difficulties with social interactions, communication, and engagement in meaningful activities. This can result in decreased social connections, reduced participation in social activities, and overall decreased quality of life for individuals with dementia.
5. Increased healthcare and economic burden: Dementia imposes a significant healthcare and economic burden on individuals, families, and society as a whole. The costs associated with diagnosis, care, and management of dementia can be substantial, including medical care, medications, home modifications, and long-term care services. The economic impact of dementia includes lost productivity and income for individuals with dementia and their caregivers, as well as increased healthcare costs for society.
6. Caregiver burden: Caregivers of individuals with dementia often face significant challenges, including increased stress, fatigue, emotional strain, and disruption of their own lives and routines. Caring for a loved one with dementia can be physically, emotionally, and mentally demanding, and caregivers may face financial, social, and emotional challenges in providing care, which can have a negative impact on their own well-being.

It's important to recognize the negative impact of dementia and its associated challenges, and to provide appropriate support and care to individuals with dementia and their caregivers to minimize these impacts and improve their overall well-being. Access to healthcare services, education, support groups, respite care, and other resources can be beneficial in managing the negative impacts of dementia and enhancing the quality of life for those affected by the condition.

Top of Form

- 1. How does dementia affect your social life?

Dementia can have a significant impact on an individual's social life. As the condition progresses, it can cause difficulties with social interactions, communication, and engagement in social activities. Some ways in which dementia may affect an individual's social life include:

1. Social withdrawal: Individuals with dementia may withdraw from social interactions due to difficulties with memory, language, and communication. They may struggle to follow conversations, remember people's names or faces, or express themselves coherently, leading to decreased participation in social activities.
2. Communication challenges: Dementia can impair an individual's ability to communicate effectively, leading to misunderstandings, confusion, and frustration in social situations. This can result in reduced social engagement, as individuals may find it challenging to express themselves or understand others.
3. Disorientation and confusion: Dementia can cause disorientation and confusion, which can lead to difficulties in social situations. Individuals may have trouble recognizing familiar places, people, or events, which can lead to increased social anxiety, withdrawal, or even avoidance of social activities.
4. Changes in behavior and personality: Dementia can also cause changes in behavior and personality, including agitation, aggression, irritability, and mood swings. These behavioral changes can disrupt social interactions and relationships, and individuals with dementia may struggle to maintain social connections and engage in social activities as they once did.
5. Reduced participation in social activities: As dementia progresses, individuals may have difficulty participating in social activities that they once enjoyed, such as hobbies, clubs, or social gatherings. This can lead to social isolation and a decreased quality of life, as individuals may feel disconnected from their social networks.
6. Impact on caregivers and family members: Dementia can also affect the social lives of caregivers and family members who provide care for individuals with dementia. Caregivers may face increased responsibilities and time commitments, which can limit their ability to engage in social activities and maintain their own social connections. Caregivers may also face emotional strain and increased stress, which can impact their social well-being.

It's important to recognize and address the social challenges associated with dementia. Strategies such as providing social support, maintaining social connections, adapting communication techniques, providing opportunities for engagement in meaningful activities, and offering respite care for caregivers can all help individuals with dementia maintain their social well-being to the extent possible. Professional support from healthcare providers, counselors, and support groups can also be valuable in navigating the social impacts of dementia and improving the overall quality of life for individuals with dementia and their caregivers.

- 1. What are the impacts of Alzheimer's disease on the family?

Alzheimer's disease, a type of dementia, can have significant impacts on the family members of individuals with the condition. Some common impacts of Alzheimer's disease on the family include:

1. Emotional distress: Alzheimer's disease can cause significant emotional distress for family members, including feelings of grief, loss, frustration, helplessness, and sadness. Witnessing the decline of a loved one's cognitive abilities and personality changes can be emotionally challenging and can affect the mental health and well-being of family members.
2. Increased caregiving responsibilities: Individuals with Alzheimer's disease often require ongoing care and support as the condition progresses. This can place significant caregiving responsibilities on family members, including spouses, children, or other close relatives. Caregiving can be physically, emotionally, and financially demanding, and can impact the caregiver's own health, employment, and quality of life.
3. Financial burden: Alzheimer's disease can also impose a financial burden on families. The cost of medical care, medications, long-term care services, and other related expenses can add up over time, and families may face financial challenges in managing the costs associated with the disease.
4. Disruption of daily routines and family dynamics: Alzheimer's disease can disrupt the daily routines and family dynamics of the affected individual and their family. Changes in communication, behavior, and personality of the person with Alzheimer's can impact family interactions, relationships, and dynamics, leading to increased stress and challenges in managing daily life.
5. Social isolation and strain on relationships: Caring for a family member with Alzheimer's disease can lead to social isolation, as caregivers may have limited time and energy to engage in social activities or maintain relationships outside of their caregiving responsibilities. The strain of caregiving can also affect relationships among family members, leading to conflicts, tension, and changes in family dynamics.
6. Psychological impact: The emotional and psychological impact of Alzheimer's disease on family members can be significant. Family members may experience feelings of guilt, anger, frustration, and grief as they witness the decline of their loved one's cognitive abilities and changes in their personality. Coping with the uncertainty and challenges of Alzheimer's disease can also affect the mental health and well-being of family members.
7. Caregiver burnout: The chronic stress and responsibilities of caregiving for a family member with Alzheimer's disease can increase the risk of caregiver burnout. Caregivers may experience physical and emotional exhaustion, sleep disturbances, increased health problems, and a decreased quality of life as a result of the caregiving role.

It's important for family members of individuals with Alzheimer's disease to seek support and care for their own well-being. This may include seeking help from healthcare professionals, engaging in self-care practices, accessing support groups or counseling services, and reaching out for assistance with caregiving responsibilities. It's also important to plan for the future, including legal and financial planning, and to communicate and make decisions together as a family to provide the best possible care for the individual with Alzheimer's while also addressing the needs and well-being of family members.

- 1. What are the possible effects of caring for a dementia client on the family of the client?

Caring for a family member with dementia can have various effects on the family of the client. Some possible effects include:

1. Emotional and psychological impact: Caregiving for a person with dementia can be emotionally and psychologically challenging. Witnessing the decline of a loved one's cognitive abilities and personality changes can lead to feelings of grief, loss, frustration, helplessness, and sadness. Caregivers may also experience anxiety, depression, and stress as they navigate the uncertainties and challenges of dementia caregiving.
2. Physical impact: Caring for a person with dementia may involve physical demands, such as assisting with activities of daily living, managing mobility issues, and providing physical care. This can result in physical strain and health issues for the caregiver, such as fatigue, back pain, sleep disturbances, and other physical health challenges.
3. Financial impact: Dementia caregiving may also have financial implications for the family. Costs associated with medical care, medications, long-term care services, home modifications, and other caregiving-related expenses can add up over time, leading to financial strain and challenges for the family.
4. Changes in daily routines and lifestyle: Caring for a person with dementia often requires significant time and energy, which can disrupt the daily routines and lifestyle of the caregiver and their family. Caregivers may have to juggle caregiving responsibilities with work, household tasks, and other responsibilities, resulting in changes to their usual routines and lifestyle.
5. Social isolation and strain on relationships: Caring for a person with dementia can lead to social isolation for the caregiver and their family. The demands of caregiving may limit the caregiver's ability to engage in social activities or maintain relationships outside of their caregiving role, resulting in social isolation. Caregiving stress can also strain relationships among family members and friends, leading to conflicts, tension, and changes in family dynamics.
6. Impact on mental and physical health: The chronic stress of caregiving for a person with dementia can take a toll on the caregiver's mental and physical health. Caregivers may experience increased risk of mental health issues, such as anxiety, depression, and caregiver burnout. Physical health may also be affected due to the physical demands of caregiving and neglect of the caregiver's own health needs.
7. Role strain and identity changes: Caregiving for a person with dementia may require the caregiver to take on new roles and responsibilities, which can lead to role strain and changes in identity. Caregivers may need to prioritize their caregiving role over other aspects of their life, such as work or personal interests, resulting in shifts in their roles and identity.

It's important for caregivers to seek support and prioritize self-care to manage the potential impacts of caring for a person with dementia. This may include seeking help from healthcare professionals, accessing respite care services, engaging in self-care practices, joining support groups, and communicating with other family members to share the caregiving responsibilities. Taking care of their own physical, emotional, and financial well-being can help caregivers better manage the challenges of dementia caregiving and maintain their own health and well-being.

**Questions about information specific to different stages of dementia:**

- 1. What information is on the early stages of dementia?

The early stages of dementia can vary depending on the type of dementia and the individual, but common signs and symptoms may include:

1. Memory loss: This is often one of the earliest signs of dementia. It may involve forgetting recently learned information, misplacing items, or having difficulty remembering important dates or events.
2. Difficulty with language and communication: This may involve difficulty finding the right words, repeating oneself, or struggling to follow or join in on conversations.
3. Confusion and disorientation: This may involve getting lost in familiar places, having difficulty recognizing familiar faces or places, or becoming confused about time, date, or season.
4. Impaired judgment and decision-making: This may involve poor judgment in decision-making, making mistakes in managing finances or personal hygiene, or showing poor judgment in social situations.
5. Difficulty with problem-solving and planning: This may involve difficulty with tasks that require planning, organizing, or problem-solving skills, such as managing finances or planning a meal.
6. Changes in mood or personality: This may involve changes in mood, such as becoming anxious, irritable, or withdrawn. Personality changes, such as increased apathy, loss of motivation, or changes in social behavior, may also occur.
7. Decreased ability to perform familiar tasks: This may involve difficulty performing familiar tasks, such as cooking, driving, or managing personal hygiene, that were previously easy and routine.
8. Decreased ability to concentrate or pay attention: This may involve difficulty staying focused, following instructions, or completing tasks that require concentration and attention.

It's important to note that the early signs of dementia may be subtle and can be easily overlooked or attributed to other factors. It's essential to consult a healthcare professional for a proper evaluation if you or a loved one are experiencing any concerning changes in cognitive function. Early detection and diagnosis of dementia can help in better managing the condition and accessing appropriate support and interventions.

Top of Form

- 1. What are the different stages of dementia?

Dementia is a progressive condition that typically advances through different stages as the cognitive function of an individual deteriorates. While the specific stages and their names may vary slightly depending on the source or the type of dementia, generally, dementia is classified into three main stages: early stage, middle stage, and late stage. Here's an overview:

1. Early stage: Also known as mild or early-stage dementia, this stage typically involves mild cognitive impairment with subtle changes in memory, language, and cognitive function. Individuals may experience difficulty with tasks that require higher-level cognitive skills, such as planning, organizing, and problem-solving. Memory loss may be noticeable, but it is often mild and may not significantly impact daily activities. Social and emotional functioning may be relatively preserved at this stage, and individuals can usually maintain independence with assistance and support.
2. Middle stage: Also known as moderate or mid-stage dementia, this stage typically involves more noticeable cognitive decline. Memory loss becomes more pronounced, and individuals may have difficulty with familiar tasks such as managing finances, preparing meals, or remembering personal details. Language and communication difficulties may worsen, and behavioral and personality changes may become more apparent. Individuals may require increased assistance with daily activities and may start to experience challenges with mobility, coordination, and self-care.
3. Late stage: Also known as severe or late-stage dementia, this stage is characterized by significant cognitive decline and functional impairment. Memory loss is severe, and individuals may have difficulty recognizing loved ones or familiar surroundings. Communication may be severely impaired, and individuals may have limited or no ability to speak or understand language. Physical functioning may be significantly impaired, with individuals requiring assistance with basic activities of daily living, such as eating, bathing, and toileting. Behavioral symptoms may be more challenging to manage, and individuals may require round-the-clock care in a specialized setting.

It's important to note that the progression of dementia can vary widely from person to person, and individuals may experience symptoms differently. Additionally, some types of dementia may have unique features or patterns of progression. It's essential to consult a healthcare professional for an accurate diagnosis, proper evaluation of the stage of dementia, and appropriate care planning.

- 1. What is general information about dementia?

Dementia is a broad term that refers to a progressive decline in cognitive function and behavior that affects a person's daily life. It is not a specific disease, but rather a syndrome caused by various underlying conditions that damage brain cells and impair cognitive function. Dementia is typically associated with memory loss, but it can also involve changes in language, problem-solving, attention, behavior, and other cognitive functions.

Here are some general key points about dementia:

1. Types of dementia: There are various types of dementia, including Alzheimer's disease, vascular dementia, Lewy body dementia, frontotemporal dementia, and others. Each type has its unique features, causes, and progression.
2. Symptoms: Dementia symptoms may include memory loss, language difficulties, impaired judgment, confusion, disorientation, changes in mood or behavior, decreased ability to perform daily tasks, and difficulties with problem-solving and planning.
3. Progressive nature: Dementia is a progressive condition, which means that symptoms tend to worsen over time. The rate and pattern of progression can vary depending on the type of dementia and the individual.
4. Risk factors: Age is the most significant risk factor for dementia, with the risk increasing with age. Other risk factors include a family history of dementia, genetics, certain medical conditions (e.g., hypertension, diabetes, high cholesterol), head injuries, lifestyle factors (e.g., lack of physical activity, unhealthy diet, smoking), and certain genetic mutations.
5. Diagnosis: Dementia is typically diagnosed through a comprehensive evaluation, including medical history, physical examination, cognitive assessments, and imaging tests. Accurate diagnosis is crucial for proper management and care planning.
6. Treatment: There is currently no cure for dementia. However, some types of dementia may be managed with medications or other interventions aimed at managing symptoms and improving quality of life. Non-pharmacological approaches, such as cognitive stimulation, physical exercise, and social engagement, may also be beneficial.
7. Caregiving: Dementia can have a significant impact on caregivers, who may provide physical, emotional, and financial support. Caregivers may face challenges related to managing behavioral symptoms, coordinating care, providing assistance with daily activities, and coping with the emotional toll of dementia caregiving.
8. Support and resources: There are various resources available for individuals with dementia and their caregivers, including support groups, caregiver training programs, respite care, and community services. It's important to seek help and support when caring for someone with dementia.

It's important to note that dementia is a complex and individualized condition, and the experience of dementia may vary widely from person to person. Consulting a healthcare professional for accurate diagnosis, care planning, and accessing appropriate support and resources is essential for individuals with dementia and their caregivers.

Top of Form

- 1. How do you identify the 7 stages of dementia?

Dementia is generally classified into stages based on the progression of symptoms and functional abilities. However, it's important to note that the staging of dementia can vary depending on the system or scale being used, and different healthcare professionals may use different staging criteria. One commonly used system for staging dementia is the Global Deterioration Scale (GDS), also known as the Reisberg Scale, which divides dementia into seven stages. Here is a general overview of the stages:

1. Stage 1: No cognitive impairment: There are no noticeable symptoms of dementia, and the individual's cognitive function is normal.
2. Stage 2: Very mild cognitive decline: The individual may experience mild forgetfulness, such as misplacing keys or forgetting familiar names, but these symptoms are often considered a normal part of aging and may not be immediately recognized as dementia.
3. Stage 3: Mild cognitive decline: Memory problems become more noticeable, and the individual may have difficulty with tasks that require concentration and organization. They may start to have difficulty with familiar activities, such as managing finances or planning.
4. Stage 4: Moderate cognitive decline: Symptoms become more evident and may include difficulty with complex tasks, language problems, decreased ability to recall recent events, and changes in behavior and mood. The individual may require more assistance with daily activities and may have difficulty recognizing familiar faces.
5. Stage 5: Moderately severe cognitive decline: The individual may experience significant memory loss, confusion about time and place, and difficulty with basic self-care tasks. They may need assistance with activities of daily living, such as dressing, bathing, and eating.
6. Stage 6: Severe cognitive decline: The individual may have little or no memory of recent events or even their own name. They may have difficulty with speech, mobility, and may require extensive assistance with all aspects of daily living. Behavioral symptoms may also be present, such as agitation, aggression, and mood swings.
7. Stage 7: Very severe cognitive decline: This is the most advanced stage of dementia, where the individual may lose the ability to communicate, walk, or eat independently. They may be bedridden and require round-the-clock care.

It's important to note that the progression of dementia can be highly individualized, and not all individuals with dementia may follow this exact staging pattern. Some individuals may progress through the stages more rapidly or slowly than others, and the symptoms and functional abilities may vary widely. Additionally, different healthcare professionals or organizations may use different staging systems or criteria. A comprehensive evaluation by a healthcare professional experienced in dementia diagnosis and management is essential for accurate staging and appropriate care planning.

**Questions about the Chance of recovery for patients affected with dementia:**

- 1. Can you fully recover from dementia?

1. As of current medical knowledge, there is no known cure for most types of dementia, including Alzheimer's disease, which is the most common type of dementia. Dementia is a progressive neurodegenerative condition that causes a decline in cognitive function and affects memory, thinking, behavior, and daily functioning. Once a person develops dementia, the condition is generally irreversible and cannot be fully cured.
2. However, it's important to note that dementia care focuses on managing the symptoms, slowing down the progression of the disease, and improving the individual's quality of life. There are interventions and treatments that may help to manage the symptoms of dementia and improve the person's well-being. These can include medications, cognitive and behavioral interventions, physical exercise, occupational therapy, speech therapy, and supportive care from caregivers and healthcare professionals.
3. In some cases, there may be reversible causes of cognitive impairment that can be addressed, such as medication side effects, vitamin deficiencies, thyroid problems, or other underlying medical conditions. In such cases, identifying and treating the underlying cause may lead to partial or full recovery of cognitive function.
4. It's important to work closely with healthcare professionals specializing in dementia care to develop an appropriate care plan that addresses the specific needs of the individual with dementia. Early diagnosis, appropriate management of symptoms, and supportive care can help optimize the person's function and quality of life, even though full recovery from dementia is currently not possible.
5. Top of Form

2- What are the chances of surviving dementia?

Dementia itself is not a fatal condition, but it is a progressive and chronic illness that affects cognitive function and daily living abilities. The life expectancy of individuals with dementia depends on various factors such as the type of dementia, the stage of the disease, overall health, and the presence of other medical conditions.

Alzheimer's disease, which is the most common type of dementia, is a progressive condition that gradually worsens over time. The life expectancy of individuals with Alzheimer's disease can vary widely, with some individuals living for many years after diagnosis, while others may have a shorter life expectancy. On average, individuals with Alzheimer's disease may live for 8 to 10 years after the initial diagnosis, but this can vary greatly from person to person.

Other types of dementia, such as vascular dementia, Lewy body dementia, frontotemporal dementia, and others, also have variable life expectancies depending on the underlying cause, disease progression, and individual health factors.

It's important to note that dementia care focuses on managing symptoms, slowing down the progression of the disease, and improving quality of life rather than aiming for a cure. Supportive care, appropriate interventions, and regular monitoring by healthcare professionals can help optimize the individual's function and well-being, but the disease itself is generally not survivable as it is a chronic and progressive condition.

3- Is dementia treatable if caught early?

While there is no known cure for most types of dementia, including Alzheimer's disease, early detection and intervention can help optimize the management of symptoms and improve the individual's quality of life. Dementia care focuses on managing symptoms, slowing down the progression of the disease, and improving overall well-being.

If dementia is caught early, appropriate interventions can be initiated to help manage the symptoms and address any reversible causes of cognitive impairment, if present. For example, if the cause of cognitive impairment is related to medication side effects, vitamin deficiencies, thyroid problems, or other underlying medical conditions, these can be addressed and managed, which may lead to partial or complete recovery of cognitive function.

Early detection also allows for the implementation of non-pharmacological interventions, such as cognitive stimulation, physical exercise, occupational therapy, and supportive care from caregivers and healthcare professionals, which may help to slow down the progression of the disease and improve the individual's function and well-being.

Additionally, early diagnosis of dementia allows the person and their family to access appropriate support services, plan for the future, and make informed decisions about care, legal, and financial matters.

It's important to note that early detection and intervention can significantly impact the management of dementia and improve the individual's quality of life, but it does not currently result in a complete cure for most types of dementia. Dementia is a chronic and progressive condition, and while early intervention can help manage symptoms and optimize care, it does not fully reverse the underlying disease process. It's important to work closely with healthcare professionals specializing in dementia care to develop an appropriate care plan based on the individual's needs and stage of the disease.

- 1. How long does dementia take to heal?

1. Dementia is a chronic and progressive condition that currently does not have a known cure. As such, dementia does not heal or go away on its own. The progression of dementia varies depending on the type of dementia, the individual's overall health, and other factors, but it is generally a long-term condition that worsens over time.
2. The duration of dementia can vary greatly from person to person. Some individuals may experience a slow progression of the disease over the course of several years, while others may experience a more rapid decline in cognitive function and daily living abilities. The average life expectancy after diagnosis of dementia, such as Alzheimer's disease, is typically several years, but this can vary depending on the individual and the type and stage of dementia.
3. It's important to note that dementia care focuses on managing symptoms, slowing down the progression of the disease, and improving the individual's quality of life through supportive care, appropriate interventions, and regular monitoring by healthcare professionals. While there is currently no known cure for most types of dementia, early detection and intervention, along with appropriate care and support, can help optimize the individual's function and well-being throughout the course of the disease. It's important to work closely with healthcare professionals specializing in dementia care to develop an appropriate care plan based on the individual's needs and stage of the disease.
4. Top of Form

**Questions about Memory Skills patients affected with dementia:**

- 1. What is the 5-word memory test?

1. The "5-word memory test" is a brief cognitive screening test that is commonly used in clinical practice to assess memory function. It involves presenting the person with a list of five unrelated words, typically read out loud or written, and then asking them to recall as many of the words as possible after a short delay, usually around 5 minutes. The person's ability to recall the words is then scored to assess their memory performance.
2. The 5-word memory test is a simple and quick cognitive screening tool that can provide a rough indication of a person's memory function, but it is not a comprehensive diagnostic tool for dementia or other cognitive disorders. It is typically used as part of a larger battery of cognitive tests and assessments to gather a more comprehensive evaluation of a person's cognitive function, including memory, attention, language, and other cognitive domains. A thorough evaluation by a qualified healthcare professional specializing in cognitive assessments is needed for accurate diagnosis and management of cognitive disorders.

2- How to improve memory with dementia?

Improving memory in individuals with dementia can be challenging due to the progressive nature of the condition. However, there are some strategies that may help support and optimize memory function for individuals with dementia. Here are some tips:

1. Create a structured routine: Establishing a consistent daily routine and structure can help individuals with dementia remember important activities and tasks. This can include regular times for meals, medication administration, and other daily activities.
2. Simplify the environment: Reducing distractions and keeping the environment simple and organized can help individuals with dementia focus and concentrate better, which may improve their memory function. Minimize noise, clutter, and unnecessary visual stimuli.
3. Use memory aids: Memory aids, such as calendars, task lists, and reminders, can help individuals with dementia remember important information, appointments, and tasks. Use simple and easy-to-understand memory aids that are tailored to the individual's cognitive abilities.
4. Provide visual cues: Visual cues can help individuals with dementia remember important information or tasks. For example, using labels on drawers or cabinets, using pictures or symbols to indicate different areas or activities, and placing visual reminders in prominent places can be helpful.
5. Engage in regular physical activity: Regular physical activity has been shown to have positive effects on cognitive function, including memory. Encourage individuals with dementia to engage in appropriate physical activities, such as walking, stretching, or other exercises, as tolerated and approved by their healthcare provider.
6. Provide cognitive stimulation: Keeping the brain engaged through cognitive stimulation activities, such as puzzles, games, reading, or engaging in hobbies, can help maintain cognitive function, including memory, in individuals with dementia.
7. Optimize nutrition and sleep: Eating a healthy and balanced diet and getting adequate sleep can support overall brain health, including memory function. Encourage individuals with dementia to eat nutritious meals and snacks, and establish a consistent sleep routine that meets their individual needs.
8. Provide emotional support: Emotional well-being can impact memory function, so providing emotional support, reducing stress, and managing any underlying mood or behavioral symptoms can potentially improve memory function in individuals with dementia.

It's important to work closely with healthcare professionals specializing in dementia care to develop an individualized care plan tailored to the person's specific needs and stage of dementia. Strategies for improving memory in dementia should be based on the individual's cognitive abilities, interests, and preferences, and should be regularly reviewed and adapted as needed.

3- What is the 3-word memory test?

The "3-word memory test" is a simple cognitive screening test that involves presenting the person with a list of three unrelated words and then asking them to recall the words after a short delay. It is often used as a quick and easy assessment of immediate memory function in clinical practice, particularly in evaluating cognitive impairment or dementia.

The person is typically given the three words to remember, either spoken or written, and then asked to recall the words after a short delay, usually around 5 minutes. The ability to recall the three words is then assessed, and the results can provide a rough indication of the person's immediate memory function.

The 3-word memory test is not a comprehensive diagnostic tool for dementia or other cognitive disorders, but rather a simple screening test that can be used as part of a larger battery of cognitive tests and assessments to gather a more comprehensive evaluation of a person's cognitive function. A qualified healthcare professional specializing in cognitive assessments should interpret the results and use them in conjunction with other assessments for accurate diagnosis and management of cognitive disorders.

Top of Form

4- What kind of memory does dementia affect?

Dementia can affect different types of memory, including:

1. Short-term or Immediate Memory: Dementia can impair short-term memory, making it difficult for individuals to remember new information or events for a brief period of time, such as recent conversations, appointments, or tasks.
2. Long-term Memory: Long-term memory is the ability to remember information or events from the past, such as childhood memories or past experiences. Dementia can affect long-term memory, leading to difficulties in recalling past events, people, and places.
3. Episodic Memory: Episodic memory involves the ability to remember specific events or experiences, including the time, place, emotions, and context associated with the event. Dementia can affect episodic memory, making it difficult for individuals to recall detailed information about past events and experiences.
4. Semantic Memory: Semantic memory is the ability to remember general knowledge and concepts, such as facts, meanings of words, or historical events. Dementia can also impact semantic memory, leading to difficulties in retaining and recalling general knowledge.
5. Procedural Memory: Procedural memory involves the ability to remember how to perform motor skills or routines, such as driving, cooking, or using tools. In the early stages of dementia, procedural memory may remain relatively preserved, but it can be affected as the disease progresses.

It's important to note that the specific type and severity of memory impairment in dementia can vary depending on the type of dementia, stage of the disease, and individual factors. A comprehensive evaluation by a qualified healthcare professional specializing in cognitive disorders is necessary for accurate diagnosis and management of dementia-related memory impairments.

**Questions about hygiene patients affected with dementia:**

- 1. Why is hygiene important for dementia patients?

Hygiene is important for dementia patients for several reasons:

1. Physical health: Good hygiene practices, such as regular bathing, grooming, and oral care, help to maintain physical health in dementia patients. Regular hygiene routines can prevent skin infections, dental issues, and other health problems that may arise due to neglect or poor personal hygiene.
2. Emotional well-being: Maintaining good hygiene can contribute to a dementia patient's emotional well-being. Feeling clean, fresh, and well-groomed can promote self-esteem, dignity, and a sense of pride in one's appearance, which can help to boost a person's mood and overall well-being.
3. Social interaction: Good hygiene practices can also facilitate social interaction for dementia patients. Poor personal hygiene can result in body odor, bad breath, and other hygiene-related issues that may lead to social isolation, embarrassment, or stigmatization. Conversely, good hygiene can enable dementia patients to feel more confident and comfortable in social settings, improving their ability to interact with others and maintain social relationships.
4. Safety: Proper hygiene can also help ensure the safety of dementia patients. Regular grooming, such as trimming nails and managing hair, can prevent injuries or accidents related to long nails, tangled hair, or other personal hygiene issues.
5. Preventing infections: Dementia patients may be more susceptible to infections due to weakened immune systems and other factors. Maintaining good hygiene, such as washing hands frequently and avoiding cross-contamination, can help prevent the spread of infections and promote better overall health.

It's important to note that dementia patients may require assistance with their hygiene routines as the disease progresses and their ability to perform self-care tasks may decline. Caregivers and healthcare professionals play a crucial role in providing appropriate support and assistance to maintain good hygiene practices in dementia patients, promoting their physical and emotional well-being.

- 1. How do you keep a dementia patient clean?

Keeping a dementia patient clean may require some additional care and assistance due to the cognitive and physical challenges associated with the condition. Here are some general tips:

1. Establish a routine: Establish a consistent daily routine for personal hygiene activities, such as bathing, grooming, oral care, and toileting. Stick to a regular schedule and try to maintain familiarity and routine to help the person with dementia feel more comfortable and cooperative.
2. Simplify the environment: Keep the bathroom and personal hygiene areas simple and clutter-free. Reduce distractions and create a calm and familiar environment to minimize confusion and agitation.
3. Use simple instructions: Use simple, clear, and concise instructions when assisting with personal hygiene tasks. Avoid complex language or long explanations, as this may be overwhelming for a person with dementia.
4. Provide visual cues: Use visual cues, such as pictures or labels, to help the person with dementia understand and follow the steps of the personal hygiene routine. For example, you can label drawers or shelves with pictures or words to indicate where personal care items are stored.
5. Use gentle and patient approach: Approach personal hygiene tasks with patience, empathy, and compassion. Be gentle and avoid rushing or forcing the person with dementia, as this may cause distress or resistance. Use a calm and reassuring tone of voice and provide positive reinforcement.
6. Involve the person as much as possible: Encourage the person with dementia to participate in their own personal care to the extent possible. This may include brushing their own teeth, combing their hair, or washing their hands with assistance.
7. Adapt to individual needs: Adjust the personal hygiene routine to the individual needs and preferences of the person with dementia. Consider their comfort level, mobility, and personal preferences when providing care. For example, if they prefer a sponge bath instead of a shower, respect their preference.
8. Use appropriate products: Use gentle and hypoallergenic products for bathing, grooming, and oral care to avoid skin irritation or discomfort. Consider using products with familiar scents or textures that the person with dementia may associate with personal hygiene.
9. Seek professional help if needed: If you are finding it challenging to keep a dementia patient clean or if the person is resistant or uncooperative, consider seeking help from healthcare professionals, such as a nurse or an occupational therapist, for guidance and assistance.

It's important to approach personal hygiene care for dementia patients with patience, understanding, and flexibility. Each person with dementia is unique, and their needs and preferences may change over time. Consulting with healthcare professionals and developing a personalized care plan can help ensure that the person with dementia maintains good hygiene while also promoting their comfort and well-being.

- 1. How often should dementia patients bathe?

The frequency of bathing for dementia patients may vary depending on their individual needs, preferences, and physical condition. It's important to consider factors such as the person's skin health, mobility, comfort level, and personal preferences when determining the appropriate frequency of bathing. Here are some general guidelines:

1. Follow the person's routine: If the person with dementia has been accustomed to a certain bathing routine prior to their diagnosis, try to maintain a similar schedule to maintain familiarity and routine.
2. Maintain good skin health: Regular bathing is important for maintaining good skin health and hygiene. However, excessive bathing or use of harsh soaps may strip the skin of natural oils and cause dryness or irritation. It's important to strike a balance and avoid over-bathing, especially in older adults who may have more fragile skin.
3. Monitor for skin issues: Keep a close eye on the person's skin for any signs of redness, rashes, or skin breakdown, which may indicate the need for more frequent bathing or changes in hygiene routine.
4. Consider individual needs: Some dementia patients may resist or become agitated during bathing, and in such cases, the frequency of bathing may need to be adjusted to minimize distress. On the other hand, some dementia patients may enjoy the sensory experience of bathing and benefit from more frequent baths.
5. Be flexible and adaptive: The bathing needs of a person with dementia may change over time due to the progression of the condition or other factors, such as changes in physical health or mobility. It's important to be flexible and adaptive in adjusting the bathing routine as needed to meet the person's changing needs.
6. Seek professional guidance: If you are unsure about the appropriate frequency of bathing for a dementia patient or if the person has specific skin or health conditions that require special care, it's recommended to consult with healthcare professionals, such as a nurse or dermatologist, for guidance and recommendations.

In general, it's important to balance the need for maintaining good hygiene with the individual preferences and comfort level of the person with dementia. Regular communication with the person's healthcare team, along with observation and assessment of the person's physical condition and skin health, can help determine the appropriate frequency of bathing for a dementia patient.

Top of Form

- 1. What stage of dementia is not showering?

The stage of dementia in which a person may have difficulty with personal hygiene, including not showering, can vary and is not necessarily tied to a specific stage of dementia. Personal hygiene challenges, such as difficulty with bathing or showering, can occur in different stages of dementia, depending on the individual's unique circumstances, level of functional ability, and progression of the disease.

For example, in the early stages of dementia, a person may still be able to maintain their personal hygiene routine independently. However, as the disease progresses, they may begin to experience difficulties with executive functions, such as planning, initiating, and completing complex tasks, including bathing or showering. This may be due to memory impairment, confusion, decreased ability to perform self-care tasks, or changes in behavior or personality.

In the middle stages of dementia, a person may require more assistance with personal hygiene, including reminders, cues, and physical assistance with bathing or showering. They may have difficulty with recognizing or remembering the purpose of personal hygiene tasks or may resist or become agitated during these activities.

In the later stages of dementia, a person's functional ability may be significantly impaired, and they may require full assistance with all aspects of personal care, including bathing or showering. At this stage, they may have limited awareness of their personal hygiene needs and may be more dependent on caregivers for all aspects of self-care.

It's important to note that each person with dementia is unique, and the progression of the disease can vary widely. Some individuals with dementia may continue to maintain their personal hygiene routine throughout the various stages of the disease, while others may struggle with personal hygiene tasks early on. It's essential to assess and address each person's individual needs and abilities, and provide appropriate support and assistance with personal hygiene tasks as needed, while respecting their dignity, preferences, and comfort level. Caregivers and healthcare professionals can work together to develop a personalized care plan that addresses the individual's specific needs and stage of dementia.

- 1. How to help someone with dementia with their personal hygiene?

Helping someone with dementia with their personal hygiene can be challenging, as it may require patience, understanding, and sensitivity to the person's cognitive and physical limitations. Here are some tips that may be helpful:

1. Establish a routine: Creating a consistent daily routine for personal hygiene activities, such as bathing or showering, can help establish a sense of familiarity and predictability for the person with dementia. Routine can help reduce confusion and resistance to personal hygiene tasks.
2. Provide visual cues: Use visual cues to help the person understand and follow the steps of personal hygiene tasks. For example, you can use pictures or labels to indicate the order of steps for brushing teeth or washing hands.
3. Simplify the environment: Keep the bathroom or bathing area simple and free from clutter. Reduce any distractions or stimuli that may cause confusion or agitation for the person with dementia.
4. Use gentle and reassuring communication: Use simple and clear language when providing instructions or cues for personal hygiene tasks. Speak in a calm and reassuring tone, and avoid rushing or demanding language, as it may cause the person with dementia to become anxious or resistant.
5. Offer choices and empower independence: Offer choices whenever possible to give the person with dementia a sense of control and autonomy. For example, you can offer choices of soap, shampoo, or towels, or ask them which part of their body they would like to wash first. Encourage them to do as much as they can independently, even if it takes longer or requires assistance.
6. Be mindful of the person's comfort: Consider the person's comfort and preferences when assisting with personal hygiene tasks. Use warm water, adjust the water temperature to their liking, and provide privacy and dignity during the process. Use gentle touch and avoid causing discomfort or pain.
7. Be flexible and patient: Understand that the person with dementia may have good days and bad days, and their ability and willingness to participate in personal hygiene tasks may vary. Be patient, flexible, and compassionate, and try to understand their perspective and feelings.
8. Seek professional help if needed: If you are struggling to help a person with dementia with their personal hygiene, or if the person becomes resistant, agitated, or distressed during personal hygiene tasks, it may be helpful to seek guidance from a healthcare professional, such as a physician, nurse, or occupational therapist, who can provide additional strategies and support.

Remember, every person with dementia is unique, and the approach to assisting with personal hygiene tasks may need to be tailored to their individual needs, abilities, and preferences. It's important to approach personal hygiene tasks with empathy, respect, and patience, and to prioritize the person's comfort, dignity, and well-being throughout the process.

Top of Form

**Questions about food and nutritional information for patients affected with dementia:**

- 1. What are the nutritional needs of patients with dementia?

Patients with dementia have unique nutritional needs that should be addressed to maintain their physical health, cognitive function, and quality of life. As dementia progresses, patients may experience a decrease in appetite, changes in taste and smell, difficulty chewing and swallowing, and other challenges that make it difficult to maintain a balanced diet.

Here are some important nutritional needs for patients with dementia:

1. Adequate hydration: Dehydration is a common problem among dementia patients. Caregivers should encourage patients to drink water and other fluids throughout the day.
2. Sufficient protein: Protein is important for maintaining muscle mass and strength. Caregivers should ensure that patients are getting enough protein in their diet.
3. Fiber-rich foods: Constipation is a common problem among dementia patients. Caregivers should encourage patients to eat foods that are high in fiber, such as fruits, vegetables, and whole grains.
4. Healthy fats: Omega-3 fatty acids found in fish, nuts, and seeds have been shown to improve brain health and cognitive function. Caregivers should include healthy fats in the patient's diet.
5. Vitamins and minerals: Dementia patients may have difficulty getting enough vitamins and minerals from their diet. Caregivers should ensure that patients are getting enough nutrients, either through their diet or through supplements if necessary.

It's important to work with a healthcare professional or registered dietitian to develop a personalized nutrition plan for patients with dementia.

2-How to help someone with dementia with nutrition?

Helping someone with dementia with their nutrition can be challenging due to various cognitive and physical limitations they may experience. Here are some tips on how to assist someone with dementia with their nutrition:

1. Establish a routine: Create a consistent mealtime routine and stick to regular meal and snack times to help establish a sense of structure and familiarity for the person with dementia.
2. Simplify food choices: Offer simple and easy-to-eat foods that are familiar to the person with dementia. Avoid complex or difficult-to-handle foods that may be overwhelming or cause frustration.
3. Provide a balanced diet: Offer a variety of foods from different food groups to ensure the person with dementia is getting a balanced diet that meets their nutritional needs. Include foods that are rich in protein, fiber, healthy fats, vitamins, and minerals.
4. Make meals appealing: Present meals in an appealing way with visually appealing colors, textures, and aromas. Use colorful plates, bowls, and utensils to make the mealtime experience more enjoyable.
5. Assist with eating: Offer assistance with eating as needed. This may include cutting food into small pieces, using utensils to help guide their hand, or providing verbal cues to encourage chewing and swallowing.
6. Offer favorite foods: If the person with dementia has specific food preferences or favorite foods, try to incorporate them into their meals to encourage their appetite and enjoyment of the meal.
7. Monitor hydration: Ensure that the person with dementia is staying adequately hydrated by offering water, juices, soups, and other hydrating fluids throughout the day.
8. Involve the person with dementia: Encourage the person with dementia to participate in meal planning, grocery shopping, and food preparation as much as possible, based on their abilities and interests.
9. Seek professional guidance: Consult with a healthcare professional, such as a registered dietitian, for personalized nutrition recommendations and guidance on meeting the unique nutritional needs of someone with dementia.

It's important to approach nutrition support for someone with dementia with patience, empathy, and understanding of their individual needs and capabilities. Each person with dementia is unique, and their nutritional needs may change as the disease progresses. Working closely with a healthcare professional and involving the person with dementia in decision-making and mealtime activities can help promote their overall health and well-being.

- 1. What are the food restrictions for dementia patients?

There are no specific food restrictions for dementia patients as the nutritional needs and dietary considerations for individuals with dementia are similar to those for the general population. However, there are some general guidelines that can be followed to promote a healthy and balanced diet for individuals with dementia:

1. Limit added sugars and refined carbohydrates: Diets high in added sugars and refined carbohydrates, such as sugary beverages, candies, pastries, and white bread, can contribute to poor nutrition and increase the risk of chronic health conditions. It's important to limit these foods in the diet of someone with dementia.
2. Watch for choking hazards: Dementia patients may have difficulty swallowing, so it's important to avoid foods that can pose a choking hazard, such as hard candies, nuts, large pieces of meat or vegetables, and foods that are difficult to chew or swallow.
3. Be mindful of food allergies and intolerances: Some individuals with dementia may also have food allergies or intolerances, so it's important to be aware of any known allergies or intolerances and avoid those foods accordingly.
4. Consider texture modifications: As dementia progresses, individuals may have difficulty with chewing and swallowing. In such cases, it may be necessary to modify the texture of foods to make them easier to eat. This could include pureeing or softening foods, or providing foods that are naturally soft or easy to swallow, such as soups, stews, mashed potatoes, or smoothies.
5. Consider cultural and personal preferences: Take into consideration the cultural and personal food preferences of the individual with dementia. Offering familiar and preferred foods can help promote appetite and enjoyment of meals.
6. Consider any medical conditions: Consider any coexisting medical conditions, such as diabetes, high blood pressure, or other health conditions that may require dietary modifications. Work with a healthcare professional, such as a registered dietitian, to develop a personalized meal plan that meets the specific nutritional needs of the individual with dementia.

It's important to note that dietary needs may vary for individuals with dementia depending on their overall health status, stage of dementia, and other individual factors. It's recommended to consult with a healthcare professional, such as a registered dietitian or a healthcare provider, for personalized nutrition recommendations and guidance based on the unique needs of the individual with dementia.

- 1. How do you give food and fluids to patients with dementia?

When providing food and fluids to patients with dementia, it's important to consider their individual needs, preferences, and any specific medical conditions they may have. Here are some general guidelines for giving food and fluids to patients with dementia:

1. Create a calm and familiar environment: Patients with dementia may feel anxious or confused during meal times. Creating a calm and familiar environment can help reduce stress and encourage eating. Reduce distractions such as loud noises or bright lights, and create a peaceful atmosphere.
2. Maintain a consistent meal routine: Establishing a consistent meal routine can help patients with dementia develop a sense of familiarity and predictability. Serve meals at the same time each day and try to stick to a consistent schedule.
3. Offer a variety of foods: Provide a balanced and nutritious diet that includes a variety of foods from different food groups. Offer foods that the patient enjoys and is familiar with, but also introduce new foods to provide a balanced diet.
4. Adapt food textures: Depending on the patient's ability to chew and swallow, you may need to adapt the texture of food. Some patients with dementia may have difficulty with solid foods, and may require pureed or soft foods that are easier to swallow. Consult with a healthcare professional, such as a registered dietitian, for guidance on appropriate food textures.
5. Use appropriate utensils: Provide utensils that are easy to use for the patient, such as spoons with large handles or forks with bent tines for better grip. Adapt the utensils based on the patient's dexterity and ability to handle them.
6. Offer fluids regularly: Dehydration can be a concern for patients with dementia, as they may forget to drink enough fluids. Offer fluids regularly throughout the day, and encourage the patient to take sips of water or other hydrating beverages.
7. Be patient and provide assistance: Patients with dementia may have difficulty feeding themselves or may exhibit behaviors such as food refusal or agitation during meals. Be patient and provide assistance as needed. Offer gentle prompts and cues, and be attentive to the patient's cues and signals.
8. Involve the patient in the process: Encourage the patient to participate in the mealtime process as much as possible. This may include helping with setting the table, choosing foods, or even preparing simple meals with supervision. Involving the patient in the process can promote a sense of independence and engagement.
9. Communicate with the healthcare team: Regularly communicate with the patient's healthcare team, including caregivers, nurses, and registered dietitians, to ensure that the patient's nutritional needs are being met and to address any concerns or changes in the patient's eating habits.

Remember that each patient with dementia is unique, and it's important to tailor the approach to their individual needs and abilities. Consult with healthcare professionals for personalized recommendations and guidance on providing food and fluids to patients with dementia.

**Questions about behavior and Safety issues of patients affected with dementia:**

- 1. How do you deal with Alzheimer's patients?

Caring for patients with Alzheimer's disease requires compassion, patience, and understanding. Here are some general tips for dealing with Alzheimer's patients:

1. Educate yourself about Alzheimer's disease: Learn about the symptoms, progression, and challenges associated with Alzheimer's disease. This will help you better understand and empathize with the patient's condition.
2. Create a safe and supportive environment: Ensure that the physical environment is safe and free from hazards. Minimize clutter, provide good lighting, and remove any potential dangers. Create a calming and familiar environment that promotes a sense of security for the patient.
3. Establish a routine: Routine can provide a sense of predictability and familiarity for Alzheimer's patients. Establish a consistent daily routine for activities such as meals, medication, and personal care. Keep the routine simple and flexible, and be prepared to adapt as needed.
4. Practice effective communication: Communicate with Alzheimer's patients in a clear and simple manner. Use simple language, speak slowly, and use non-verbal cues such as gestures and facial expressions. Avoid speaking in a condescending or patronizing manner.
5. Practice active listening: Pay attention to the patient's cues, emotions, and non-verbal communication. Listen patiently and respectfully to their words, even if they may not make complete sense. Respond with empathy and reassurance.
6. Be patient and flexible: Alzheimer's patients may exhibit behaviors such as agitation, confusion, or repetitive actions. Be patient and understanding, and avoid reacting with frustration or anger. Be flexible and adapt to the patient's changing needs and abilities.
7. Encourage independence: Promote the patient's independence and autonomy as much as possible. Allow them to participate in activities that they are capable of doing, even if it takes longer or requires assistance. Encourage decision-making within their abilities.
8. Provide meaningful activities: Engage Alzheimer's patients in activities that they enjoy and that provide meaning and purpose. This could include hobbies, crafts, music, or gentle exercises. Tailor the activities to the patient's interests and abilities.
9. Seek support: Caring for patients with Alzheimer's disease can be emotionally and physically challenging. Seek support from family members, friends, or support groups. Consider consulting with healthcare professionals for guidance and assistance.
10. Take care of yourself: Caring for Alzheimer's patients can be demanding, so it's important to prioritize self-care. Make sure to take breaks, get adequate rest, eat well, and seek support when needed. Taking care of yourself will help you provide better care for the patient.

Remember that Alzheimer's disease affects each person differently, and what works for one patient may not work for another. It's important to approach each situation with empathy, patience, and flexibility, and to seek guidance from healthcare professionals as needed.

- 1. What not to do with a dementia patient?

Caring for a dementia patient requires careful consideration of their unique needs and sensitivities. Here are some things to avoid when dealing with a dementia patient:

1. Don't argue or correct: Dementia patients may experience confusion or memory loss, and arguing or correcting them can cause distress or agitation. Avoid insisting that they are wrong or correcting their statements. Instead, respond with empathy and reassurance, focusing on their emotions and well-being.
2. Don't use complex language or jargon: Using complicated language or jargon can confuse dementia patients further. Use simple and clear language, speak slowly, and use non-verbal cues to aid communication.
3. Don't rush or impose tasks: Dementia patients may require more time to complete tasks or may not be able to do them at all. Avoid rushing or imposing tasks on them. Allow them to do what they can at their own pace, and provide assistance when needed.
4. Don't underestimate safety risks: Dementia patients may have impaired judgment or reduced safety awareness. Avoid underestimating safety risks and ensure that the environment is safe and free from hazards. Take appropriate measures to prevent falls, accidents, or other potential dangers.
5. Don't use physical or verbal aggression: Dementia patients may exhibit challenging behaviors, but using physical or verbal aggression in response is not acceptable. Avoid using force, yelling, or harsh language. Instead, remain calm, use a reassuring tone, and try to de-escalate the situation.
6. Don't neglect self-care: Caring for a dementia patient can be emotionally and physically demanding. Avoid neglecting your own self-care, as it can impact your ability to provide effective care. Make sure to prioritize your own well-being, seek support, and take breaks when needed.
7. Don't isolate or neglect social interaction: Social interaction is important for dementia patients' well-being. Avoid isolating them or neglecting social interaction. Encourage meaningful activities, engagement with others, and opportunities for socialization, which can promote cognitive function and emotional well-being.
8. Don't make assumptions or generalizations: Each dementia patient is unique, and it's important to avoid making assumptions or generalizations about their abilities or limitations. Assess their individual needs, abilities, and preferences, and provide care that is tailored to their specific requirements.
9. Don't neglect proper nutrition and hydration: Dementia patients may have difficulty with eating or drinking, leading to malnutrition or dehydration. Avoid neglecting proper nutrition and hydration. Offer regular meals and fluids, adapt food textures as needed, and consult with a registered dietitian for guidance on meeting their nutritional needs.
10. Don't neglect seeking professional help: Dementia care can be complex, and it's important to seek professional help when needed. Don't hesitate to consult with healthcare professionals, including physicians, nurses, social workers, or occupational therapists, for guidance, support, and assistance in providing optimal care for the dementia patient.

Remember, caring for a dementia patient requires patience, empathy, and understanding. Avoiding these common mistakes can help provide compassionate and effective care for individuals with dementia, while also ensuring your own well-being as a caregiver.

Top of Form

- 1. How can you maintain the safety of dementia patients?

Maintaining the safety of dementia patients is crucial to prevent accidents, injuries, and other risks associated with their cognitive impairment. Here are some tips for maintaining the safety of dementia patients:

1. Create a safe environment: Ensure that the physical environment is safe by removing hazards such as clutter, loose rugs, and sharp objects. Install handrails in bathrooms and staircases, use non-slip mats, and ensure proper lighting to prevent falls.
2. Use safety devices: Utilize safety devices such as door locks, window locks, and safety gates to prevent the dementia patient from wandering or accessing unsafe areas. Consider using monitoring devices or wearable technology that can alert you if the patient leaves a designated area.
3. Supervise and monitor: Provide constant supervision and monitoring, especially if the dementia patient is at risk of wandering or engaging in unsafe behaviors. Keep an eye on them during activities such as cooking, bathing, or using household appliances.
4. Adapt the environment: Modify the environment to meet the specific needs of the dementia patient. For example, use visual cues such as signs, labels, and color-coded items to help with orientation and navigation. Use clocks or calendars to assist with time orientation.
5. Provide clear communication: Use simple and clear communication when interacting with the dementia patient. Avoid complex language or jargon that may confuse or frustrate them. Use visual aids, gestures, and other non-verbal cues to aid communication.
6. Establish a routine: Establish a consistent routine for daily activities, such as mealtimes, medication schedules, and bedtime routines. A predictable routine can help reduce confusion and agitation in dementia patients.
7. Promote mobility and exercise: Encourage regular physical activity and exercise, as it can help maintain muscle strength, balance, and coordination, which can reduce the risk of falls and other accidents.
8. Monitor and manage medications: Ensure that medications are managed safely, with proper dosages, frequency, and administration. Keep medications out of reach and monitor for any adverse reactions or interactions.
9. Provide proper nutrition and hydration: Ensure that the dementia patient receives adequate nutrition and hydration. Adapt food textures or consistencies as needed, and offer regular meals and fluids to prevent malnutrition and dehydration.
10. Involve healthcare professionals: Involve healthcare professionals, including physicians, nurses, and occupational therapists, for assessments, recommendations, and interventions to optimize the safety and well-being of the dementia patient.

Remember that dementia patients may have changing needs and abilities over time, so regularly reassess the safety of their environment and adjust care accordingly. By implementing these measures, you can help maintain the safety of dementia patients and reduce the risks associated with their cognitive impairment.

- 1. How can you help a client with dementia feel safe and secure during care?

Helping a client with dementia feel safe and secure during care is important to reduce anxiety, agitation, and other behavioral symptoms associated with the condition. Here are some strategies that can help:

1. Establish trust and rapport: Building a trusting relationship with the client is essential. Approach them with warmth, kindness, and empathy. Use their preferred name and address them respectfully to create a sense of familiarity and trust.
2. Use familiar and comforting items: Surround the client with familiar items from their past, such as photographs, personal belongings, or familiar scents. These familiar items can provide a sense of security and comfort.
3. Maintain a consistent routine: Stick to a consistent routine for care activities, such as bathing, dressing, and meal times. Consistency can help the client feel more secure and reduce confusion or distress.
4. Provide a calm and soothing environment: Create a calm and soothing environment by reducing noise, clutter, and distractions. Use soft lighting and play calming music to create a relaxing atmosphere.
5. Use non-verbal communication: Non-verbal communication, such as gentle touch, eye contact, and facial expressions, can help convey reassurance and comfort to the client. Pay attention to their non-verbal cues and respond accordingly.
6. Avoid correcting or arguing: Refrain from correcting or arguing with the client, as it can lead to frustration and agitation. Instead, validate their feelings and emotions, and redirect their attention to positive or enjoyable activities.
7. Provide clear and simple instructions: Use clear and simple instructions when providing care. Break down tasks into small steps and use visual cues or gestures to help the client understand what is happening.
8. Offer choices: Offer the client choices whenever possible to help them maintain a sense of autonomy and control. For example, let them choose between two clothing options or offer choices during mealtime.
9. Use distraction and redirection: If the client becomes agitated or anxious, use distraction and redirection techniques to shift their focus to a different activity or topic. This can help defuse a potentially challenging situation and promote a sense of calm.
10. Involve family and familiar caregivers: Involving family members or familiar caregivers in the care process can help the client feel more secure and comfortable. Familiar faces and voices can provide a sense of continuity and stability.

Remember, each person with dementia is unique, and what works for one person may not work for another. It's important to be patient, flexible, and attuned to the individual needs and preferences of the client. Consulting with healthcare professionals, including dementia specialists or caregivers, can also provide valuable guidance and support in providing care that promotes a sense of safety and security for clients with dementia.

Top of Form

- 1. Why is safety needed for dementia patients?

Safety is crucial for dementia patients due to the cognitive impairments and behavioral changes that are commonly associated with dementia. Here are some reasons why safety is important for dementia patients:

1. Risk of falls: Dementia patients may experience problems with balance, coordination, and judgment, which can increase their risk of falls. Falls can result in serious injuries, such as fractures or head injuries, which can have severe consequences for the physical health and well-being of dementia patients.
2. Wandering and getting lost: Many individuals with dementia may wander and become disoriented, which can lead to getting lost and being exposed to various risks, such as environmental hazards, extreme weather conditions, or dangerous situations. Wandering can also cause distress to both the person with dementia and their caregivers.
3. Difficulty with decision-making and judgment: Dementia can impair a person's ability to make sound decisions and exercise good judgment. This can result in unsafe behaviors, such as forgetting to turn off appliances, leaving the stove on, or engaging in risky activities.
4. Reduced awareness of danger: Dementia can diminish a person's ability to recognize or respond appropriately to dangerous situations. For example, they may not recognize the danger of touching hot surfaces, ingesting harmful substances, or interacting with strangers.
5. Changes in behavior and agitation: Dementia can cause changes in behavior, including agitation, aggression, and impulsivity. These behaviors can pose risks to both the person with dementia and those around them, including caregivers, family members, and other residents in a care setting.
6. Medication management issues: Dementia patients may have difficulty managing their medications properly, leading to potential medication errors, over-dosage, or adverse drug interactions, which can compromise their safety and well-being.
7. Communication difficulties: Dementia can impair communication skills, making it challenging for individuals with dementia to express their needs or understand instructions. This can result in misunderstandings or misinterpretations, leading to potential safety risks.

Due to these cognitive impairments and behavioral changes associated with dementia, ensuring safety measures are in place is crucial to minimize the risks and promote the well-being of dementia patients. Implementing safety measures can help prevent accidents, injuries, wandering, and other potentially harmful situations, and provide a supportive and secure environment for individuals with dementia.

**Questions about Coping with hallucinations:**

- 1. How do you deal with someone who hallucinates from dementia?

Dealing with someone who hallucinates from dementia requires a compassionate and patient approach. Here are some strategies to consider:

1. Stay calm and reassuring: It's important to remain calm and composed when dealing with someone who is experiencing hallucinations. Avoid expressing disbelief or arguing about the hallucinations, as this can escalate the situation. Instead, reassure the person and provide a calming presence.
2. Validate their experiences: Acknowledge and validate the person's experiences, even if they seem illogical or unreal. Avoid trying to convince them that their hallucinations are not real, as this can cause distress or agitation. Instead, use empathetic and validating statements, such as "That sounds scary" or "I understand that you are seeing something."
3. Provide a safe environment: Ensure that the environment is safe and free from any potential hazards related to the hallucinations. For example, if the person is seeing objects that are not there, gently guide them around the objects or remove them from their sight to prevent accidents.
4. Offer reassurance and comfort: Provide reassurance and comfort to the person by offering physical touch, such as holding their hand or giving them a hug, if appropriate and welcomed by the individual. Use soothing and comforting words to help calm them down.
5. Redirect their attention: If the person is becoming agitated or distressed due to their hallucinations, try redirecting their attention to a different activity or topic. This can help shift their focus away from the hallucinations and reduce their distress.
6. Avoid confrontation or correction: It's important to avoid confronting or correcting the person about their hallucinations, as this can lead to increased agitation or confusion. Instead, focus on providing comfort and reassurance, and avoid challenging or contradicting their perceptions.
7. Involve healthcare professionals: If the hallucinations are persistent or causing significant distress, involve healthcare professionals, such as the person's primary care physician or a dementia specialist. They can provide further assessment, guidance, and potential medication interventions, if appropriate.
8. Document and report: It's essential to document and report any instances of hallucinations to the person's healthcare team, caregivers, and family members. This can help in tracking patterns, identifying triggers, and developing strategies to manage the hallucinations effectively.

Dealing with hallucinations in dementia patients can be challenging, and it's important to approach the situation with empathy, patience, and understanding. Consulting with healthcare professionals or seeking support from dementia caregivers or support groups can provide valuable guidance and assistance in managing hallucinations in individuals with dementia.

- 1. What are the coping strategies for dementia patients?

Coping strategies can be helpful for individuals with dementia to manage their symptoms and maintain a better quality of life. Here are some coping strategies for dementia patients:

1. Establish a routine: Maintaining a consistent daily routine can help provide structure and stability for individuals with dementia. Establishing regular schedules for daily activities such as meals, medication, exercise, and leisure time can help create a sense of predictability and familiarity, which may reduce anxiety and confusion.
2. Simplify the environment: Simplify the environment by reducing clutter, removing unnecessary items, and creating clear pathways to minimize confusion and improve safety. Using labels, signs, or color-coded cues can also help individuals with dementia identify and navigate their surroundings.
3. Use memory aids: Memory aids, such as calendars, clocks, and written reminders, can be useful in helping individuals with dementia remember important information, appointments, and tasks. Technology-based memory aids, such as reminder apps or voice-activated devices, can also be beneficial.
4. Engage in meaningful activities: Encouraging engagement in meaningful activities, such as hobbies, art, music, and reminiscing, can help stimulate cognitive function, enhance mood, and reduce agitation in individuals with dementia. Tailor activities to the person's interests, abilities, and preferences.
5. Provide social support: Social interaction and emotional support are vital for individuals with dementia. Regular visits from family members, friends, or caregivers can provide companionship, emotional connection, and stimulation. Participation in social programs, support groups, or dementia-specific activities can also be beneficial.
6. Practice good communication: Using simple and clear language, speaking slowly and calmly, and providing visual cues can enhance communication with individuals with dementia. Avoid using complex or ambiguous language, talking loudly, or speaking in a condescending manner. Non-verbal communication, such as gestures, facial expressions, and touch, can also convey messages effectively.
7. Promote physical health: Maintaining physical health can help manage dementia symptoms. Encouraging regular exercise, healthy eating, and adequate sleep can improve overall well-being, reduce behavioral symptoms, and enhance cognitive function.
8. Seek professional support: Consulting with healthcare professionals, such as geriatricians, neurologists, or dementia specialists, can provide valuable guidance and support in managing dementia symptoms. Medication interventions, behavioral therapies, and other interventions may be recommended to manage specific symptoms.
9. Provide a supportive environment: Creating a supportive and compassionate environment is crucial for individuals with dementia. Offering reassurance, empathy, and patience, and avoiding confrontation, criticism, or correction can help reduce distress and improve overall well-being.

Coping strategies for dementia patients may vary depending on the stage of dementia, individual needs, and abilities. It's important to work with healthcare professionals, caregivers, and support networks to develop a personalized approach that meets the unique needs of the person with dementia.

- 1. What stage of dementia are hallucinations?

1. Hallucinations can occur in various stages of dementia, but they are more commonly associated with the later stages of the disease. Hallucinations are often considered a symptom of moderate to severe dementia and can be particularly prevalent in conditions such as Alzheimer's disease with Lewy bodies (AD-LB), which is a form of dementia characterized by the presence of abnormal protein deposits in the brain called Lewy bodies.
2. In the early stages of dementia, individuals may experience mild cognitive symptoms, such as memory loss and difficulty with concentration, but hallucinations are less common. However, as dementia progresses, and the brain undergoes more significant changes, including damage to areas responsible for perception and processing of sensory information, hallucinations may become more frequent.
3. In the later stages of dementia, which are often characterized by severe cognitive decline, impaired communication, and decreased ability to recognize familiar people or places, hallucinations may become more pronounced. The type of hallucinations experienced by individuals with dementia can vary, but they often involve visual hallucinations, such as seeing people, animals, or objects that are not present.
4. It's important to note that hallucinations in dementia can be distressing for both the individual with dementia and their caregivers, and it's essential to seek appropriate medical and professional support to manage and address these symptoms effectively. Healthcare professionals, such as geriatricians, neurologists, or dementia specialists, can provide guidance on appropriate interventions, including medication management, behavioral strategies, and supportive care to help manage hallucinations in individuals with dementia.

4- How do you calm hallucinations in the elderly?

Coping with hallucinations in the elderly, particularly in individuals with dementia, can be challenging. Here are some strategies that may help calm hallucinations in the elderly:

1. Provide reassurance: Offering reassurance and comfort to the individual experiencing hallucinations can be helpful. Speak calmly, use a soothing tone of voice, and provide validation and comfort without dismissing or contradicting their perceptions.
2. Validate their experiences: Avoid denying or arguing with the person's hallucinations, as this may cause increased agitation or distress. Instead, try to validate their experiences by acknowledging their feelings and perceptions. You can say things like, "That sounds frightening," or "I understand that you're seeing something that I can't see."
3. Create a calm environment: Creating a calm and soothing environment can help reduce anxiety and agitation associated with hallucinations. Dimming lights, playing soft music, and removing any potential triggers or distractions from the environment may be beneficial.
4. Distraction techniques: Redirecting the individual's attention to a different topic or activity can be effective in managing hallucinations. Engaging in activities that the person enjoys, such as listening to music, looking at old photos, or engaging in a familiar hobby, can help shift their focus away from the hallucinations.
5. Maintain a regular routine: Establishing a consistent daily routine can provide structure and familiarity, which may help reduce confusion and agitation associated with hallucinations. Regular routines for meals, medication, and activities can help create a sense of predictability and stability.
6. Medication management: In some cases, medication may be prescribed by a healthcare professional to manage hallucinations in the elderly. Antipsychotic medications or other medications may be used under the guidance of a qualified healthcare professional, but their use should be carefully considered and monitored due to potential side effects.
7. Involve healthcare professionals: It's important to involve healthcare professionals, such as geriatricians, neurologists, or dementia specialists, in managing hallucinations in the elderly. They can provide guidance on appropriate interventions, including medication management, behavioral strategies, and supportive care tailored to the individual's needs.
8. Ensure safety: If the individual experiencing hallucinations becomes agitated, disoriented, or potentially harmful to themselves or others, it's important to prioritize safety. Take steps to ensure the person's safety and that of those around them, which may involve removing any potentially dangerous objects, providing supervision, or seeking professional help as needed.

It's essential to work closely with healthcare professionals and caregivers to develop an individualized care plan that addresses the specific needs and challenges associated with hallucinations in the elderly, particularly in the context of dementia. Each person's experience with hallucinations may be different, and the management approach should be tailored to their unique needs and condition.

**Communication difficulties and how to manage patients wuth dementia:**

- 1. What are 3 common communication difficulties for people with dementia?

Communicating with individuals who have dementia can present several challenges due to the progressive cognitive decline and changes in language abilities that are characteristic of the condition. Here are three common communication difficulties that people with dementia may experience:

1. Difficulty with finding the right words: As dementia progresses, individuals may struggle to find the right words to express their thoughts or convey their needs. They may experience word-finding difficulties or have trouble remembering the names of common objects or people. This can lead to frustration, confusion, and difficulty in expressing themselves effectively.
2. Impaired comprehension: Individuals with dementia may have difficulty understanding spoken or written language. They may have trouble following instructions, comprehending questions, or understanding complex sentences. This can result in misunderstandings, misinterpretations, and miscommunication, leading to frustration and confusion for both the individual with dementia and their caregivers.
3. Disorientation and confusion: People with dementia may experience disorientation and confusion, particularly regarding time, place, and person. They may lose track of the current date, forget where they are or whom they are speaking with, and may have difficulty recalling recent events or past memories accurately. This can lead to misunderstandings and communication breakdowns, as their perception of reality may be altered.

It's important to keep in mind that communication difficulties in dementia are progressive and may vary depending on the stage and type of dementia. It's crucial to be patient, understanding, and adaptive in your communication approach when interacting with individuals with dementia. Using simple language, speaking slowly and clearly, using visual aids, and using non-verbal cues, such as gestures and facial expressions, can be helpful strategies to improve communication with individuals who have dementia. It's also important to actively listen, be empathetic, and show understanding to help mitigate communication challenges and facilitate effective communication with individuals with dementia. Consulting with healthcare professionals, including speech-language pathologists or dementia specialists, can also provide valuable guidance and support in managing communication difficulties in individuals with dementia.

- 1. What can help improve communication with a patient who has dementia?

Improving communication with a patient who has dementia requires understanding and adapting to their unique needs and abilities. Here are some strategies that may help improve communication with a person who has dementia:

1. Use simple language: Use simple words and short sentences to convey your message. Avoid using complex or technical language, and try to break down information or instructions into smaller, manageable parts. Speak slowly and clearly, and allow time for the person to process and respond.
2. Provide visual aids: Use visual aids, such as pictures, diagrams, or written notes, to support your verbal communication. Visual cues can help individuals with dementia better understand and remember information. Pointing to objects or using gestures can also be helpful in reinforcing your message.
3. Be patient and give them time: Individuals with dementia may require more time to process information and respond. Be patient and avoid rushing or interrupting. Allow them to express themselves at their own pace, and resist the urge to finish their sentences or speak for them.
4. Use non-verbal cues: Non-verbal cues, such as facial expressions, tone of voice, and body language, can convey meaning and emotions. Use a calm and reassuring tone of voice, and maintain eye contact to establish connection and trust.
5. Be empathetic and validate feelings: Validate the person's feelings and emotions, even if their perceptions or memories may not be accurate. Offer empathy, understanding, and reassurance. Avoid correcting or arguing with their statements, as this may cause further confusion or agitation.
6. Create a supportive environment: Minimize distractions and noise in the environment to help the person focus on the conversation. Ensure that the lighting is adequate, and reduce any environmental factors that may trigger agitation or confusion.
7. Use familiar and meaningful topics: Use familiar and meaningful topics of conversation, such as past memories, hobbies, or interests, to engage the person with dementia. This can help stimulate their memory and encourage communication.
8. Involve sensory cues: Sensory cues, such as touch, smell, or taste, can help stimulate memories and create a connection. For example, holding their hand, offering a favorite food or drink, or using familiar scents can evoke positive memories and emotions.
9. Practice active listening: Practice active listening by giving your full attention, maintaining eye contact, and responding appropriately to the person's cues and messages. Show genuine interest and curiosity in their communication, and avoid interrupting or finishing their sentences.
10. Seek professional help: Consult with healthcare professionals, such as speech-language pathologists or dementia specialists, for further guidance and support in improving communication with individuals with dementia. They can provide tailored strategies and interventions based on the person's specific needs and condition.

Remember that every person with dementia is unique, and what works for one person may not work for another. It's essential to be flexible, patient, and empathetic in your communication approach, and to constantly assess and adapt to the person's changing abilities and needs. Effective communication can help improve their quality of life, reduce agitation and frustration, and enhance their sense of connection and engagement with their environment and caregivers.

- 1. What types of actions can make communicating with residents with dementia difficult?

Communicating with residents with dementia can be challenging due to various factors. Here are some common actions or situations that can make communication difficult:

1. Cognitive impairment: Dementia affects cognitive functions such as memory, attention, problem-solving, and language. Residents with dementia may have difficulty understanding, processing, or recalling information, which can affect their ability to communicate effectively.
2. Language difficulties: Dementia can impair language skills, including speaking, understanding, reading, and writing. Residents may struggle to find the right words, follow a conversation, or express their thoughts coherently.
3. Emotional distress: Residents with dementia may experience emotional distress, including anxiety, agitation, or depression, which can impact their ability to communicate. They may become overwhelmed, confused, or agitated, leading to difficulty in expressing themselves or understanding others.
4. Sensory impairments: Sensory impairments, such as hearing or vision loss, can further complicate communication with residents with dementia. They may not be able to hear or see well, which can affect their ability to understand spoken words or interpret visual cues.
5. Environmental factors: Environmental factors, such as noise, distractions, or unfamiliar surroundings, can make communication challenging for residents with dementia. A noisy or cluttered environment can be overwhelming and distracting, making it difficult for them to focus or follow a conversation.
6. Lack of familiarity: Lack of familiarity with the caregiver, environment, or topic of conversation can also hinder effective communication. Residents with dementia may struggle to connect with unfamiliar caregivers or understand new or unfamiliar situations, which can impact their ability to communicate.
7. Emotional attachment to a topic: Residents with dementia may become emotionally attached to certain topics, memories, or perceptions, and may insist on their version of reality, even if it contradicts with the facts. This can make communication difficult as they may resist or become agitated when their beliefs or memories are challenged.
8. Physical limitations: Physical limitations, such as weakness, tremors, or difficulty with fine motor skills, can affect the ability of residents with dementia to express themselves or engage in communication activities, such as writing or gesturing.
9. Lack of context or cues: Communication may be challenging if residents with dementia lack the context or cues necessary to understand the conversation. They may struggle to interpret abstract or complex information, sarcasm, or non-literal language.
10. Caregiver approach: The approach and communication style of the caregiver can also impact the communication with residents with dementia. If the caregiver is impatient, dismissive, or confrontational, it can create barriers to effective communication and may cause the resident to become more resistant or agitated.

It's important to recognize and address these factors to improve communication with residents with dementia. Using strategies such as simplifying language, using visual cues, creating a supportive environment, being patient and empathetic, and adapting to the individual's needs and abilities can help facilitate effective communication and enhance the overall care and well-being of residents with dementia.

4- Why communication is difficult with dementia?

Communication can be difficult for individuals with dementia due to the progressive cognitive decline that is characteristic of the condition. Dementia affects various cognitive functions, including memory, attention, problem-solving, language, and perception, which can impair communication in several ways:

1. Language difficulties: Dementia can affect language skills, including speaking, understanding, reading, and writing. Individuals with dementia may have difficulty finding the right words, following a conversation, or expressing their thoughts coherently.
2. Impaired comprehension: Dementia can impact an individual's ability to comprehend spoken or written language. They may have difficulty understanding questions, following instructions, or interpreting complex information.
3. Reduced verbal expression: Dementia can result in reduced verbal expression, where individuals may have limited vocabulary, struggle with word finding, or speak in shorter and less complex sentences.
4. Cognitive processing deficits: Dementia can impair cognitive processing, making it challenging for individuals to process and organize information, respond to questions, or engage in meaningful conversations.
5. Disorientation: Individuals with dementia may experience disorientation, confusion, or memory loss, which can affect their ability to engage in coherent communication or follow the flow of a conversation.
6. Emotional distress: Dementia can cause emotional distress, including anxiety, agitation, or depression, which can further impact communication. Emotional distress can make individuals more resistant, agitated, or withdrawn, hindering effective communication.
7. Lack of context or cues: Individuals with dementia may struggle to interpret abstract or complex information, sarcasm, or non-literal language, as they may have difficulty understanding the context or cues necessary for effective communication.
8. Environmental factors: Environmental factors, such as noise, distractions, or unfamiliar surroundings, can make communication challenging for individuals with dementia. A noisy or cluttered environment can be overwhelming and distracting, making it difficult for them to focus or follow a conversation.
9. Lack of self-awareness: Individuals with dementia may have limited self-awareness and may not recognize their communication difficulties. They may not be able to express their needs or understand that their communication is impaired, which can further hinder effective communication.
10. Caregiver approach: The approach and communication style of the caregiver can also impact communication with individuals with dementia. If the caregiver is not patient, empathetic, or skilled in communicating with dementia patients, it can create barriers to effective communication.

It's important to understand these challenges and use strategies tailored to the individual's needs and abilities to improve communication with individuals with dementia. Simplifying language, using visual cues, creating a supportive environment, being patient and empathetic, and adapting communication techniques can help facilitate effective communication and enhance the overall care and well-being of individuals with dementia.

**Daily activities and patients affected with dementia:**

1. What are daily activities in dementia?

Daily activities for individuals with dementia should be tailored to their abilities, interests, and preferences, as well as the stage of dementia they are in. Here are some examples of daily activities that can be beneficial for individuals with dementia:

1. Personal care: Assisting with personal hygiene activities such as bathing, grooming, and dressing can help maintain the individual's physical health and well-being. It is essential to approach these activities with sensitivity, respect, and dignity, and adapt them to the individual's level of ability and comfort.
2. Exercise and physical activities: Encouraging regular physical exercise, such as walking, gentle stretching, or chair exercises, can help maintain mobility, strength, and balance. Physical activities can also improve mood, reduce agitation, and promote overall well-being.
3. Cognitive activities: Engaging in cognitive activities, such as puzzles, memory games, reading, or engaging in hobbies or crafts, can help stimulate the brain, promote cognitive function, and provide a sense of accomplishment and purpose.
4. Social activities: Encouraging social interactions, such as conversation, reminiscing, or participating in group activities, can help reduce social isolation and promote social and emotional well-being. Activities that involve interacting with others, such as music therapy, art therapy, or group exercises, can be particularly beneficial.
5. Music and sensory activities: Listening to familiar music, engaging in sensory activities such as touching different textures, or using sensory objects like scented items or soft blankets, can help calm and engage individuals with dementia.
6. Household tasks: Involving individuals in simple household tasks, such as setting the table, folding laundry, or watering plants, can provide a sense of purpose and accomplishment. These tasks can be adapted to the individual's abilities and preferences.
7. Outdoor activities: Spending time outdoors, such as taking a walk in a garden or sitting in a park, can provide fresh air, sunlight, and sensory stimulation, which can be beneficial for individuals with dementia.
8. Reminiscence activities: Engaging in reminiscence activities, such as looking at old photos, sharing stories, or talking about past experiences, can help stimulate memory, encourage conversation, and promote emotional well-being.
9. Relaxation activities: Engaging in relaxation activities, such as deep breathing, meditation, or gentle massage, can help reduce anxiety, agitation, and promote relaxation and calmness.

It's important to assess the individual's abilities, interests, and preferences and adapt daily activities accordingly. It's also crucial to provide a structured and predictable routine, be patient, and provide emotional support to individuals with dementia during daily activities to enhance their overall well-being and quality of life.

1. Why are activities important for dementia patients?

Activities are important for individuals with dementia for several reasons:

1. Cognitive stimulation: Engaging in various activities can help stimulate the brain and promote cognitive function. Activities that challenge memory, problem-solving, and decision-making skills can help maintain cognitive abilities and slow the progression of cognitive decline in individuals with dementia.
2. Emotional well-being: Activities can provide a sense of purpose, accomplishment, and satisfaction, which can boost the emotional well-being of individuals with dementia. Meaningful activities that align with their interests, preferences, and abilities can help improve mood, reduce anxiety, depression, and other behavioral symptoms associated with dementia.
3. Quality of life: Engaging in enjoyable and meaningful activities can enhance the overall quality of life for individuals with dementia. Activities can provide a sense of structure, routine, and normalcy, which can help individuals feel more in control and connected to the world around them.
4. Social interaction: Many activities can be done in a social setting, such as group exercises, games, or hobbies, which can help promote social interaction and reduce social isolation. Social engagement can help improve mood, reduce agitation, and promote emotional well-being in individuals with dementia.
5. Physical well-being: Certain activities, such as exercise, mobility tasks, and sensory activities, can help maintain physical health and well-being in individuals with dementia. Physical activities can promote mobility, strength, balance, and overall physical fitness, which can help maintain independence and reduce the risk of falls and other health complications.
6. Behavior management: Engaging individuals with dementia in meaningful activities can also help redirect their energy and focus, reducing agitation, restlessness, and other challenging behaviors. Activities can provide a positive outlet for excess energy and help individuals with dementia feel more calm and relaxed.

Overall, activities play a crucial role in improving the well-being and quality of life of individuals with dementia. It's important to assess the individual's abilities, interests, and preferences and adapt activities accordingly, while providing supervision, support, and encouragement to help individuals with dementia engage in activities that are safe, enjoyable, and meaningful to them.

1. What activities can people with dementia or Alzheimer's do?

There are many different activities that individuals with dementia or Alzheimer's can engage in, depending on their abilities, interests, and preferences. Here are some examples:

1. Memory games: Memory games, such as matching games, puzzles, and card games, can help stimulate cognitive function, memory, and concentration.
2. Reminiscence activities: Reminiscence activities involve recalling and sharing memories from the past. This can include looking at old photos, listening to familiar music, or talking about past events or experiences.
3. Sensory activities: Sensory activities can engage the senses, such as touch, smell, taste, sight, and hearing. Examples include sensory boxes with different textures, aromatherapy, or listening to calming music.
4. Arts and crafts: Engaging in arts and crafts activities, such as painting, drawing, coloring, or crafting, can provide a creative outlet and promote self-expression.
5. Physical activities: Physical activities, such as gentle exercises, walking, dancing, or stretching, can help promote physical health, mobility, and well-being.
6. Music therapy: Music can be a powerful tool for individuals with dementia. Listening to familiar music, singing, or playing musical instruments can help improve mood, reduce anxiety, and promote relaxation.
7. Pet therapy: Interacting with animals, such as therapy dogs or cats, can provide comfort, companionship, and emotional support for individuals with dementia.
8. Simple household tasks: Engaging in simple household tasks, such as folding laundry, setting the table, or sorting items, can provide a sense of purpose and accomplishment.
9. Social activities: Social activities, such as group discussions, social games, or social outings, can promote social interaction and reduce social isolation.
10. Outdoor activities: Spending time outdoors, such as gardening, walking in nature, or sitting in a park, can provide fresh air, sunlight, and a change of scenery, which can be beneficial for individuals with dementia.

It's important to adapt activities based on the individual's abilities, interests, and preferences, and to provide supervision, support, and encouragement as needed. Consulting with a healthcare professional or a dementia care specialist can help identify appropriate activities and strategies for engaging individuals with dementia or Alzheimer's in meaningful and enjoyable activities.

**First aid for patient with dementia:**

- 1. What is the first aid for dementia patients?

First aid for individuals with dementia or Alzheimer's disease may vary depending on the specific situation and the severity of the condition. Here are some general guidelines for providing first aid for dementia patients:

1. Assess the situation: Check for any immediate dangers or hazards in the environment, such as tripping hazards, sharp objects, or unsafe conditions. Ensure that the person is safe from any potential harm.
2. Stay calm and reassuring: Individuals with dementia may become confused, agitated, or anxious during a medical emergency. It's important to stay calm, speak in a reassuring tone, and provide comfort and reassurance to the person.
3. Call for medical assistance: If the individual is experiencing a medical emergency, such as difficulty breathing, chest pain, severe injury, or loss of consciousness, call emergency services (e.g., 911) for professional medical assistance.
4. Provide comfort and reassurance: Offer comfort and reassurance to the person, and try to maintain a calm and soothing demeanor. Use simple and clear language, and avoid arguing or challenging the person's perception of reality.
5. Attend to physical needs: Attend to the person's physical needs, such as providing water, food, or warmth, if necessary. Offer assistance with mobility, if needed, to prevent falls or injuries.
6. Provide familiar items: Offer familiar items, such as a favorite blanket, family photo, or familiar object, to provide comfort and familiarity to the person.
7. Be patient and understanding: Understand that individuals with dementia may have difficulty communicating, understanding instructions, or expressing their needs. Be patient, compassionate, and empathetic in your interactions.
8. Involve caregivers or family members: If the person has caregivers or family members, involve them in the first aid process, and keep them informed about the situation.
9. Document and report: Document the details of the incident, including the date, time, and any observed symptoms or changes in behavior. Report the incident to the person's healthcare provider, caregivers, or family members, as appropriate.

It's important to remember that providing first aid for individuals with dementia may require additional patience, understanding, and adaptability due to the unique challenges and behaviors associated with the condition. Consulting with healthcare professionals or dementia care specialists can provide further guidance on providing appropriate first aid for individuals with dementia or Alzheimer's disease.

- 1. What are the essential coping strategies for dementia?

Coping strategies for individuals with dementia can help manage the challenges and symptoms associated with the condition, improve their quality of life, and support their well-being. Here are some essential coping strategies for dementia:

1. Establish a routine: Creating a consistent daily routine can provide structure and familiarity for individuals with dementia. This can include regular times for meals, activities, and rest, which can help reduce confusion and anxiety.
2. Simplify the environment: Simplifying the environment can reduce sensory overload and confusion. This can include decluttering living spaces, using simple and familiar objects, providing clear signage, and minimizing noise and distractions.
3. Use clear and simple communication: Use simple and clear language when communicating with individuals with dementia. Avoid using complex or abstract concepts, and use visual cues, gestures, and facial expressions to support communication.
4. Foster a calm and supportive environment: Creating a calm and supportive environment can help reduce agitation and anxiety in individuals with dementia. This can include providing a calm and soothing atmosphere, avoiding confrontation or arguing, and providing reassurance and comfort.
5. Engage in meaningful activities: Encouraging engagement in meaningful activities can provide purpose, enjoyment, and cognitive stimulation for individuals with dementia. This can include activities such as hobbies, music, art, puzzles, and reminiscence therapy.
6. Provide opportunities for physical exercise: Regular physical exercise can have numerous benefits for individuals with dementia, including improved physical health, mood, and cognitive function. Activities such as walking, chair exercises, and gentle movements can be beneficial.
7. Practice self-care for caregivers: Caregivers of individuals with dementia also need to take care of themselves. This includes managing their own physical and emotional well-being, seeking support from other caregivers or professionals, and taking regular breaks to prevent caregiver burnout.
8. Seek professional support: Consulting with healthcare professionals, dementia care specialists, and support groups can provide valuable guidance, resources, and emotional support for both individuals with dementia and their caregivers.
9. Consider medication and other interventions: In some cases, medication or other interventions may be used to manage symptoms of dementia, such as agitation, depression, or sleep disturbances. Consult with healthcare professionals for appropriate medical management.

Each person with dementia is unique, and coping strategies may need to be tailored to their specific needs, abilities, and preferences. It's important to work closely with healthcare professionals, dementia care specialists, and caregivers to develop a personalized care plan that addresses the individual's unique challenges and promotes their well-being.

- 1. What are the 4 steps when addressing a patient with dementia?

When addressing a patient with dementia, it can be helpful to follow these four steps:

1. Approach with respect and empathy: Approach the person with dementia in a respectful and empathetic manner, using a calm and friendly tone of voice. Make eye contact and use non-verbal cues, such as a gentle touch, to establish a connection.
2. Introduce yourself and use their name: Introduce yourself and address the person by their preferred name, if known. This can help create a sense of familiarity and establish rapport.
3. Use simple and clear communication: Use simple and clear language when communicating, avoiding jargon or complex concepts. Speak slowly and give the person enough time to process and respond. Use visual cues, gestures, and facial expressions to support communication.
4. Listen actively and validate their feelings: Listen actively to what the person is saying, and validate their feelings and emotions. Avoid dismissing their concerns or correcting them if they are confused. Instead, respond with empathy and understanding.

These steps can help create a positive and supportive interaction with a person with dementia, reduce communication difficulties, and promote effective communication. It's important to approach each person with dementia as an individual and adapt your communication style based on their unique needs and abilities.

- 1. What is the medical management of dementia?

Medical management of dementia typically involves a multi-faceted approach that may include the following:

1. Medication: There are several medications available that may be used to manage symptoms of dementia, depending on the type and stage of dementia. These medications include cholinesterase inhibitors (such as donepezil, rivastigmine, and galantamine) and N-methyl-D-aspartate (NMDA) receptor antagonists (such as memantine). These medications are used to help manage cognitive symptoms, such as memory loss, thinking skills, and behavior.
2. Treating underlying health conditions: Addressing and managing underlying health conditions, such as hypertension, diabetes, depression, and sleep disorders, may help slow the progression of dementia or alleviate some of its symptoms. Medical interventions for these conditions may include medication, lifestyle changes, and other appropriate treatments.
3. Behavioral interventions: Non-pharmacological interventions, such as behavioral strategies, may also be used to manage symptoms of dementia. These may include strategies such as creating a structured routine, using memory aids, providing a safe and supportive environment, and managing challenging behaviors through behavior modification techniques.
4. Supportive care: Providing supportive care for individuals with dementia, including assistance with activities of daily living (ADLs), managing nutrition and hydration, and addressing safety concerns, is an important part of medical management. This may involve working with caregivers, healthcare professionals, and other support services to ensure the person's physical, emotional, and social well-being.
5. Caregiver education and support: Caregivers play a crucial role in the management of dementia. Educating caregivers about dementia, providing them with coping strategies, and offering emotional support can be beneficial in managing the condition and promoting the well-being of both the person with dementia and their caregivers.
6. Regular monitoring and follow-up: Regular monitoring and follow-up with healthcare professionals, including neurologists, geriatricians, psychiatrists, and other specialists, is important in the medical management of dementia. This may involve periodic assessments of cognitive function, review of medication effectiveness and side effects, and adjustment of care plans as needed.

It's important to note that there is no cure for most types of dementia, and medical management focuses on symptom management, improving quality of life, and supporting the well-being of individuals with dementia and their caregivers. The specific medical management approach will depend on the type and stage of dementia, as well as the individual needs and preferences of the person with dementia. Consulting with healthcare professionals experienced in dementia care is essential to develop an appropriate and personalized care plan.

**Emergency situations for patients with dementia:**

- 1. When is dementia an emergency?

Dementia itself is not typically considered an emergency. However, there are certain situations related to dementia that may require emergency medical attention. These may include:

1. Sudden and severe changes in behavior or mental status: If a person with dementia experiences a sudden and severe change in behavior or mental status, such as extreme agitation, aggression, confusion, delusions, or hallucinations, it may be necessary to seek emergency medical care. These changes may be indicative of an underlying medical condition or a potential safety concern, and prompt evaluation by a healthcare professional is important.
2. Physical injury or medical crisis: If a person with dementia sustains a physical injury, such as a fall, a burn, or a severe cut, or experiences a medical crisis, such as difficulty breathing, chest pain, severe headache, or stroke-like symptoms, it may require immediate medical attention. These situations may pose a risk to the person's health and safety, and emergency medical care may be needed.
3. Wandering or getting lost: If a person with dementia wanders away from home or gets lost, it can be a dangerous situation. Wandering is common in dementia and can pose risks such as getting lost, exposure to harsh weather conditions, falls, or other injuries. If a person with dementia is missing or has wandered, it is important to contact local authorities and initiate a search immediately.
4. Suicidal ideation or intent: If a person with dementia expresses suicidal thoughts, intentions, or engages in self-harming behavior, it is a serious concern that requires immediate attention. Contacting emergency services or a mental health professional is essential to ensure the person's safety.

In these situations, it is important to call emergency services or go to the nearest emergency department for prompt evaluation and appropriate medical care. It's also important to have a plan in place for emergencies related to dementia, such as having emergency contact numbers readily available and developing a safety plan with guidance from healthcare professionals. Caregivers and family members should be vigilant in monitoring the well-being of individuals with dementia and seeking appropriate medical attention when necessary.

- 1. What should a caregiver do to help a dementia client in an emergency?

As a caregiver of a dementia client, it's important to be prepared and have a plan in place for emergencies. Here are some steps you can take to help a dementia client in an emergency:

1. Stay calm: It's crucial to stay calm and composed in an emergency situation. This will help you make clear decisions and provide appropriate care to the person with dementia.
2. Assess the situation: Evaluate the nature and severity of the emergency. Is it a medical emergency, a safety concern, or a behavioral issue related to dementia? Assessing the situation will help you determine the appropriate course of action.
3. Call for help: If it's a medical emergency or a safety concern, call emergency services (such as 911 in the United States) for immediate assistance. Provide clear and concise information about the situation and the person's condition to the emergency operator.
4. Follow any established safety plans: If you have a safety plan in place for emergencies related to dementia, follow it. This may include instructions on how to handle wandering, falls, agitation, or other specific situations.
5. Ensure safety: Take steps to ensure the safety of the person with dementia and others around them. This may include removing potential hazards, providing first aid or basic medical care, and using calming techniques to manage behavioral symptoms.
6. Reassure and comfort: People with dementia may become confused, agitated, or scared during an emergency. Offer reassurance, comfort, and support to help calm their anxiety and confusion.
7. Communicate effectively: Use simple and clear communication with the person with dementia. Avoid using complex language, speaking too fast, or overwhelming them with too much information. Use non-verbal cues, such as touch or visual cues, to communicate effectively.
8. Stay with the person: It's important to stay with the person with dementia throughout the emergency and provide continuous reassurance and support. Avoid leaving them alone unless it is absolutely necessary for their safety.
9. Document the incident: Take note of the details of the emergency, including the time, location, and nature of the event. This information may be helpful for healthcare professionals and caregivers in future care planning.
10. Follow up: After the emergency has been resolved, follow up with appropriate medical care or further actions as needed. Update healthcare professionals and other involved parties about the situation and any changes in the person's condition.

Remember, emergencies can be stressful and challenging, but having a plan in place, staying calm, and seeking appropriate help can help ensure the safety and well-being of the person with dementia. It's important to work closely with healthcare professionals, have emergency contact numbers readily available, and regularly review and update the emergency plan as needed.

Top of Form

- 1. What safety precautions should dementia patients take in the hospital?

When caring for dementia patients in a hospital setting, it's important to take specific safety precautions to ensure their well-being. Here are some general safety precautions that can be implemented:

1. Identification: Make sure the person with dementia has proper identification, such as a hospital wristband or name tag, to prevent confusion and aid in identification by hospital staff.
2. Fall prevention: Dementia patients may have an increased risk of falls due to their cognitive and physical limitations. Implement fall prevention measures, such as bed alarms, non-slip footwear, and assistive devices (e.g., bed rails, walking aids) to minimize fall risk.
3. Wandering prevention: Dementia patients may wander and become disoriented in an unfamiliar hospital environment. Implement measures to prevent wandering, such as using door alarms, providing clear signage, and ensuring supervised areas.
4. Medication management: Dementia patients may have difficulty managing their medications independently. Ensure that medications are administered according to the prescribed schedule and that they are stored securely to prevent accidental ingestion or overdose.
5. Agitation and behavioral management: Dementia patients may exhibit agitation, confusion, or other challenging behaviors in the hospital setting. Implement strategies for managing agitation, such as providing a calm and structured environment, using familiar objects or routines, and providing appropriate social interaction and reassurance.
6. Communication: Use simple and clear communication techniques when interacting with dementia patients in the hospital. Speak slowly, use familiar language, and provide visual cues as needed to help improve understanding and reduce anxiety.
7. Nutrition and hydration: Ensure that dementia patients are receiving adequate nutrition and hydration during their hospital stay. Monitor their intake and offer food and fluids at regular intervals, taking into consideration any dietary restrictions or preferences.
8. Infection control: Implement infection control measures, including proper hand hygiene, use of personal protective equipment, and regular cleaning and disinfection of the hospital environment, to prevent the spread of infections.
9. Supportive care: Provide appropriate emotional support and comfort to dementia patients in the hospital. This may include offering reassurance, providing familiar objects or photos, and involving family members or caregivers in care decisions.
10. Family involvement: Involve family members or caregivers in the care of dementia patients in the hospital. They can provide valuable information about the person's preferences, behaviors, and medical history, and can also offer emotional support and assistance in managing the person's care.

It's important to work closely with the healthcare team, including nurses, physicians, and other healthcare professionals, to ensure that appropriate safety precautions are in place for dementia patients in the hospital setting. Individualized care plans based on the specific needs and behaviors of each person with dementia should be developed and implemented to provide safe and quality care.

- 1. What do you do when a dementia patient is out of control?

Dealing with a dementia patient who is out of control can be challenging, and it's important to prioritize the safety and well-being of the person with dementia, as well as those around them. Here are some steps to take when a dementia patient is exhibiting challenging behaviors or is out of control:

1. Stay calm: It's important to remain calm and composed in a difficult situation. Avoid raising your voice, using aggressive or confrontational language, or responding with frustration, as this may escalate the situation further.
2. Assess for underlying causes: Try to identify any underlying causes that may be contributing to the challenging behavior. It could be pain, discomfort, hunger, thirst, fear, or other triggers. Addressing the underlying cause may help resolve or manage the behavior.
3. Provide reassurance: Offer reassurance to the person with dementia in a calm and comforting manner. Use a calm and soothing tone of voice, and offer words of reassurance and comfort to help the person feel safe and secure.
4. Redirect or distract: Redirect the person's attention to a different activity or topic to help shift their focus away from the challenging behavior. Distraction techniques, such as offering a favorite object, engaging in a familiar activity, or talking about a pleasant memory, may help redirect their behavior.
5. Use validation and empathy: Use validation and empathy to acknowledge and validate the person's feelings and emotions, even if they may not be grounded in reality. Avoid arguing, correcting, or dismissing their concerns, as this may escalate the situation further.
6. Remove triggers: If possible, remove any triggers or environmental factors that may be contributing to the challenging behavior. For example, if the person with dementia is agitated due to loud noises, try to reduce the noise level in the environment.
7. Seek help from healthcare professionals: If the situation is escalating or if the person with dementia poses a risk to themselves or others, seek help from healthcare professionals, such as a nurse, physician, or other trained personnel. They can provide guidance, support, and intervention to manage the situation safely.
8. Ensure safety: Prioritize safety at all times. If the person with dementia is in danger of harming themselves or others, take appropriate measures to ensure their safety. This may involve calling for assistance, moving away from potential hazards, or providing physical assistance to prevent injury.
9. Involve caregivers or family members: If the person with dementia has caregivers or family members, involve them in the situation and seek their support. They may have valuable insights into the person's behaviors and preferences, and can help provide comfort and reassurance.
10. Follow care plan: If the person with dementia has a care plan or behavior management plan in place, follow it as closely as possible. This may include specific strategies or interventions designed to manage challenging behaviors.

Dealing with an out-of-control dementia patient can be challenging, and it's important to prioritize safety, remain calm, and seek appropriate help when needed. Working closely with healthcare professionals, caregivers, and family members can help ensure the well-being of the person with dementia and those around them.

**Conflict resolution for patients with dementia:**

1- How do you handle conflict with dementia patients?

2- What strategies can you use to help a person with dementia cope?

3- How do you calm a combative dementia patient?

4- How do you redirect an angry dementia patient?

**Patient Ethics for Patients with Dementia:**

- 1. What are the ethics of patient dementia?

Ethics in the context of dementia care encompass a range of principles and considerations that guide healthcare professionals, caregivers, and others involved in the care of individuals with dementia. Some key ethical considerations in dementia care include:

1. Autonomy and decision-making: Individuals with dementia may experience cognitive decline and may have difficulties making decisions or expressing their preferences. It is important to respect their autonomy and support their decision-making capacity to the extent possible, while also ensuring their safety and well-being. This may involve using strategies such as shared decision-making, providing information in a way that is understandable to the person with dementia, and involving caregivers or family members in decision-making processes when appropriate.
2. Beneficence and non-maleficence: Healthcare professionals and caregivers have a duty to act in the best interests of the person with dementia, promoting their well-being and avoiding harm. This may involve providing appropriate care and support, managing symptoms and behaviors effectively, addressing pain and discomfort, and considering the person's physical, emotional, and social needs.
3. Dignity and respect: People with dementia should be treated with dignity and respect, regardless of their cognitive abilities. This includes maintaining their privacy, providing person-centered care that considers their individual preferences and values, and avoiding practices that may demean or stigmatize them.
4. Truth-telling and honesty: Healthcare professionals and caregivers should strive to be truthful and honest with individuals with dementia to the extent possible, while taking into consideration their cognitive abilities, emotional state, and the potential impact of the information on their well-being. This may involve providing information in a gentle and compassionate manner, using validation techniques, and considering the person's capacity to understand and process information.
5. Justice and fairness: Ensuring equitable access to care and resources for individuals with dementia is an important ethical consideration. This may involve addressing issues related to healthcare disparities, advocating for the rights and needs of individuals with dementia, and promoting social justice in the provision of dementia care services.
6. End-of-life care and advanced care planning: Ethical considerations related to end-of-life care and advanced care planning are important in dementia care. This includes respecting the person's wishes regarding end-of-life care, discussing and documenting their preferences for care in advance, and involving caregivers and family members in decision-making processes.
7. Confidentiality and privacy: Protecting the confidentiality and privacy of individuals with dementia is important, while also ensuring that necessary information is shared with appropriate healthcare professionals, caregivers, and family members to provide safe and effective care. This may involve adhering to relevant privacy laws and regulations, obtaining informed consent for sharing information, and maintaining appropriate documentation practices.
8. Cultural and social considerations: Consideration of cultural, social, and diversity-related factors is important in dementia care. This includes respecting the person's cultural beliefs, practices, and values, as well as understanding the social and environmental context in which they live, and tailoring care approaches accordingly.
9. Caregiver support: Ethical considerations in dementia care also extend to caregivers, who may face significant challenges and burdens in providing care for individuals with dementia. This includes providing support, education, and resources to caregivers to promote their well-being and ensure they are able to provide appropriate care.

Ethical considerations in dementia care are complex and multifaceted, and it is important for healthcare professionals, caregivers, and others involved in dementia care to carefully consider and apply ethical principles in their practice to ensure the best possible care and support for individuals with dementia.

- 1. How cultural and ethical issues impact dementia?

Cultural and ethical issues can have a significant impact on how dementia is perceived, diagnosed, and managed in different populations and communities. Some ways in which cultural and ethical issues can impact dementia care include:

1. Perceptions of dementia: Cultural beliefs and attitudes towards dementia may influence how it is perceived and understood within a particular community. For example, some cultures may view dementia as a normal part of aging or attribute it to supernatural causes, while others may view it as a taboo or shameful condition. These perceptions can impact help-seeking behaviors, attitudes towards diagnosis and treatment, and acceptance of dementia care interventions.
2. Diagnosis and assessment: Cultural factors can impact the process of diagnosing dementia, including language barriers, differences in cognitive and communication styles, and varying cultural norms for expressing cognitive complaints. Some cultural groups may be less likely to seek medical help for cognitive symptoms due to cultural beliefs or stigma, which can lead to delayed or missed diagnoses. Additionally, cultural norms and values may impact the acceptability and accuracy of commonly used cognitive assessment tools, requiring adaptation or modification for cultural appropriateness.
3. Treatment and care preferences: Cultural beliefs, values, and traditions may impact the preferences and expectations of individuals with dementia and their caregivers regarding treatment and care. For example, some cultural groups may prioritize traditional or alternative therapies over Western medical approaches, or may have specific preferences for home-based care or religious/spiritual interventions. These cultural preferences may need to be taken into consideration in developing a care plan that aligns with the individual's and family's values and beliefs.
4. Decision-making and autonomy: Cultural norms related to decision-making and autonomy can impact the ability of individuals with dementia to make decisions about their own care, as well as the involvement of family members or caregivers in decision-making processes. In some cultures, family members may play a prominent role in decision-making for individuals with dementia, which may impact the autonomy and agency of the person with dementia. It is important to respect cultural norms while also promoting the autonomy and decision-making capacity of the individual to the extent possible.
5. End-of-life care: Cultural beliefs and practices related to end-of-life care and death may impact the care and decision-making for individuals with dementia at the end of life. For example, cultural beliefs about afterlife, funeral rites, and mourning practices may influence decisions about palliative care, advanced care planning, and end-of-life interventions. Cultural considerations should be taken into account when discussing end-of-life care preferences and developing care plans.
6. Caregiver burden and support: Cultural factors can impact the experiences and needs of caregivers of individuals with dementia. Cultural norms and expectations may shape the roles and responsibilities of caregivers, as well as their attitudes towards seeking help or using formal care services. Cultural considerations should be taken into account in providing support, education, and resources to caregivers to ensure that interventions are culturally appropriate and effective.

It is important for healthcare professionals, caregivers, and others involved in dementia care to be aware of and sensitive to cultural and ethical issues, and to consider these factors in providing person-centered, culturally appropriate care that respects the values, beliefs, and preferences of individuals with dementia and their families.

- 1. What are the ethical issues of Alzheimer's disease?

There are several ethical issues related to Alzheimer's disease, which is a type of dementia. Some of the key ethical issues associated with Alzheimer's disease include:

1. Informed consent: Individuals with Alzheimer's disease may have diminished capacity to understand and provide informed consent for medical treatments, research participation, or other interventions. Ethical considerations arise in determining the appropriate level of decision-making capacity, ensuring that individuals are fully informed to the extent possible, and obtaining informed consent from individuals with Alzheimer's disease for their medical care and participation in research.
2. Autonomy and personhood: Individuals with Alzheimer's disease may experience a decline in their ability to make decisions, communicate, and express their preferences. Ethical considerations arise in balancing the autonomy and personhood of individuals with Alzheimer's disease with the need to ensure their safety, well-being, and quality of care. Ethical principles such as respect for autonomy, beneficence, and non-maleficence need to be carefully considered in the care of individuals with Alzheimer's disease.
3. End-of-life care: Ethical issues arise in decision-making about end-of-life care for individuals with Alzheimer's disease. This includes decisions about the use of life-sustaining treatments, palliative care, and advanced care planning. Ethical considerations include respecting the wishes and values of the individual with Alzheimer's disease, balancing the benefits and burdens of interventions, and ensuring that end-of-life care is aligned with the individual's preferences and values.
4. Caregiver burden and support: Alzheimer's disease can place significant burdens on caregivers, including physical, emotional, and financial burdens. Ethical considerations arise in ensuring that caregivers are supported, informed, and able to provide adequate care while maintaining their own well-being. This includes issues related to caregiver rights, responsibilities, and access to support services.
5. Equity and access to care: Ethical considerations arise in ensuring that individuals with Alzheimer's disease have equitable access to appropriate care, including diagnosis, treatment, and support services. This includes issues related to disparities in access to care based on factors such as race, ethnicity, socioeconomic status, geographic location, and other social determinants of health.
6. Research and clinical trials: Ethical considerations arise in conducting research and clinical trials involving individuals with Alzheimer's disease. This includes issues related to informed consent, recruitment, enrollment, and ethical considerations in research design, conduct, and dissemination of results. Ethical principles such as beneficence, respect for autonomy, and justice need to be carefully considered in research involving individuals with Alzheimer's disease.
7. Privacy and confidentiality: Ethical considerations arise in protecting the privacy and confidentiality of individuals with Alzheimer's disease, including issues related to the use of personal health information, disclosure of diagnosis, and sharing of information with family members, caregivers, and other healthcare providers.

It is important for healthcare professionals, caregivers, researchers, and policymakers to be aware of and address these ethical issues in the care of individuals with Alzheimer's disease, with a focus on promoting person-centered care, respecting autonomy and dignity, ensuring access to care, and upholding ethical principles in research and clinical practice.

- 1. What are ethical considerations when treating patients?

Ethical considerations in treating patients encompass a broad range of principles and values that guide healthcare professionals in providing optimal care while upholding ethical standards. Some of the key ethical considerations when treating patients include:

1. Respect for autonomy: This principle emphasizes the right of patients to make their own decisions about their own healthcare, even if those decisions are different from what the healthcare professional might choose. It involves obtaining informed consent, respecting the patient's values, preferences, and choices, and involving the patient in decision-making to the extent possible.
2. Beneficence: This principle involves the obligation of healthcare professionals to act in the best interest of the patient, providing care that promotes the patient's well-being and health. It requires healthcare professionals to seek the best possible outcomes for patients while balancing potential benefits and risks.
3. Non-maleficence: This principle, often summarized as "do no harm," involves the obligation of healthcare professionals to avoid causing harm to patients and to minimize potential risks and harms associated with treatment or interventions. It requires careful assessment of risks and benefits, and consideration of potential harm in decision-making.
4. Justice: This principle involves the fair distribution of healthcare resources and services, and the equitable treatment of patients regardless of factors such as race, ethnicity, socioeconomic status, age, or other social determinants of health. It emphasizes the need to provide healthcare services in a fair, just, and unbiased manner, without discrimination or bias.
5. Confidentiality and privacy: These principles involve protecting the privacy and confidentiality of patients' personal health information, and respecting their right to privacy in their healthcare interactions. Healthcare professionals have an obligation to maintain patient confidentiality and privacy, unless there are compelling reasons to breach it, such as when required by law or when necessary to protect the patient or others from harm.
6. Honesty and transparency: These principles involve being truthful and transparent with patients about their diagnosis, prognosis, treatment options, and other relevant information. It involves providing patients with accurate and complete information to enable them to make informed decisions about their healthcare.
7. Cultural and diversity considerations: These principles involve being sensitive to the cultural, religious, and diversity-related needs of patients, and providing care that is respectful of their beliefs, values, and practices. It requires understanding and accommodating patients' cultural backgrounds, language preferences, and other unique needs in the provision of care.
8. Collaboration and teamwork: These principles involve collaborating with other healthcare professionals, patients, and their families in a team-based approach to care. It involves effective communication, shared decision-making, and interdisciplinary collaboration to ensure comprehensive and coordinated care for patients.

Ethical considerations are an integral part of healthcare practice and play a crucial role in ensuring that patients receive safe, effective, and patient-centered care. Healthcare professionals should be familiar with ethical principles and guidelines, and strive to uphold them in their clinical practice, while considering the unique circumstances and needs of each patient.

**Helpful experiences of other caregivers:**

- 1. What are the experiences of carers of people with dementia?

The experiences of caregivers of people with dementia can vary widely depending on various factors such as the stage of dementia, the severity of symptoms, the caregiver's relationship with the person with dementia, the availability of support and resources, and the caregiver's own physical and emotional well-being. However, some common experiences reported by caregivers of people with dementia may include:

1. Emotional and physical strain: Caring for a person with dementia can be emotionally and physically demanding. Witnessing the decline of a loved one's cognitive and functional abilities can be distressing and emotionally challenging. Caregivers may also experience physical strain due to the demands of caregiving tasks, such as assisting with mobility, personal care, and managing challenging behaviors.
2. Role changes and impact on personal life: Caregiving for a person with dementia often requires significant adjustments to the caregiver's daily routine, roles, and responsibilities. Caregivers may need to dedicate considerable time and effort to provide care, which may impact their own personal and professional life. They may have to give up or reduce their work hours, social activities, and personal hobbies, and experience changes in their relationships with friends and other family members.
3. Financial burden: Caring for a person with dementia can also result in financial strain, as costs associated with medical care, medications, home modifications, and other caregiving-related expenses can add up over time. Caregivers may face financial challenges in providing optimal care for their loved one with dementia, and may need to navigate complex healthcare and financial systems to access necessary resources and support.
4. Emotional distress and mental health challenges: Caregivers of people with dementia may experience a wide range of emotions, including sadness, frustration, guilt, anxiety, and depression. The chronic stress and emotional burden of caregiving can take a toll on caregivers' mental health and well-being, and may require them to seek support from mental health professionals or other caregivers.
5. Social isolation and decreased quality of life: Caregiving responsibilities can limit caregivers' opportunities for social interaction, leisure activities, and self-care. Caregivers may experience social isolation and decreased quality of life as they devote significant time and energy to caregiving, which can affect their own physical and mental well-being.
6. Uncertainty and grief: Caring for a person with dementia involves coping with the uncertainty of the disease progression and the changing needs of the person with dementia. Caregivers may experience grief and loss as they witness the decline of their loved one's cognitive and functional abilities, and may need support to navigate through the emotional challenges associated with dementia care.
7. Resilience and personal growth: Despite the challenges, many caregivers also report positive experiences, such as increased compassion, resilience, and personal growth. Caregiving for a person with dementia can provide an opportunity for caregivers to develop new skills, deepen relationships, and find meaning and purpose in their caregiving role.

It's important to note that caregiving experiences can be highly individual and can vary widely depending on the unique circumstances of each caregiver and person with dementia. Caregivers should seek support from healthcare professionals, support groups, and other caregivers to cope with the challenges and complexities of caregiving, and prioritize their own physical and emotional well-being while caring for their loved one with dementia.

Top of Form

- 1. How can we support the caregivers of dementia patients?

Supporting caregivers of dementia patients is crucial as they play a critical role in providing care and improving the quality of life for individuals with dementia. Here are some ways to support caregivers of dementia patients:

1. Education and Information: Provide caregivers with education and information about dementia, its symptoms, progression, and management strategies. This can help caregivers better understand the condition and develop effective caregiving strategies.
2. Caregiver Training: Offer caregiver training programs that provide practical skills and techniques for managing dementia-related symptoms, including communication strategies, behavioral management techniques, and safety precautions.
3. Respite Care: Provide respite care options to allow caregivers to take breaks and have time for self-care. Respite care can be in the form of professional in-home care services, adult day care programs, or temporary residential care.
4. Support Groups: Offer support groups for caregivers of dementia patients to provide emotional support, share experiences, and exchange information with others who are facing similar challenges. Support groups can help reduce caregiver isolation and provide a sense of community.
5. Counseling and Mental Health Support: Provide access to counseling services or mental health support to help caregivers manage stress, anxiety, depression, and other mental health challenges associated with caregiving.
6. Access to Resources: Connect caregivers with available resources and services in the community, such as financial assistance, legal support, and support from local Alzheimer's or dementia organizations.
7. Flexible Work Arrangements: Support caregivers in their workplace by offering flexible work arrangements, such as adjusted work hours, telecommuting, or leave of absence, to accommodate their caregiving responsibilities.
8. Caregiver Self-Care: Encourage and promote caregiver self-care, including proper nutrition, exercise, adequate sleep, and regular breaks from caregiving responsibilities to prevent burnout and promote well-being.
9. Respecting Caregiver Choices: Recognize and respect the autonomy and choices of caregivers, including their decisions regarding the care and treatment of the person with dementia, while providing guidance and support as needed.
10. Collaboration with Healthcare Professionals: Foster collaboration between caregivers and healthcare professionals, including regular communication, shared decision-making, and coordinated care to ensure the best possible care for the person with dementia and support for the caregiver.

Supporting caregivers of dementia patients is crucial to help them manage the challenges associated with caregiving and ensure the well-being of both the caregiver and the person with dementia. It's important to assess the unique needs of each caregiver and provide tailored support accordingly.

3-How do you support carers and others to support an individual with dementia in the final stages of their life?

Supporting caregivers and others to support an individual with dementia in the final stages of their life requires a compassionate and holistic approach. Here are some key considerations:

1. Emotional Support: Offer emotional support to caregivers and others involved in the care of the person with dementia. This may include providing empathetic listening, validation of their feelings and experiences, and offering comfort and reassurance.
2. Education and Information: Provide caregivers and others with education and information about the expected progression of dementia in the final stages, including common symptoms, changes in care needs, and end-of-life care options. This can help them better understand what to expect and how to provide appropriate care.
3. Palliative Care and Comfort Measures: Collaborate with palliative care professionals to develop a comprehensive care plan that focuses on comfort and quality of life for the person with dementia in the final stages. This may include pain management, symptom control, and other comfort measures to ensure the person's physical and emotional well-being.
4. Advance Care Planning: Facilitate discussions and decision-making regarding advance care planning, including the person's wishes for end-of-life care, preferences for medical interventions, and other considerations. This can help ensure that the person's preferences and values are respected in their final stages of life.
5. Communication and Collaboration: Foster open communication and collaboration among caregivers, family members, healthcare professionals, and other relevant stakeholders involved in the care of the person with dementia. This can help ensure that everyone is on the same page and working together to provide the best possible care and support.
6. Respecting Cultural and Spiritual Beliefs: Respect and honor the cultural and spiritual beliefs of the person with dementia and their family, and incorporate these beliefs into the care plan as appropriate. This may include respecting specific rituals, customs, or religious practices that hold significance for the person and their family.
7. Respite and Support for Caregivers: Offer respite care and support to caregivers to prevent caregiver burnout and promote their well-being. This may include providing opportunities for caregivers to take breaks, providing emotional support, and connecting them with relevant resources and services.
8. Grief and Bereavement Support: Offer grief and bereavement support to caregivers and others involved in the care of the person with dementia after their passing. This may include providing emotional support, facilitating access to grief counseling or support groups, and offering resources to help cope with the loss.

Supporting caregivers and others to support an individual with dementia in the final stages of their life requires a compassionate and person-centered approach that focuses on physical, emotional, spiritual, and cultural well-being. Collaborating with healthcare professionals, providing education and information, and offering emotional support are essential components of effective care in the final stages of dementia.

- 1. What is the role of carers and others in supporting individuals with dementia?

Carers and others play a crucial role in supporting individuals with dementia in various ways. Here are some key roles they play:

1. Providing Care and Assistance: Carers and others provide direct care and assistance to individuals with dementia in their daily activities, including personal care, mobility support, meal preparation, medication management, and other essential tasks. They help ensure that the person with dementia is safe, comfortable, and well-cared for.
2. Emotional Support: Carers and others offer emotional support to individuals with dementia by providing companionship, engaging in meaningful activities, and offering empathy, reassurance, and validation. Emotional support is essential for promoting the person's emotional well-being and maintaining their quality of life.
3. Advocacy: Carers and others act as advocates for individuals with dementia, ensuring that their needs, preferences, and rights are respected and addressed. This may involve advocating for appropriate healthcare, social services, and legal matters, as well as navigating complex healthcare and caregiving systems on behalf of the person with dementia.
4. Communication and Social Interaction: Carers and others facilitate communication and social interaction for individuals with dementia, as communication difficulties are common in dementia. They use strategies such as validation, active listening, and non-verbal communication to enhance communication and foster social engagement, which can help reduce distress and improve the person's well-being.
5. Managing Challenging Behaviors: Carers and others often deal with challenging behaviors that may arise in individuals with dementia, such as agitation, aggression, wandering, and sundowning. They use strategies such as redirection, distraction, and calming techniques to manage these behaviors in a compassionate and person-centered manner.
6. Coordination of Care: Carers and others play a critical role in coordinating and managing the overall care of individuals with dementia, including coordinating healthcare appointments, managing medications, arranging for home care services, and collaborating with healthcare professionals and other caregivers to ensure continuity and quality of care.
7. Education and Information: Carers and others seek education and information about dementia to better understand the condition and how to provide appropriate care. They may attend educational programs, read literature, and consult with healthcare professionals to learn about dementia, its symptoms, progression, and how to provide effective care and support.
8. Self-Care: Carers and others also need to prioritize self-care to maintain their own physical, emotional, and mental well-being. This may involve taking breaks, seeking support from others, engaging in activities they enjoy, and accessing respite care services to prevent caregiver burnout and maintain their ability to provide effective care.

Overall, the role of carers and others in supporting individuals with dementia is multi-faceted and essential for promoting the well-being and quality of life of individuals with dementia. It involves providing care and assistance, emotional support, advocacy, communication facilitation, managing challenging behaviors, coordinating care, seeking education and information, and prioritizing self-care. Collaborating with healthcare professionals, accessing support services, and practicing self-care are critical components of effective caregiving for individuals with dementia.

**How to deal with family and friends of patients with dementia**

- 1. How can family and friends help someone with dementia?

Family and friends can play a crucial role in helping someone with dementia in various ways. Here are some ways family and friends can provide support:

1. Emotional Support: Offer emotional support to the person with dementia by being patient, empathetic, and understanding. Show compassion and listen actively to their concerns and feelings without judgment. Offer reassurance, validation, and comfort to help them manage their emotions and maintain their well-being.
2. Maintain Familiarity: Help maintain a familiar environment and routine for the person with dementia. Avoid making significant changes to their living arrangements or daily routines, as familiarity and routine can help reduce confusion and anxiety for individuals with dementia.
3. Promote Independence: Support the person with dementia in maintaining their independence and dignity for as long as possible. Encourage them to participate in activities they enjoy and can manage independently, such as self-care, hobbies, and social interactions, to promote their self-esteem and well-being.
4. Communication and Social Interaction: Practice effective communication strategies when interacting with the person with dementia. Use simple and clear language, speak slowly and calmly, and avoid talking down to them. Be patient and give them time to process information and respond. Engage in meaningful conversations and activities to promote social interaction and engagement.
5. Offer Practical Assistance: Provide practical assistance with daily activities such as meal preparation, medication management, transportation, and household tasks. This can help ease the burden on the person with dementia and ensure their safety and well-being.
6. Advocate for Appropriate Care: Advocate for appropriate healthcare and social services for the person with dementia. Collaborate with healthcare professionals and other caregivers to ensure the person with dementia receives appropriate medical care, support services, and legal protection.
7. Educate Yourself: Seek education and information about dementia to better understand the condition and how to provide effective care. Learn about the symptoms, progression, and management of dementia to provide informed support to the person with dementia.
8. Take Care of Yourself: Caring for someone with dementia can be physically and emotionally challenging. It's important for family and friends to prioritize their own physical, emotional, and mental well-being. Practice self-care, seek support from others, and consider accessing respite care services to prevent caregiver burnout.
9. Engage in Supportive Activities: Participate in supportive activities and programs for caregivers of individuals with dementia, such as support groups, counseling, and respite care services. These resources can provide emotional support, information, and practical assistance for caregivers.
10. Respect the Person's Dignity and Autonomy: Respect the person's dignity and autonomy, even as their cognitive abilities decline. Avoid talking down to them, making decisions for them without their input, or treating them as helpless. Encourage their participation in decision-making and respect their choices to the extent possible.

Support from family and friends can make a significant positive impact on the well-being and quality of life of individuals with dementia. It involves providing emotional support, maintaining familiarity, promoting independence, effective communication, practical assistance, advocacy for appropriate care, education, self-care, engaging in supportive activities, and respecting the person's dignity and autonomy. Collaborating with healthcare professionals, accessing support services, and practicing self-care are critical components of providing effective support to someone with dementia.

Top of Form

- 1. How do you deal with a friend with dementia?

Dealing with a friend who has dementia can be challenging, but it's important to approach the situation with compassion, understanding, and patience. Here are some tips on how to deal with a friend with dementia:

1. Educate Yourself: Educate yourself about dementia and its symptoms, progression, and management. Understanding the condition can help you better comprehend what your friend is going through and how you can support them.
2. Be Patient and Understanding: Dementia can cause changes in behavior, memory loss, confusion, and other cognitive difficulties. Be patient and understanding when your friend exhibits these symptoms. Avoid getting frustrated or angry, as it may exacerbate the situation.
3. Communicate Clearly: Use simple, clear language when communicating with your friend. Avoid using jargon, complex sentences, or ambiguous questions. Speak slowly and calmly, and give them time to process information and respond. Use non-verbal cues and gestures to assist with communication.
4. Maintain Familiarity: Help maintain a familiar environment and routine for your friend. Avoid making significant changes to their living arrangements or daily routines, as familiarity can help reduce confusion and anxiety.
5. Encourage Independence: Support your friend in maintaining their independence and dignity for as long as possible. Encourage them to participate in activities they enjoy and can manage independently, and avoid doing things for them that they can still do on their own.
6. Offer Emotional Support: Be a source of emotional support for your friend. Listen actively to their concerns and feelings without judgment. Offer reassurance, validation, and comfort to help them manage their emotions and maintain their well-being.
7. Engage in Meaningful Activities: Engage in meaningful activities with your friend, such as reminiscing, engaging in hobbies, or doing activities they enjoy. Meaningful activities can help stimulate their cognitive function, provide a sense of purpose, and promote social interaction.
8. Be Flexible and Adaptive: Be flexible and adaptive in your approach to dealing with your friend's changing abilities and needs. Be prepared for their condition to evolve over time and adjust your expectations and approach accordingly.
9. Respect Their Dignity and Autonomy: Respect your friend's dignity and autonomy, even as their cognitive abilities decline. Avoid talking down to them or treating them as helpless. Encourage their participation in decision-making and respect their choices to the extent possible.
10. Seek Support for Yourself: Caring for a friend with dementia can be emotionally challenging. Seek support for yourself from other friends, family members, or support groups. Taking care of your own well-being will help you be better able to support your friend.

Dealing with a friend with dementia requires patience, understanding, and empathy. It's important to communicate clearly, maintain familiarity, encourage independence, offer emotional support, engage in meaningful activities, be flexible and adaptive, respect their dignity and autonomy, and seek support for yourself. Collaborating with healthcare professionals and other caregivers can also be beneficial in providing comprehensive care for your friend with dementia.

- 1. How do you deal with family conflict and dementia?

Dealing with family conflict when a loved one has dementia can be challenging, as the emotional and practical stressors of caregiving can sometimes lead to disagreements, misunderstandings, and conflicts among family members. Here are some tips for managing family conflict related to dementia:

1. Open and Honest Communication: Encourage open and honest communication among family members. Create a safe space where everyone can express their concerns, emotions, and opinions without judgment. Avoid assigning blame and focus on finding solutions together.
2. Educate Yourself and Others: Educate yourself and other family members about dementia, its symptoms, progression, and management. Understanding the condition can help family members better comprehend what their loved one is going through and how to provide appropriate care and support.
3. Develop a Care Plan: Develop a care plan in collaboration with all family members involved in the caregiving process. Clearly define roles, responsibilities, and expectations, and discuss and agree upon important decisions, such as medical care, living arrangements, and financial management.
4. Seek Mediation or Professional Help: If family conflicts become persistent and unmanageable, consider seeking the help of a neutral third party, such as a professional mediator or counselor. They can facilitate communication, provide guidance, and help resolve conflicts in a fair and neutral manner.
5. Practice Empathy and Compassion: Dementia can be emotionally challenging for both the person with dementia and their family members. Practice empathy and compassion towards each other's feelings and experiences. Recognize that everyone may be coping with the situation in their own way.
6. Focus on the Best Interests of the Person with Dementia: Keep the best interests of the person with dementia at the center of decision-making. Focus on their physical and emotional well-being, safety, and quality of life. This can help guide family discussions and decisions.
7. Take Care of Yourself: Caring for a loved one with dementia can be physically and emotionally draining. It's important to prioritize self-care and take breaks when needed to prevent burnout. Seek support from other family members, friends, or professionals, and practice self-compassion.
8. Be Flexible and Adaptive: Be willing to adapt and adjust the care plan as the needs of the person with dementia change over time. Dementia is a progressive condition, and caregiving may require ongoing adjustments to accommodate the changing abilities and needs of the person with dementia.

Dealing with family conflict related to dementia requires effective communication, empathy, collaboration, and a focus on the best interests of the person with dementia. Seeking professional help and practicing self-care can also be important in managing and resolving conflicts among family members.

**How to advocate for patients with dementia:**

- 1. What is the role of advocacy in dementia patients?

Advocacy plays a crucial role in supporting individuals with dementia and their families. Here are some key aspects of advocacy for dementia patients:

1. Raising Awareness: Advocacy efforts can help raise awareness about dementia as a condition, its impact on individuals and families, and the need for support and resources. This can help reduce stigma, increase understanding, and promote empathy and compassion towards people with dementia.
2. Promoting Access to Care and Support: Advocates can work to ensure that individuals with dementia have access to appropriate healthcare, social services, and support programs. This may include advocating for policy changes, funding for dementia care and research, and improved access to specialized dementia care services.
3. Empowering Patients and Families: Advocacy can empower individuals with dementia and their families by providing information, resources, and tools to navigate the challenges of the condition. This may include providing education, support groups, legal and financial guidance, and empowering individuals with dementia to have a voice in their care and decision-making process.
4. Influencing Policy and Legislation: Advocacy efforts can aim to influence policy and legislation related to dementia care, including advocating for improved standards of care, better training for healthcare professionals, and policies that protect the rights and dignity of individuals with dementia.
5. Enhancing Caregiver Support: Advocacy can focus on providing support for caregivers of individuals with dementia, who often experience significant physical, emotional, and financial burdens. This may include advocating for caregiver support programs, respite care, and financial assistance to alleviate the challenges faced by caregivers.
6. Promoting Research and Innovation: Advocacy efforts can support and promote research into the causes, prevention, and treatment of dementia. This may involve advocating for increased funding for dementia research, supporting clinical trials, and promoting innovations in care and interventions for individuals with dementia.
7. Influencing Dementia-Friendly Communities: Advocacy can work towards creating dementia-friendly communities that are inclusive, supportive, and accommodating to the needs of individuals with dementia. This may involve advocating for community engagement, accessibility, and awareness initiatives that promote inclusivity and understanding towards individuals with dementia.

Advocacy can play a critical role in improving the lives of individuals with dementia and their families by promoting awareness, access to care and support, empowerment, policy changes, caregiver support, research, and dementia-friendly communities. Advocacy efforts can help create a more supportive and inclusive environment for individuals with dementia to live with dignity and quality of life.

- 1. How do we help dementia patients?

Helping individuals with dementia requires a multifaceted approach that addresses their physical, emotional, social, and cognitive needs. Here are some general ways to help dementia patients:

1. Establishing a safe and supportive environment: Creating a safe and dementia-friendly environment can help prevent accidents, promote independence, and reduce confusion. This may involve modifying the home or care facility to remove hazards, ensuring proper lighting, using signs and labels for orientation, and providing familiar and meaningful objects.
2. Providing person-centered care: Understanding the unique needs, preferences, and abilities of individuals with dementia is crucial. Providing person-centered care involves tailoring care plans to the individual's needs, interests, and abilities, promoting autonomy, and respecting their dignity and choices.
3. Communicating effectively: Communication can be challenging for individuals with dementia due to cognitive impairments. Using simple and clear language, speaking slowly and calmly, using visual aids, and providing ample time for responses can help improve communication and reduce frustration.
4. Managing behavioral and psychological symptoms: Dementia patients may experience behavioral and psychological symptoms such as agitation, aggression, and wandering. Managing these symptoms may involve identifying and addressing triggers, providing a calm and structured environment, using diversional activities, and collaborating with healthcare professionals for appropriate interventions.
5. Promoting physical health: Maintaining physical health is important for individuals with dementia. Encouraging regular physical exercise, providing a nutritious diet, managing chronic health conditions, and promoting good sleep hygiene can help support overall well-being.
6. Providing social engagement: Social engagement is important for the emotional well-being of individuals with dementia. Encouraging social interactions, facilitating meaningful activities, and maintaining connections with family and friends can help reduce isolation and improve quality of life.
7. Supporting caregivers: Caring for someone with dementia can be physically and emotionally demanding. Supporting caregivers by providing respite care, education, emotional support, and access to community resources can help them cope with the challenges of caregiving.
8. Collaborating with healthcare professionals: Collaborating with healthcare professionals, including physicians, nurses, social workers, and other specialists, is crucial in managing the complex needs of dementia patients. Healthcare professionals can provide medical management, guidance, and support in developing care plans that are tailored to the needs of the individual with dementia.

It's important to note that dementia is a progressive condition, and the needs of individuals with dementia may change over time. Consulting with healthcare professionals and working as a team with caregivers, family members, and other stakeholders can help provide comprehensive and effective support for individuals with dementia.

Top of Form

3- How do I advocate for Alzheimer's disease?

Advocacy for Alzheimer's disease involves raising awareness, promoting understanding, and advocating for policies and resources that support individuals with Alzheimer's disease and their caregivers. Here are some ways you can advocate for Alzheimer's disease:

1. Educate yourself: Learn about Alzheimer's disease, its impact on individuals and caregivers, available resources, and the latest research and advancements. Stay informed about policies, laws, and regulations related to Alzheimer's disease at the local, national, and international levels.
2. Raise awareness: Use various channels, such as social media, public speaking, community events, and other communication platforms, to raise awareness about Alzheimer's disease. Share accurate information, dispel myths, and promote understanding and empathy towards individuals with Alzheimer's disease and their caregivers.
3. Engage with policymakers: Reach out to local, regional, national, and international policymakers to advocate for policies that support individuals with Alzheimer's disease and their caregivers. This may involve writing letters or emails, making phone calls, attending public meetings, or participating in advocacy campaigns organized by Alzheimer's advocacy organizations.
4. Join advocacy organizations: Join Alzheimer's disease advocacy organizations and participate in their advocacy efforts. These organizations often have resources, campaigns, and platforms for individuals to advocate for Alzheimer's disease collectively. They may also provide opportunities for participation in events, rallies, and awareness campaigns.
5. Share personal stories: Share your personal experiences as a caregiver, family member, or someone living with Alzheimer's disease, if comfortable and appropriate. Personal stories can be powerful advocacy tools that raise awareness, promote empathy, and inspire action.
6. Support research: Advocate for increased funding and resources for Alzheimer's disease research, including clinical trials, to advance our understanding of the disease and develop better treatments, interventions, and care strategies.
7. Collaborate with others: Partner with other individuals, organizations, and stakeholders who share similar goals in advocating for Alzheimer's disease. Collaborating with others can amplify your voice and impact.
8. Promote dementia-friendly communities: Advocate for dementia-friendly communities that promote inclusion, understanding, and support for individuals with Alzheimer's disease and their caregivers. This may involve engaging with local businesses, government agencies, and community organizations to promote dementia-friendly practices and policies.

Remember, advocating for Alzheimer's disease requires persistence, patience, and collaboration. By raising awareness, promoting understanding, engaging with policymakers, and collaborating with others, you can make a meaningful impact in advocating for individuals with Alzheimer's disease and their caregivers.

**When to transfer a with dementia to the hospital:**

- 1. When should you take someone to the hospital for dementia?

As a language model AI, I do not have the ability to provide medical advice. However, in general, there are certain situations when it may be appropriate to take someone with dementia to the hospital. These may include:

1. Acute or severe medical conditions: If a person with dementia develops a sudden or severe medical condition, such as chest pain, difficulty breathing, uncontrolled bleeding, or a significant change in their mental or physical state, it may be necessary to take them to the hospital for evaluation and treatment.
2. Injury or trauma: If a person with dementia experiences a fall, head injury, or other significant injury, it may be necessary to take them to the hospital for assessment and appropriate medical care.
3. Behavioral or psychiatric symptoms: If a person with dementia exhibits extreme or dangerous behavioral or psychiatric symptoms, such as severe agitation, aggression, suicidal or homicidal ideation, or other concerning behaviors that pose a risk to themselves or others, it may be necessary to seek immediate medical attention at the hospital.
4. Inability to manage basic activities of daily living: If a person with dementia becomes unable to manage basic activities of daily living, such as eating, drinking, grooming, or toileting, and there is a risk of dehydration, malnutrition, or other health complications, it may be necessary to seek medical attention at the hospital.
5. Caregiver inability to manage the situation: If a caregiver is unable to manage the needs or behaviors of a person with dementia at home due to their own health issues, lack of support, or other challenges, it may be necessary to seek help at the hospital for appropriate care and support.

It is important to consult with a healthcare professional, such as a primary care physician or a healthcare provider familiar with the person's medical history and condition, for appropriate guidance in determining when to take someone with dementia to the hospital. They can provide personalized advice based on the individual's specific situation and needs.

Top of Form

- 1. Why do people with dementia go to hospital?

People with dementia may go to the hospital for various reasons related to their physical health, mental health, or overall well-being. Some common reasons why people with dementia may go to the hospital include:

1. Medical conditions: People with dementia, like anyone else, can experience various medical conditions that may require hospitalization. This can include acute illnesses, chronic conditions, infections, injuries, and other physical health issues that may need medical assessment, treatment, and management.
2. Behavioral or psychiatric symptoms: Dementia can be associated with behavioral or psychiatric symptoms, such as agitation, aggression, anxiety, depression, hallucinations, delusions, and other challenging behaviors. In some cases, these symptoms may become severe or unmanageable, leading to hospitalization for assessment, stabilization, and appropriate interventions.
3. Injuries or accidents: People with dementia may be at increased risk of falls, accidents, or injuries due to cognitive impairment, mobility issues, or other factors. Hospitalization may be necessary for evaluation and treatment of injuries, fractures, or other physical trauma.
4. Changes in mental or functional status: Significant changes in mental or functional status, such as sudden decline in cognitive function, worsening of memory or communication abilities, or inability to perform basic activities of daily living, may require medical evaluation and management in a hospital setting.
5. Caregiver inability to manage at home: In some cases, caregivers of people with dementia may face challenges in managing the care needs or behaviors of their loved ones at home, due to their own health issues, lack of support, or other reasons. Hospitalization may be necessary to provide appropriate care and support for the person with dementia.
6. End-of-life care: For individuals with advanced dementia who are in the final stages of their life, hospitalization may be needed for palliative care, symptom management, and end-of-life comfort measures.

It's important to note that the decision to hospitalize a person with dementia should be based on an individualized assessment of their specific medical and care needs, taking into account their overall health status, functional abilities, behavioral symptoms, and the availability of appropriate care resources. Healthcare professionals, including primary care physicians, geriatricians, and other specialists familiar with dementia care, can provide guidance and recommendations for appropriate care and management in each unique situation.

- 1. Is it better to keep dementia patient at home?

Whether it is better to keep a person with dementia at home or in another care setting depends on various factors, including the individual's specific needs, the level of support available at home, the caregiver's abilities and resources, and the overall safety and well-being of the person with dementia. Here are some considerations:

1. Safety: Safety is a top priority for people with dementia, as they may be at risk of accidents, injuries, or other dangers due to cognitive impairments, mobility issues, and other factors. If the home environment can be adequately modified to ensure the person's safety, and if there is constant supervision and support available, keeping the person with dementia at home may be feasible.
2. Caregiver resources: Caring for a person with dementia can be physically and emotionally demanding. It requires significant time, energy, patience, and skills to provide the necessary care and support. If the caregiver has the necessary resources, including physical and mental health, social support, and access to appropriate services, keeping the person with dementia at home may be possible.
3. Quality of life: The goal of dementia care is to optimize the person's quality of life, which includes meeting their physical, emotional, social, and cognitive needs. If the home environment can provide the necessary support and stimulation to maintain the person's well-being and overall quality of life, keeping the person at home may be preferable.
4. Level of care needs: As dementia progresses, the level of care needs may increase, requiring more specialized care and support. If the person's care needs exceed the capabilities of the caregivers or the home environment, a different care setting, such as an assisted living facility or a memory care unit, may be more appropriate.
5. Caregiver well-being: Caregivers of people with dementia may experience increased stress, fatigue, and burnout, which can impact their own physical and mental health. If caregivers are struggling to provide adequate care and support at home, it may be necessary to consider other care options to ensure the well-being of both the person with dementia and the caregiver.
6. Financial considerations: The cost of dementia care, including home modifications, in-home care services, and other related expenses, can be significant. Caregivers should carefully assess their financial resources and the potential costs of keeping the person with dementia at home and weigh them against other care options.

In some cases, a combination of home-based care and outside assistance, such as in-home care services or respite care, may be a viable option for keeping a person with dementia at home. It is important to work closely with healthcare professionals, including primary care physicians, geriatricians, and other specialists, to assess the unique needs of the person with dementia and the caregiver's abilities and resources, and make informed decisions about the most appropriate care setting for the individual.

4-What are signs that dementia is getting worse?

Dementia is a progressive condition, and the symptoms tend to worsen over time. Here are some signs that may indicate dementia is getting worse:

1. Increased memory loss: One of the hallmark symptoms of dementia is memory loss, and as the condition progresses, the person may experience increased difficulty remembering recent events, names, and important information. They may repeat questions or forget familiar faces and places.
2. Decline in cognitive abilities: Dementia can affect various cognitive functions, including problem-solving, decision-making, attention, and language skills. As the condition progresses, the person may have increasing difficulty with these tasks, which can impact their ability to perform daily activities independently.
3. Changes in behavior and mood: Dementia can also cause changes in behavior and mood. The person may become more agitated, irritable, or anxious. They may exhibit mood swings, become more withdrawn or socially isolated, or engage in repetitive behaviors.
4. Decline in physical abilities: Dementia can also affect a person's physical abilities, such as coordination, balance, and mobility. As the condition progresses, the person may have difficulty with tasks such as dressing, grooming, and bathing, and may require more assistance with activities of daily living.
5. Communication difficulties: Dementia can impact a person's ability to communicate effectively. As the condition worsens, the person may have difficulty finding the right words, following conversations, or understanding others. They may also have difficulty expressing themselves or may repeat the same phrases or questions.
6. Increased dependence on others: As dementia progresses, the person may become more dependent on others for assistance with daily activities and self-care. They may require increasing levels of support and supervision to ensure their safety and well-being.
7. Disorientation and confusion: Dementia can cause disorientation and confusion, with the person becoming more confused about time, place, and people. They may have difficulty recognizing familiar surroundings, getting lost in familiar places, or becoming disoriented even in their own home.

It is important to note that the progression of dementia can vary from person to person, and not all individuals with dementia will experience the same symptoms or at the same rate. If you notice any of these signs in yourself or a loved one, it is important to consult a healthcare professional for a comprehensive evaluation and appropriate management. Early diagnosis and intervention can help with better management of dementia and support for both the person with dementia and their caregivers.

Top of Form

**Where and how to use services for patients with dementia**

- 1. What services do dementia patients need?

Dementia patients may require a range of services and support to address their physical, emotional, and cognitive needs. Some of the common services that dementia patients may need include:

1. Medical care: Dementia patients may require ongoing medical care to manage their physical health, including regular check-ups, monitoring and management of chronic health conditions, medication management, and addressing any medical issues that may arise.
2. Memory care and cognitive support: Dementia patients may benefit from memory care programs and cognitive support services that are specifically designed to help them maintain cognitive function, engage in meaningful activities, and slow the progression of cognitive decline. These may include cognitive exercises, memory-enhancing techniques, and cognitive stimulation programs.
3. Personal care: Dementia patients may require assistance with personal care activities, such as bathing, dressing, grooming, and toileting. This can help ensure their hygiene and personal well-being.
4. Caregiver support: Dementia patients often rely on caregivers for support, and caregivers may require their own support services, such as respite care, counseling, education, and support groups. Caregiver support services can help caregivers manage the challenges associated with caring for someone with dementia and maintain their own physical and emotional well-being.
5. Home modifications: Modifying the home environment to make it safe and supportive for someone with dementia may be necessary. This may include installing safety features like grab bars, removing tripping hazards, improving lighting, and making other adjustments to minimize risks and enhance safety.
6. Social engagement and activities: Dementia patients may benefit from social engagement and meaningful activities that can help improve their quality of life and provide mental stimulation. This may include social activities, hobbies, exercise programs, music therapy, and other activities that promote cognitive, emotional, and physical well-being.
7. Behavioral and emotional support: Dementia patients may experience behavioral and emotional symptoms, such as agitation, anxiety, depression, and mood swings. Behavioral and emotional support services, including counseling, therapy, and medication management, may be needed to address these symptoms and help the person manage their emotional well-being.
8. End-of-life care: In advanced stages of dementia, end-of-life care and palliative care may be needed to provide comfort and support for the person with dementia and their family. This may include pain management, emotional support, and assistance with advanced care planning.

The specific services needed for dementia patients may vary depending on the stage of dementia, the individual's needs and preferences, and the available resources in the local community. It is important to work with a healthcare professional or a care team to determine and access the appropriate services and support for the unique needs of each person with dementia.

- 1. What are dementia care services?

Dementia care services are specialized services designed to support individuals with dementia and their families in managing the physical, emotional, and cognitive challenges associated with dementia. These services are typically provided by trained professionals and may be available in various settings, including home care, assisted living facilities, memory care units, and skilled nursing facilities. Some common dementia care services include:

1. Personal care: Assistance with activities of daily living (ADLs) such as bathing, dressing, grooming, toileting, and mobility support to help individuals with dementia maintain their personal hygiene, physical well-being, and independence.
2. Memory care and cognitive support: Memory care programs and cognitive support services that provide specialized care and activities designed to support cognitive function, engage individuals with dementia in meaningful activities, and slow the progression of cognitive decline. These may include cognitive exercises, memory-enhancing techniques, and cognitive stimulation programs.
3. Medication management: Assistance with medication management, including proper administration of prescribed medications, monitoring for side effects or interactions, and coordination with healthcare providers to ensure that medications are being taken as prescribed.
4. Behavioral and emotional support: Assistance with managing and addressing behavioral and emotional symptoms associated with dementia, such as agitation, anxiety, depression, mood swings, and challenging behaviors. This may include counseling, therapy, and medication management to help manage these symptoms and improve emotional well-being.
5. Social engagement and activities: Social engagement programs and meaningful activities designed to promote social interaction, cognitive stimulation, and emotional well-being for individuals with dementia. This may include social activities, hobbies, exercise programs, music therapy, and other activities that promote engagement and quality of life.
6. Caregiver support: Support services for family caregivers of individuals with dementia, including respite care, education, counseling, and support groups. Caregiver support services can help caregivers manage the challenges associated with caring for someone with dementia and maintain their own physical and emotional well-being.
7. Home modifications: Modifying the home environment to make it safe and supportive for someone with dementia, including installing safety features like grab bars, removing tripping hazards, improving lighting, and making other adjustments to minimize risks and enhance safety.
8. End-of-life care: In advanced stages of dementia, end-of-life care and palliative care may be needed to provide comfort and support for the person with dementia and their family. This may include pain management, emotional support, and assistance with advanced care planning.

The specific dementia care services provided may vary depending on the stage of dementia, the individual's needs and preferences, and the available resources in the local community. It is important to work with a healthcare professional or a care team to determine and access the appropriate dementia care services for the unique needs of each person with dementia.

Top of Form

- 1. Where are most patients with dementia cared for?

The care setting for individuals with dementia can vary depending on the severity of the condition, the availability of care options, and the preferences of the person with dementia and their family. Some common care settings for individuals with dementia include:

1. Home care: Many individuals with dementia are cared for at home, with family members or other caregivers providing care in the familiar surroundings of the person's own home. Home care may involve informal caregiving by family members, friends, or other informal caregivers, as well as formal home care services provided by professional caregivers or home health agencies.
2. Assisted living facilities: Assisted living facilities are residential care settings that provide support and services for individuals who need assistance with activities of daily living (ADLs), including those with dementia. These facilities typically offer private or shared rooms, meals, assistance with personal care, medication management, and social engagement activities.
3. Memory care units: Memory care units are specialized units within assisted living facilities or nursing homes that are designed specifically for individuals with dementia. These units provide specialized care, programming, and a secure environment to meet the unique needs of individuals with dementia, including those with behaviors that may be challenging or require specialized care.
4. Skilled nursing facilities: Skilled nursing facilities, also known as nursing homes or long-term care facilities, provide round-the-clock medical care and support for individuals with complex medical needs, including individuals with advanced dementia who require higher levels of care, supervision, and medical management.
5. Hospice care: Hospice care is specialized care provided to individuals who are in the final stages of a terminal illness, including individuals with dementia. Hospice care focuses on providing comfort, pain management, and emotional support to individuals with advanced dementia and their families in their own home, a hospice facility, or another care setting.

The care setting for individuals with dementia may vary depending on factors such as the stage of dementia, the level of care needed, the availability of resources, and the preferences and circumstances of the person with dementia and their family. It is important to work with healthcare professionals and care teams to determine the most appropriate care setting for the individual with dementia based on their unique needs and circumstances.

- 1. What is the best way to help someone with dementia?

The best way to help someone with dementia depends on their individual needs, preferences, and circumstances. However, here are some general tips that may be helpful:

1. Establish and maintain a routine: Individuals with dementia often benefit from a predictable routine, as it can help reduce confusion and agitation. Establish a consistent schedule for daily activities such as meals, medication, bathing, and bedtime.
2. Use clear and simple language: When communicating with someone with dementia, use clear and simple language, speak slowly and calmly, and use non-verbal cues such as gestures and facial expressions to help convey your message. Avoid using complex or abstract language that may be confusing.
3. Create a safe environment: Make the environment safe and supportive for the person with dementia. Remove tripping hazards, install handrails, and ensure that the home or care setting is well-lit. Consider using locks or alarms to prevent wandering or other safety concerns.
4. Provide reassurance and validation: Individuals with dementia may experience confusion, anxiety, or frustration. Provide reassurance and validation, and avoid correcting or arguing with them. Instead, redirect their attention or offer comfort and support.
5. Foster social engagement: Encourage social engagement and meaningful activities, such as hobbies, music, art, or reminiscing. Engage the person in activities that they enjoy and that align with their interests and abilities.
6. Seek support and education: Caring for someone with dementia can be challenging, and it's important to seek support and education to better understand the condition and learn coping strategies. This may include joining support groups, attending caregiver education programs, or seeking guidance from healthcare professionals.
7. Practice self-care: Caring for someone with dementia can be emotionally and physically demanding. It's important to prioritize your own physical and mental well-being. Make sure to take breaks, get adequate rest, eat well, and seek support for yourself as needed.
8. Involve healthcare professionals: Work closely with healthcare professionals, including doctors, nurses, and other members of the healthcare team, to develop a care plan tailored to the individual's needs. They can provide guidance, support, and appropriate medical interventions to help manage the symptoms of dementia.

Remember that dementia is a progressive condition, and the needs of individuals with dementia may change over time. It's important to regularly reassess and adjust the care plan based on the person's evolving needs and preferences. Always consult with healthcare professionals for personalized guidance and care recommendations for someone with dementia.

Top of Form

**Financial help and services for patients with dementia**

- 1. How can I help a dementia patient with money?

Assisting a person with dementia with managing their money can be complex, as their cognitive abilities and decision-making skills may be impaired. Here are some general tips for helping a dementia patient with money:

1. Legal and financial planning: If possible, work with the person with dementia and their family or legal representative to establish legal and financial plans in advance. This may include creating a durable power of attorney for finances, establishing a trust, or setting up automatic bill payments.
2. Simplify financial tasks: Simplify financial tasks for the person with dementia. For example, consolidate their accounts, limit the number of credit cards, and streamline their financial paperwork. Minimize the complexity and the number of financial decisions they need to make.
3. Monitor and safeguard finances: Regularly monitor the person's financial accounts for any signs of irregularities, such as unusual transactions or bills. Safeguard their finances by securing important documents, such as social security cards, bank statements, and financial records in a safe place.
4. Limit access to money: Consider limiting the person's access to cash or credit cards to prevent overspending or financial exploitation. Use caution in providing access to financial resources, and consider involving a trusted family member or caregiver in managing their finances.
5. Seek professional financial advice: Consider seeking professional financial advice from a qualified financial planner or elder law attorney who has experience in handling financial matters related to dementia. They can provide guidance on financial management strategies, estate planning, and other legal and financial matters.
6. Communicate with compassion: When discussing financial matters with a person with dementia, use clear and simple language, be patient, and communicate with compassion. Avoid arguing or criticizing their financial decisions, and instead focus on finding practical solutions that prioritize their best interests.
7. Report suspected financial abuse: If you suspect that a person with dementia is being financially exploited or abused, report it to the appropriate authorities, such as law enforcement, adult protective services, or the person's healthcare provider. Taking action to protect their finances is important to safeguard their well-being.

It's essential to approach financial matters with sensitivity and respect for the person's autonomy and dignity. Consulting with legal and financial professionals who have experience in dealing with dementia-related issues can provide valuable guidance and support in managing financial affairs for someone with dementia.

Top of Form

- 1. What services can help people with dementia?

There are various services that can help people with dementia, depending on their specific needs and stage of the disease. Some of the commonly available services for individuals with dementia include:

1. Memory care programs: Memory care programs are specialized care programs designed specifically for individuals with dementia or Alzheimer's disease. These programs typically provide a structured and supportive environment, with trained staff who understand the unique needs of individuals with dementia.
2. Home care services: Home care services provide assistance and support to individuals with dementia in their own homes. This can include help with personal care, meal preparation, medication management, and companionship. Home care services can be tailored to the individual's needs and can help them maintain their independence and quality of life in familiar surroundings.
3. Respite care: Respite care offers temporary relief to caregivers of individuals with dementia. Respite care can be provided at home or in a care facility and allows caregivers to take a break from their caregiving responsibilities, while ensuring that the person with dementia receives appropriate care and supervision.
4. Adult day programs: Adult day programs are daytime programs that provide structured activities, socialization, and supervision for individuals with dementia. These programs can offer respite for caregivers while providing meaningful activities and social engagement for individuals with dementia.
5. Memory clinics: Memory clinics are specialized healthcare settings that provide comprehensive evaluations, diagnosis, and ongoing management for individuals with memory disorders, including dementia. Memory clinics typically have a multidisciplinary team of healthcare professionals who can provide specialized care, support, and education for individuals with dementia and their families.
6. Support groups: Support groups are available for both individuals with dementia and their caregivers. These groups offer emotional support, education, and information sharing in a supportive environment. Support groups can be in-person or online and provide an opportunity for individuals with dementia and their caregivers to connect with others who are going through similar experiences.
7. Counseling and therapy: Counseling and therapy services, including individual counseling, family therapy, and behavioral therapy, can help individuals with dementia and their families cope with the emotional and psychological challenges associated with the disease. Counseling and therapy can provide support for managing stress, depression, anxiety, and other emotional issues.
8. Home modifications and assistive devices: Modifying the home environment and using assistive devices can help individuals with dementia maintain their safety and independence. Examples of home modifications include installing grab bars, improving lighting, and removing tripping hazards. Assistive devices such as memory aids, reminder systems, and wearable tracking devices can also be helpful in managing the challenges associated with dementia.
9. Case management and care coordination: Case management and care coordination services can help individuals with dementia and their families navigate the complex healthcare and social service systems, ensure access to appropriate services and resources, and coordinate care among different providers.

These are just a few examples of the services that can help individuals with dementia. The specific services needed will depend on the individual's needs, stage of dementia, and available resources in their community. Consulting with healthcare professionals, social workers, or geriatric care managers can be helpful in identifying and accessing appropriate services for individuals with dementia.

- 1. What is the financial impact of dementia?

The financial impact of dementia can be significant, both for individuals with dementia and their families. Dementia is a progressive brain disorder that can cause cognitive decline, memory loss, and functional impairment, which can result in increased care needs and associated costs. Some of the financial impacts of dementia may include:

1. Healthcare costs: Individuals with dementia may require ongoing medical care, including regular check-ups, diagnostic tests, medications, and specialized care for managing the symptoms of dementia. As the disease progresses, the need for medical care and interventions may increase, resulting in higher healthcare costs.
2. Long-term care costs: Many individuals with dementia require long-term care as the disease progresses. This may include in-home care services, assisted living facilities, memory care units, or nursing homes. Long-term care can be expensive, and the costs can vary depending on the level of care needed, location, and duration of care.
3. Caregiver costs: Family members or other caregivers who provide care for individuals with dementia may also experience financial impacts. Caregivers may need to reduce their work hours or quit their jobs to provide care, resulting in lost income and potential impacts on retirement savings. Caregivers may also incur expenses related to caregiving, such as transportation, home modifications, and assistive devices.
4. Legal and financial planning costs: Planning for the financial and legal aspects of dementia, such as creating a power of attorney, setting up a trust, or managing financial affairs, may also involve costs, including legal and financial consultation fees.
5. Lost income and productivity: Individuals with dementia who are still in the workforce may experience reduced productivity or may need to take time off work due to cognitive decline or other symptoms of the disease. This can result in lost income and potential impacts on career advancement or retirement savings.
6. Financial exploitation: Individuals with dementia may be vulnerable to financial exploitation, such as scams, fraud, or undue influence by others. This can result in financial losses and impacts on the individual's financial well-being.
7. Economic burden on society: Dementia poses a significant economic burden on society as a whole, including healthcare costs, long-term care costs, and productivity losses for caregivers and individuals with dementia who are still in the workforce. This can have broader societal impacts, including increased healthcare spending, strain on social services, and potential impacts on the economy.

It's important to note that the financial impact of dementia can vary widely depending on the severity of the disease, available support systems, geographic location, and other factors. It's recommended to seek financial and legal advice early in the dementia journey to plan for the potential financial impacts and explore available resources and support options.

Top of Form

**Legal issues for patients with dementia**

- 1. What are the 4 issues people with dementia have?

As individuals with dementia experience cognitive decline and functional impairment, they may face several common issues. Four of the most prominent issues faced by people with dementia include:

1. Memory loss: Memory loss is a hallmark symptom of dementia, particularly in the early stages. People with dementia may have difficulty remembering recent events, familiar faces, names, and other information. This can impact their ability to communicate, perform daily tasks, and maintain independence.
2. Communication difficulties: Dementia can impair language skills and communication abilities. People with dementia may struggle to find the right words, express themselves clearly, or understand others. This can lead to frustration, social isolation, and difficulties in expressing their needs and preferences.
3. Behavioral changes: Dementia can cause changes in behavior, mood, and personality. People with dementia may exhibit agitation, aggression, wandering, depression, anxiety, or other challenging behaviors. These behavioral changes can be distressing for both the person with dementia and their caregivers, and may require appropriate management strategies and interventions.
4. Functional impairment: Dementia can gradually impair an individual's ability to perform daily tasks such as dressing, bathing, eating, managing finances, and other activities of daily living (ADLs). This can result in increased dependence on others for care, reduced quality of life, and potential safety risks.

It's important to note that the specific issues experienced by individuals with dementia can vary depending on the type and stage of dementia, as well as individual variations. Some individuals may have additional symptoms or challenges, while others may experience these issues to a lesser extent. Proper diagnosis, individualized care plans, and support from healthcare professionals, caregivers, and other resources can help address these issues and improve the quality of life for individuals with dementia.

- 1. What is an ethical issue with dementia patients?

There are several ethical issues that can arise in the care of individuals with dementia. One common ethical issue is the balance between respecting autonomy and ensuring their safety and well-being. For example, individuals with dementia may exhibit behaviors that are risky or harmful to themselves or others, such as wandering or refusing necessary medical treatment. This can raise ethical questions about the appropriate level of intervention and restriction of autonomy to ensure the person's safety and well-being while still respecting their autonomy and preserving their dignity.

Another ethical issue is the issue of informed consent. As dementia progresses, individuals may have difficulty understanding and making informed decisions about their own care. This can pose challenges in obtaining valid consent for medical treatments, participation in research, or other interventions. Healthcare providers and caregivers may need to make decisions on behalf of individuals with dementia, following ethical principles such as beneficence (acting in the person's best interest), respect for autonomy (respecting their values and preferences to the extent possible), and promoting their well-being.

The issue of end-of-life care is another ethical concern in dementia care. Decisions regarding advance directives, palliative care, and end-of-life wishes may need to be made in the context of the person's dementia and their ability to express their preferences. Ethical considerations may arise in determining the most appropriate care goals and interventions, as well as addressing potential conflicts among family members or caregivers regarding end-of-life decisions.

Additionally, issues related to privacy, confidentiality, and the sharing of personal information may arise in dementia care. As individuals with dementia may have limited capacity to understand the implications of sharing personal information, caregivers and healthcare providers must carefully navigate issues of privacy and confidentiality while still providing appropriate care and support.

It's important to recognize that ethical issues in dementia care can be complex and multifaceted, and may require careful consideration of the unique circumstances and needs of each individual. Healthcare providers, caregivers, and family members involved in the care of individuals with dementia should approach these issues with compassion, respect, and a commitment to upholding ethical principles while promoting the well-being of the person with dementia. Consulting with healthcare professionals or ethicists experienced in dementia care can be helpful in navigating these complex ethical issues.

3-What are the practical issues affecting patients with dementia?

There are several practical issues that can affect patients with dementia. These may include:

1. Daily living activities: Dementia can impact a person's ability to perform activities of daily living (ADLs) independently, such as bathing, dressing, grooming, toileting, eating, and mobility. As the disease progresses, patients may require increasing assistance and support with these basic activities, which can impact their overall quality of life and increase the burden on caregivers.
2. Medication management: Many individuals with dementia may require medications to manage their symptoms or treat other health conditions. However, dementia can affect a person's ability to remember to take medications as prescribed, understand dosage instructions, and manage complex medication regimens. This can lead to issues with medication adherence and potentially adverse health outcomes.
3. Safety concerns: Dementia can impair a person's judgment, decision-making, and awareness of their environment, which can increase their risk for accidents, falls, wandering, and other safety concerns. Caregivers and family members may need to implement safety measures in the home or other settings to protect the person with dementia from harm.
4. Communication difficulties: Dementia can affect a person's ability to communicate effectively. Patients may have difficulty finding the right words, following conversations, understanding others, or expressing their own thoughts and feelings. This can impact their ability to express their needs and preferences, understand instructions, and engage in social interactions.
5. Legal and financial issues: Dementia can impact a person's ability to make sound legal and financial decisions. Patients may be vulnerable to financial exploitation, scams, or other legal and financial issues. Caregivers and family members may need to take steps to protect the person's legal and financial interests, such as establishing power of attorney or guardianship.
6. Caregiver support: Dementia can place a significant burden on caregivers who may need to provide ongoing care and support to the person with dementia. Caregivers may experience physical, emotional, and financial strain, and may need support in managing their caregiving responsibilities while also taking care of their own well-being.
7. Access to appropriate care services: Patients with dementia may require access to various care services, such as medical care, memory care, home care, respite care, or specialized dementia care programs. Access to appropriate care services may depend on factors such as location, availability, affordability, and the specific needs and preferences of the person with dementia.

These are some of the practical issues that can affect patients with dementia. It's important to work with healthcare professionals, caregivers, and other support systems to address these practical issues and ensure that individuals with dementia receive the appropriate care and support to maximize their well-being and quality of life.

- 1. What are the rights of a dementia patient?

Dementia patients, like all individuals, have rights that should be respected and upheld. Some of the key rights of a dementia patient include:

1. Right to dignity and respect: Dementia patients have the right to be treated with dignity and respect at all times, regardless of their cognitive abilities. This includes being addressed by their preferred name, being included in decision-making processes to the extent possible, and being treated in a compassionate and person-centered manner.
2. Right to autonomy: Dementia patients have the right to make their own choices and decisions to the extent possible, based on their cognitive abilities and stage of dementia. This includes choices related to their daily activities, living arrangements, healthcare decisions, and participation in social, cultural, and recreational activities.
3. Right to safety and protection: Dementia patients have the right to be protected from abuse, neglect, exploitation, and harm. This includes ensuring that they are in a safe and secure environment, and that appropriate measures are taken to prevent accidents, falls, wandering, and other safety risks.
4. Right to receive appropriate medical and nursing care: Dementia patients have the right to receive appropriate medical and nursing care to address their physical, emotional, and mental health needs. This includes access to timely and accurate diagnosis, appropriate treatment and management of dementia symptoms, and palliative care in the advanced stages of the disease.
5. Right to privacy and confidentiality: Dementia patients have the right to privacy and confidentiality, including the protection of their personal and medical information. This includes obtaining informed consent for any medical or research interventions, and ensuring that their personal information is not shared without their consent, unless required by law.
6. Right to advocacy and representation: Dementia patients have the right to have an advocate or representative who can help ensure that their rights and interests are protected. This may include a family member, caregiver, or other appointed representative who can speak on their behalf, provide support, and advocate for their needs and preferences.
7. Right to cultural and spiritual expression: Dementia patients have the right to express their cultural and spiritual beliefs and preferences, and to have access to appropriate cultural and spiritual practices and support, based on their background, beliefs, and preferences.
8. Right to end-of-life care: Dementia patients have the right to receive appropriate end-of-life care that respects their dignity, preferences, and wishes. This includes the right to receive palliative care, to have a care plan that aligns with their values and goals, and to be surrounded by loved ones during their final moments.

It's important to recognize and respect the rights of dementia patients, and to work collaboratively with healthcare professionals, caregivers, and other stakeholders to ensure that their rights are upheld throughout the course of their disease. Additionally, it's crucial to be aware of local laws and regulations that may govern the rights and protections of dementia patients in your specific region or country.

**Insurance issues for patients with dementia**

- 1. Does dementia affect life insurance?

Yes, dementia can affect life insurance in several ways. When an individual with dementia applies for life insurance, or if a person who already has a life insurance policy is diagnosed with dementia, it can impact the coverage, premiums, and policy terms. Some potential effects of dementia on life insurance include:

1. Higher premiums: Life insurance premiums are typically based on the individual's age, health, and other risk factors. Dementia is considered a serious health condition, and insurers may view it as a higher risk, resulting in higher premiums for individuals with dementia or a history of dementia in their family.
2. Limited coverage options: Individuals with dementia may face limited options for life insurance coverage. Some insurance companies may decline to provide coverage to individuals with dementia, while others may offer coverage with certain limitations, such as lower coverage amounts or shorter policy terms.
3. Medical underwriting and disclosure requirements: Dementia may require additional medical underwriting and disclosure requirements during the life insurance application process. This may include providing detailed medical history, undergoing medical examinations or tests, and disclosing any previous diagnosis or treatment for dementia.
4. Exclusions and limitations: Life insurance policies may have exclusions or limitations related to dementia or other pre-existing conditions. These exclusions or limitations may limit or exclude coverage for certain types of death, such as death due to dementia-related causes.
5. Timing and stage of dementia: The timing and stage of dementia at the time of life insurance application can also impact the coverage and premiums. Early-stage dementia may have less impact on coverage and premiums compared to advanced-stage dementia.

It's important to note that life insurance companies have their own underwriting guidelines and may treat dementia differently based on their policies and practices. It's essential to thoroughly review the terms and conditions of any life insurance policy, and if you have questions or concerns related to dementia and life insurance, it's best to consult with a qualified insurance professional or financial advisor who can provide guidance based on your specific circumstances.

- 1. How does dementia affect travel insurance?

Dementia can impact travel insurance coverage in several ways. Travel insurance is designed to provide coverage for unexpected events, including medical emergencies, during travel. However, dementia, as a pre-existing condition, may affect the coverage and terms of travel insurance policies. Here are some potential ways how dementia can affect travel insurance:

1. Medical coverage: Individuals with dementia may require additional medical coverage due to the risk of medical emergencies related to their condition. Travel insurance policies may have limitations or exclusions related to pre-existing medical conditions, including dementia. This means that any medical expenses related to dementia, such as hospitalization, medical consultations, or medication, may not be covered by the travel insurance policy.
2. Travel cancellation or interruption: Dementia may also impact travel cancellation or interruption coverage. If a person with dementia is unable to travel or has to interrupt their trip due to a worsening of their condition, travel insurance may provide coverage for reimbursement of non-refundable trip expenses or additional travel expenses. However, policy terms and conditions may vary, and coverage for dementia-related cancellations or interruptions may be limited or excluded.
3. Travel assistance: Travel insurance may also provide travel assistance services, such as emergency medical evacuation, repatriation of remains, or medical escorts. However, coverage for these services may be impacted by the presence of dementia as a pre-existing condition.
4. Disclosure requirements: Similar to life insurance, travel insurance policies may require individuals with dementia to disclose their medical condition during the application process. Failure to disclose the pre-existing condition of dementia may result in denial of coverage or cancellation of the policy.
5. Policy premiums: Travel insurance premiums may also be affected by dementia. Individuals with dementia or a history of dementia in their family may be considered higher risk by insurers, resulting in higher premiums or even denial of coverage.

It's important to review the terms and conditions of any travel insurance policy thoroughly, and if you have questions or concerns related to dementia and travel insurance, it's best to consult with a qualified insurance professional or travel insurance provider who can provide guidance based on your specific circumstances. Additionally, it's important to be transparent and disclose any pre-existing medical conditions, including dementia, during the travel insurance application process to ensure that you have appropriate coverage and avoid potential issues with claim reimbursement in the future.

- 1. What are the actual problems for dementia patients?

Dementia patients face a wide range of challenges and problems that can significantly impact their daily lives. These problems may include:

1. Cognitive decline: Dementia is characterized by cognitive decline, including memory loss, difficulties with language, problem-solving, decision-making, and changes in judgment. These cognitive deficits can make it challenging for individuals with dementia to perform everyday tasks and engage in social activities.
2. Communication difficulties: As dementia progresses, communication can become increasingly difficult. Patients may struggle to express themselves or understand others, leading to frustration, confusion, and isolation.
3. Behavioral and psychological symptoms: Dementia patients may exhibit a range of behavioral and psychological symptoms, such as agitation, aggression, anxiety, depression, hallucinations, delusions, and sleep disturbances. These symptoms can be distressing for both the patient and their caregivers and may require careful management and intervention.
4. Activities of daily living (ADL) impairment: Dementia can impair the ability to perform basic activities of daily living (ADLs), such as dressing, bathing, grooming, eating, and using the toilet. This can result in increased dependence on caregivers for assistance with these activities.
5. Safety risks: Dementia patients may face safety risks due to their cognitive decline and behavioral symptoms. They may wander, become disoriented, have difficulties with mobility, forget to take medications or engage in risky behaviors, which can pose risks to their health and safety.
6. Caregiver burden: Dementia care often falls on family members and caregivers who may experience significant physical, emotional, and financial burdens. Caregivers may face challenges in managing the care needs of a loved one with dementia, balancing work and family responsibilities, coping with the emotional toll of the disease, and navigating the healthcare system.
7. Social isolation: Dementia can lead to social isolation as patients may have difficulties with communication, engagement in social activities, and may withdraw from social interactions due to embarrassment or fear of stigma. This can result in reduced social connections, loneliness, and a diminished quality of life.
8. Financial and legal challenges: Dementia patients may face financial and legal challenges, such as managing finances, making legal decisions, and protecting their rights. This may require assistance from caregivers or legal professionals to ensure their financial and legal affairs are properly managed.
9. Care coordination: Coordinating care for dementia patients can be complex, involving multiple healthcare providers, specialists, and caregivers. Managing appointments, medications, and coordinating care can be challenging and may require additional support and resources.
10. End-of-life care planning: Dementia is a progressive and terminal disease, and end-of-life care planning becomes a significant consideration for patients and their families. This may involve discussions about advance directives, palliative care, and other end-of-life care decisions.

It's important to recognize that dementia is a complex and multifaceted condition that affects individuals in different ways. Each person with dementia may have unique challenges and needs, and it's crucial to provide person-centered care that takes into consideration their individual circumstances and provides appropriate support and interventions to address their specific problems.

- 1. Can I claim benefits if I have dementia?

Yes, individuals with dementia may be eligible for certain benefits, depending on their country of residence and the specific program requirements. Benefits can provide financial assistance to help cover the costs associated with dementia care, support services, and living expenses. Here are some potential benefits that individuals with dementia may be eligible for:

1. Social Security Disability Benefits: In the United States, individuals with dementia who are no longer able to work due to their condition may be eligible for Social Security Disability Insurance (SSDI) or Supplemental Security Income (SSI) benefits, depending on their work history and income level.
2. Pension and Retirement Benefits: Individuals who have contributed to a pension or retirement plan during their working years may be eligible for pension or retirement benefits, depending on the terms and conditions of their plan.
3. Veterans Benefits: Veterans who have served in the military and have been diagnosed with dementia may be eligible for benefits through the Department of Veterans Affairs (VA), including disability compensation, pensions, and healthcare services.
4. Long-Term Care Insurance: If an individual with dementia has a long-term care insurance policy, they may be able to make a claim to cover the costs of dementia care services, such as in-home care, assisted living, or nursing home care, depending on the terms and coverage of their policy.
5. Medicaid: Medicaid is a state and federal program in the United States that provides healthcare coverage for low-income individuals, including those with dementia. Medicaid may cover medical services, long-term care services, and other supports for individuals with dementia who meet the program's eligibility requirements.
6. Carer's Allowance or Caregiver Benefits: In some countries, caregivers who provide care for individuals with dementia may be eligible for caregiver benefits or carer's allowance, which provides financial assistance to help offset the costs of caregiving, such as lost wages or additional expenses related to providing care.

It's important to note that eligibility for benefits can vary depending on the country, state, or province, and specific program requirements. It's recommended to consult with relevant government agencies, healthcare providers, or legal professionals to understand the benefits that may be available for individuals with dementia in your specific location and circumstances.

**Home Help for patients with dementia**

- 1. What dementia home care services are available?

There are a variety of dementia home care services available to provide support and assistance for individuals with dementia in the comfort of their own home. These services can be tailored to meet the unique needs and challenges associated with dementia, and may include:

1. Personal Care Assistance: This may include help with activities of daily living (ADLs) such as bathing, dressing, grooming, toileting, and mobility assistance.
2. Medication Management: Assistance with medication reminders, organizing medications, and ensuring medications are taken as prescribed.
3. Home Health Care: Skilled nursing services provided by registered nurses (RNs) or licensed practical nurses (LPNs), including wound care, medication administration, and monitoring of vital signs.
4. Memory Care Programs: Specialized programs designed specifically for individuals with dementia, which may include cognitive stimulation activities, reminiscence therapy, and social engagement to help maintain cognitive function and quality of life.
5. Respite Care: Temporary relief for primary caregivers, providing them with a break from caregiving responsibilities while ensuring that the person with dementia is well cared for at home.
6. Companionship: Socialization, companionship, and engagement in activities to reduce social isolation and improve the emotional well-being of the individual with dementia.
7. Home Safety Assessments: Evaluation of the home environment to identify potential safety hazards for individuals with dementia, and recommendations for modifications to promote safety and reduce the risk of falls or accidents.
8. Meal Preparation: Assistance with meal planning and preparation, taking into consideration any dietary restrictions or preferences of the individual with dementia.
9. Transportation Assistance: Help with transportation to medical appointments, social outings, or other activities as needed.
10. Home Modifications: Recommendations for home modifications, such as installing grab bars, removing trip hazards, and improving lighting, to make the home environment safer and more conducive to the needs of the individual with dementia.

It's important to note that the availability and scope of dementia home care services may vary depending on the location and resources available in your community. It's recommended to consult with a healthcare professional, geriatric care manager, or a reputable home care agency to discuss the specific needs and preferences of the individual with dementia and to determine the most appropriate home care services for their situation.

Top of Form

- 1. How do I prepare to give dementia care at home?

Preparing to provide dementia care at home requires careful planning and consideration of the unique needs and challenges associated with dementia. Here are some steps to help you prepare:

1. Educate Yourself: Learn as much as you can about dementia, including its stages, symptoms, and common behavioral changes. Understanding the disease can help you anticipate and respond effectively to the needs and behaviors of the person with dementia.
2. Create a Safe Environment: Make the home environment safe and conducive to the needs of the person with dementia. This may include removing tripping hazards, installing grab bars, improving lighting, and securing dangerous items such as medications, cleaning supplies, and sharp objects.
3. Seek Support: Caring for someone with dementia can be physically and emotionally challenging. Seek support from family, friends, or a support group to share the caregiving responsibilities, provide emotional support, and help you manage stress.
4. Develop a Care Plan: Create a care plan that outlines the daily routine, activities, and tasks for the person with dementia. This may include a schedule for medication administration, meal planning, and personal care routines.
5. Communicate Clearly: Communication can be difficult with dementia patients due to language and cognitive changes. Use simple and clear language, maintain eye contact, and use visual aids and gestures to help convey your message. Be patient and avoid arguing or correcting the person with dementia, as this can lead to frustration and agitation.
6. Practice Compassionate Care: Dementia patients may exhibit challenging behaviors such as agitation, aggression, or confusion. Practice compassionate care by using empathy, validation, and redirection techniques to address their needs and emotions. Avoid using force or restraint unless it is necessary for safety reasons.
7. Plan for Respite Care: Caring for someone with dementia can be physically and emotionally exhausting. Plan for respite care options to give yourself regular breaks to rest and rejuvenate.
8. Get Professional Help: Consider engaging the services of a home care agency or a professional caregiver with experience in dementia care. They can provide specialized care, support, and expertise to help you manage the challenges associated with dementia.
9. Take Care of Yourself: Self-care is important as a caregiver. Make sure to prioritize your physical and emotional well-being by getting enough rest, eating healthily, and seeking emotional support when needed.
10. Be Flexible: Dementia is a progressive disease, and the needs of the person with dementia may change over time. Be prepared to adapt and adjust your care plan as needed to accommodate the changing needs of the individual with dementia.

Remember that caring for someone with dementia at home can be demanding, and it's important to seek help, support, and resources to ensure the well-being of both the person with dementia and yourself as the caregiver. Consult with healthcare professionals, geriatric care managers, or other reputable sources for guidance and assistance in providing dementia care at home.

**Transportation options for patients with dementia**

1. Can you travel abroad if you have dementia?

Whether or not someone with dementia can travel abroad depends on the stage and severity of their condition, as well as other individual factors. Here are some considerations to keep in mind:

1. Safety: The safety of the person with dementia should be the top priority. Consider the person's ability to navigate unfamiliar environments, follow instructions, manage self-care, and respond to emergency situations. Dementia patients may have impaired judgment, memory loss, and difficulty with orientation, which can pose risks while traveling.
2. Medical Care: Consider the availability and accessibility of appropriate medical care at the travel destination. Dementia patients may require ongoing medical management, including medication administration, monitoring of health conditions, and access to healthcare professionals. Research the availability of local medical facilities and ensure that necessary medical information and documentation, including medications and medical records, are available during the trip.
3. Travel Logistics: Traveling can be physically and emotionally taxing, and the person with dementia may have difficulties with mobility, fatigue, or increased confusion. Consider the duration and mode of transportation of the trip, including long flights, layovers, and changes in time zones, which may disrupt sleep patterns and exacerbate symptoms of dementia.
4. Caregiver Support: If you are a caregiver traveling with someone with dementia, consider your ability to provide appropriate care and support during the trip. This includes managing medications, helping with personal care, managing behaviors, and addressing any emergencies or challenging situations that may arise.
5. Legal and Financial Considerations: Ensure that you have the necessary legal and financial documents in place, such as power of attorney, advance directives, and travel insurance. These documents can help you make decisions on behalf of the person with dementia and manage any legal or financial matters that may arise during the trip.
6. Travel Insurance: Consider obtaining travel insurance that specifically covers individuals with pre-existing medical conditions, including dementia. Review the coverage details carefully, including medical coverage, emergency medical evacuation, trip cancellation or interruption, and other relevant benefits.
7. Destination Considerations: Research the destination thoroughly, including the local customs, culture, language, healthcare system, and accessibility. Consider the availability of familiar foods, routines, and environments that may help reduce confusion and agitation for the person with dementia.

It's important to consult with the person's healthcare provider, as well as other relevant professionals, such as a geriatric care manager or travel medicine specialist, before making any travel decisions for someone with dementia. They can provide personalized guidance based on the individual's condition and help you make an informed decision about whether traveling abroad is safe and feasible.

1. What facility is best for dementia patients?

The best facility for dementia patients depends on several factors, including the stage and severity of dementia, the individual's unique needs and preferences, and the available resources and support systems. Here are some options to consider:

1. Home-based care: Many dementia patients prefer to stay in their own homes for as long as possible, especially during the early stages of dementia. Home-based care can be provided by family caregivers or professional caregivers who come to the person's home to provide assistance with daily activities, medication management, personal care, and companionship. Home-based care can provide familiar surroundings, routines, and minimal disruption to the person's lifestyle.
2. Memory care facilities: Memory care facilities, also known as dementia care or Alzheimer's care facilities, are specialized residential care facilities that cater specifically to individuals with dementia. These facilities are designed to provide a safe and supportive environment for individuals with dementia, with trained staff who are experienced in caring for dementia patients. Memory care facilities typically offer specialized programs, activities, and environments to meet the unique needs of individuals with dementia, including secure environments to prevent wandering, cognitive stimulation programs, and tailored care plans.
3. Assisted living communities: Assisted living communities provide housing, meals, and assistance with activities of daily living (ADLs) for seniors, including those with dementia. These communities typically offer a range of services and amenities, such as 24-hour staff availability, social activities, and medical oversight. Some assisted living communities may also have designated memory care units or provide specialized dementia care services.
4. Skilled nursing facilities: Skilled nursing facilities, also known as nursing homes or long-term care facilities, provide 24-hour nursing care and medical services for individuals with complex medical needs, including those with advanced dementia. Skilled nursing facilities may have specialized dementia care units or provide dementia care services within their facility.
5. Adult day care programs: Adult day care programs provide supervised care and activities during daytime hours for individuals with dementia, allowing family caregivers to continue working or take a break from caregiving responsibilities. Adult day care programs typically provide socialization, cognitive stimulation, and assistance with ADLs, and may also offer therapeutic activities and support services.

The best facility for a dementia patient depends on their individual needs, preferences, and the available resources. It's important to carefully evaluate the options, visit different facilities, and consult with healthcare professionals, including the person's healthcare provider and a geriatric care manager, to determine the most appropriate and suitable care setting for the individual with dementia.

1. Can people with dementia travel by plane?

The ability of people with dementia to travel by plane depends on several factors, including the stage and severity of dementia, the individual's overall health and functional abilities, and the level of support available during the travel process. Here are some considerations:

1. Early stages of dementia: In the early stages of dementia, some individuals may still be able to travel by plane with appropriate support and planning. They may be able to follow instructions, manage their personal care, and communicate their needs. However, it's important to carefully assess the individual's cognitive and functional abilities, as well as their overall health, to determine if air travel is safe and appropriate for them.
2. Mid to late stages of dementia: As dementia progresses, individuals may experience challenges with memory, orientation, communication, and functional abilities, which can make air travel more difficult and risky. They may become agitated, confused, or disoriented in unfamiliar environments, and may have difficulty following instructions or managing their personal care. Air travel can also be physically demanding and exhausting, which may be particularly challenging for individuals with advanced dementia.
3. Safety considerations: Air travel involves navigating through airports, security screenings, boarding, and disembarking from the aircraft, which can be overwhelming for individuals with dementia. There is also a risk of wandering or getting lost, especially during layovers or transfers. Additionally, the sensory overload of air travel, including noise, bright lights, and unfamiliar surroundings, can be distressing for individuals with dementia and may exacerbate their symptoms.
4. Support and preparation: If air travel is being considered for a person with dementia, it's important to ensure that there is adequate support and preparation in place. This may include having a travel companion who is familiar with the person's needs and can provide assistance, contacting the airline in advance to request accommodations such as priority boarding or special seating, carrying all necessary documents and identification, and having a contingency plan in case of emergencies or unexpected situations.
5. Consultation with healthcare professionals: It's crucial to consult with the person's healthcare provider, including their primary care physician and neurologist or geriatrician, to assess the individual's overall health and cognitive abilities, and to seek their professional opinion on the appropriateness and safety of air travel for the person with dementia.

In summary, whether a person with dementia can travel by plane depends on their individual abilities, overall health, level of support, and the stage of dementia. It's important to carefully evaluate the risks and benefits, and to seek guidance from healthcare professionals to make an informed decision.

The ability of people with dementia to travel by plane depends on several factors, including the stage and severity of dementia, the individual's overall health and functional abilities, and the level of support available during the travel process. Here are some considerations:

1. Early stages of dementia: In the early stages of dementia, some individuals may still be able to travel by plane with appropriate support and planning. They may be able to follow instructions, manage their personal care, and communicate their needs. However, it's important to carefully assess the individual's cognitive and functional abilities, as well as their overall health, to determine if air travel is safe and appropriate for them.
2. Mid to late stages of dementia: As dementia progresses, individuals may experience challenges with memory, orientation, communication, and functional abilities, which can make air travel more difficult and risky. They may become agitated, confused, or disoriented in unfamiliar environments, and may have difficulty following instructions or managing their personal care. Air travel can also be physically demanding and exhausting, which may be particularly challenging for individuals with advanced dementia.
3. Safety considerations: Air travel involves navigating through airports, security screenings, boarding, and disembarking from the aircraft, which can be overwhelming for individuals with dementia. There is also a risk of wandering or getting lost, especially during layovers or transfers. Additionally, the sensory overload of air travel, including noise, bright lights, and unfamiliar surroundings, can be distressing for individuals with dementia and may exacerbate their symptoms.
4. Support and preparation: If air travel is being considered for a person with dementia, it's important to ensure that there is adequate support and preparation in place. This may include having a travel companion who is familiar with the person's needs and can provide assistance, contacting the airline in advance to request accommodations such as priority boarding or special seating, carrying all necessary documents and identification, and having a contingency plan in case of emergencies or unexpected situations.
5. Consultation with healthcare professionals: It's crucial to consult with the person's healthcare provider, including their primary care physician and neurologist or geriatrician, to assess the individual's overall health and cognitive abilities, and to seek their professional opinion on the appropriateness and safety of air travel for the person with dementia.

In summary, whether a person with dementia can travel by plane depends on their individual abilities, overall health, level of support, and the stage of dementia. It's important to carefully evaluate the risks and benefits, and to seek guidance from healthcare professionals to make an informed decision.

4- What services do dementia patients need?

Dementia patients require a range of services to support their physical, cognitive, emotional, and social well-being. Here are some key services that can benefit individuals with dementia:

1. Medical care: Dementia patients need access to appropriate medical care, including regular check-ups, medication management, and treatment of other health conditions. This may involve working closely with their primary care physician, neurologist, geriatrician, or other healthcare providers to ensure that their medical needs are met.
2. Cognitive stimulation: Engaging in activities that provide cognitive stimulation can help maintain cognitive function and slow the progression of dementia. This may include activities such as puzzles, games, reading, or other mentally stimulating activities tailored to the individual's interests and abilities.
3. Assistance with activities of daily living (ADLs): Dementia patients may require assistance with activities of daily living (ADLs) such as bathing, dressing, grooming, toileting, and eating. Home health aides, personal care assistants, or other trained caregivers can provide support with these activities to help maintain the person's dignity, safety, and well-being.
4. Memory care programs: Memory care programs or memory care units within senior living communities or specialized care facilities are designed to provide specialized care and support for individuals with dementia. These programs may offer structured activities, cognitive stimulation, social engagement, and specialized care approaches tailored to the unique needs of individuals with dementia.
5. Behavioral and emotional support: Dementia patients may experience changes in behavior, mood, and emotions, such as agitation, depression, anxiety, and aggression. Services that provide behavioral and emotional support, such as counseling, therapy, or support groups, can help manage these symptoms and improve the person's overall well-being.
6. Respite care: Caregiving for a person with dementia can be physically and emotionally demanding. Respite care services, such as in-home respite care or short-term stays in specialized care facilities, can provide temporary relief for caregivers, allowing them to take breaks, rest, and attend to their own well-being.
7. Care coordination and case management: Dementia patients may benefit from care coordination and case management services, which help ensure that their care is coordinated among different healthcare providers, services, and settings. This can help optimize care, manage transitions, and address any challenges or issues that may arise.
8. Home modifications and safety assessments: Modifying the home environment to be safe and supportive for individuals with dementia is important. Services such as home assessments, home modifications, and safety evaluations can help identify and address potential hazards or challenges in the home, such as fall risks, wandering risks, or other safety concerns.
9. Legal and financial planning: Dementia patients and their families may benefit from legal and financial planning services, including estate planning, power of attorney, guardianship, and financial management. These services can help ensure that the person's legal and financial affairs are in order and that their best interests are protected.
10. Social engagement and community support: Social engagement and community support are important for the emotional well-being of dementia patients. Services such as adult day programs, social clubs, support groups, and other community-based activities can provide opportunities for social interaction, meaningful engagement, and emotional support.

The specific services needed for dementia patients may vary depending on the individual's stage of dementia, overall health, and unique needs. It's important to work closely with healthcare professionals, caregivers, and support organizations to develop a care plan that addresses the person's specific needs and enhances their quality of life.

**Stress Management for patients with dementia**

- 1. What are the methods of stress management in the elderly?

Stress management is important for the elderly population, including those who may be experiencing cognitive decline or have dementia. Here are some methods of stress management that can be beneficial for the elderly:

1. Relaxation techniques: Various relaxation techniques such as deep breathing, progressive muscle relaxation, meditation, and mindfulness can help reduce stress and promote relaxation. These techniques can be practiced regularly to help manage stress and anxiety.
2. Exercise: Regular physical activity can help reduce stress, anxiety, and depression. Elderly individuals can engage in gentle forms of exercise such as walking, tai chi, yoga, or water aerobics, based on their abilities and health conditions. Exercise not only helps with stress management but also promotes overall physical and mental well-being.
3. Social support: Maintaining social connections and having a support system can help reduce stress in the elderly. Spending time with family, friends, and participating in social activities can provide emotional support and companionship, which can help alleviate stress.
4. Cognitive stimulation: Engaging in activities that provide cognitive stimulation, such as reading, solving puzzles, playing games, or learning new skills, can help keep the mind active and reduce stress. Cognitive stimulation activities can help maintain cognitive function and improve overall well-being in the elderly.
5. Time management: Effective time management can help reduce stress by prioritizing tasks and activities, setting realistic goals, and avoiding unnecessary stressors. Caregivers can help the elderly with time management, ensuring that they have a balanced schedule and can avoid feeling overwhelmed.
6. Adequate sleep: Getting sufficient sleep is important for stress management. Elderly individuals should aim for good sleep hygiene, including establishing a regular sleep schedule, creating a comfortable sleep environment, and practicing relaxation techniques before bedtime.
7. Nutritional health: Eating a balanced diet that is rich in nutrients can help support overall health and well-being, which in turn can reduce stress. It's important for the elderly to eat a nutritious diet and stay hydrated to support their physical and mental health.
8. Supportive environment: Creating a supportive and safe environment can help reduce stress in the elderly. This can involve making necessary home modifications, having a safe and comfortable living space, and ensuring that the elderly person has access to necessary resources, support services, and medical care.
9. Counseling or therapy: Professional counseling or therapy can be beneficial for elderly individuals who are experiencing stress or mental health concerns. Licensed counselors, psychologists, or geriatric mental health specialists can provide support, guidance, and tools to manage stress and cope with challenging emotions.
10. Medication management: For elderly individuals who are on medications, proper medication management can be essential to prevent medication-related stress. Organizing medications, adhering to prescribed dosages, and seeking help from healthcare professionals when needed can help prevent stress related to medication management.

It's important to work with healthcare professionals, caregivers, and support networks to develop a comprehensive stress management plan that is tailored to the unique needs and abilities of the elderly individual. It's also crucial to monitor for signs of stress or mental health concerns in the elderly and seek appropriate professional help when needed.

- 1. How to respond when a person with dementia is expressing stress and distress?

When a person with dementia is expressing stress and distress, it's important to respond with empathy and understanding. Here are some general guidelines for responding to a person with dementia who is experiencing stress or distress:

1. Stay calm: It's important to remain calm and composed when responding to a person with dementia who is expressing stress or distress. Avoid reacting with frustration or impatience, as this can escalate the situation.
2. Validate feelings: Acknowledge and validate the person's feelings. You can say things like "I can see that you're upset" or "It sounds like you're feeling worried." Validating their emotions can help them feel heard and understood.
3. Use simple and clear communication: Use simple, clear, and concise language when communicating with a person with dementia. Avoid using complex or lengthy sentences, and use a calm and reassuring tone of voice. Speak slowly and allow the person enough time to process the information.
4. Provide reassurance: Offer reassurance to the person with dementia. You can say things like "You're safe" or "I'm here to help." Reassurance can help calm their anxiety and reduce their distress.
5. Redirect or distract: If the person with dementia is fixated on a particular topic or is becoming increasingly agitated, you can try redirecting their attention to something else or distracting them with a different activity or topic. For example, you can suggest going for a walk, listening to music, or engaging in a favorite hobby.
6. Avoid arguing or correcting: It's important to avoid arguing or correcting a person with dementia who is expressing stress or distress. This can often escalate the situation and cause further distress. Instead, try to empathize with their feelings and offer reassurance.
7. Provide comfort and physical touch: Physical touch, such as holding their hand or giving a gentle hug, can be comforting for a person with dementia who is feeling stressed or distressed. Comforting gestures and physical touch can help establish a sense of connection and reassurance.
8. Use familiar and calming cues: Using familiar and calming cues, such as playing familiar music, showing familiar photos, or using familiar scents, can help soothe a person with dementia who is experiencing stress or distress. Familiar cues can evoke positive memories and associations, which can help reduce distress.
9. Seek professional help if needed: If the person with dementia is experiencing persistent stress or distress, it's important to involve healthcare professionals, such as a doctor, nurse, or geriatric care specialist, to assess the situation and provide appropriate interventions or treatments.

Remember, each person with dementia is unique, and what may work for one person may not work for another. It's important to approach each situation with patience, empathy, and flexibility, and to work closely with healthcare professionals and caregivers to provide the best possible care for the person with dementia.

- 1. What activities reduce anxiety for dementia patients?

Engaging in appropriate activities can help reduce anxiety for dementia patients. Here are some examples of activities that may help:

1. Music therapy: Listening to familiar and calming music can have a positive effect on dementia patients. Music has been shown to evoke positive emotions, stimulate memories, and promote relaxation, which can help reduce anxiety.
2. Art and crafts: Engaging in simple art and craft activities, such as coloring, painting, or making simple crafts, can be enjoyable for dementia patients and provide a sense of accomplishment. These activities can also help improve hand-eye coordination and stimulate creativity.
3. Sensory stimulation: Sensory stimulation activities, such as touching different textures, smelling pleasant scents, or listening to calming sounds, can help distract and relax dementia patients. Sensory activities can also help promote relaxation and reduce anxiety.
4. Physical activity: Gentle physical activities, such as walking, chair exercises, or tai chi, can help reduce anxiety in dementia patients. Physical activity can improve mood, increase relaxation, and promote better sleep, which can all help reduce anxiety.
5. Memory and cognitive exercises: Engaging in memory and cognitive exercises, such as puzzles, word games, or reminiscence activities, can help stimulate the brain and promote cognitive function in dementia patients. These activities can also provide a sense of accomplishment and boost self-esteem.
6. Familiar hobbies: Encouraging dementia patients to engage in familiar hobbies or activities that they enjoyed in the past can help reduce anxiety. This can include activities such as gardening, cooking, knitting, or playing a musical instrument. Familiar hobbies can provide a sense of comfort and familiarity, and help reduce anxiety and stress.
7. Socialization: Maintaining social connections and engaging in social activities can help reduce anxiety in dementia patients. This can include spending time with loved ones, participating in group activities, or attending social gatherings specifically designed for individuals with dementia. Socialization can help reduce feelings of isolation and promote a sense of belonging.

It's important to assess the abilities and interests of each individual with dementia and tailor the activities to their preferences and capabilities. It's also important to provide a safe and supportive environment for engaging in activities, and to monitor for any signs of distress or agitation during the activities. Consulting with healthcare professionals, such as a doctor, nurse, or occupational therapist, can also provide guidance on appropriate activities for dementia patients and help address any specific needs or concerns.

Top of Form

- 1. How do you manage stress in patients?

Managing stress in patients can be approached in a holistic manner that takes into account their physical, emotional, and mental well-being. Here are some general strategies that can be helpful in managing stress in patients:

1. Establish a calm and supportive environment: Creating a calm and supportive environment can help reduce stress in patients. This can include minimizing loud noises, providing comfortable and familiar surroundings, and ensuring a predictable routine. Creating a safe and calming environment can help patients feel more relaxed and at ease.
2. Effective communication: Clear and compassionate communication is crucial in managing stress in patients. Listening to their concerns, validating their emotions, and providing reassurance can help patients feel heard and understood. Using simple and clear language, visual aids, and other communication techniques tailored to the patient's abilities can also be helpful.
3. Emotional support: Providing emotional support is essential in managing stress in patients. This can include offering comfort, empathy, and validation of their emotions. Encouraging patients to express their feelings and providing appropriate outlets for emotional expression, such as talking, crying, or engaging in creative activities, can also be beneficial.
4. Relaxation techniques: Teaching and practicing relaxation techniques with patients can help them manage stress. This can include techniques such as deep breathing, progressive muscle relaxation, guided imagery, and mindfulness. These techniques can help patients relax, reduce tension, and manage anxiety.
5. Medication and medical interventions: In some cases, medication and medical interventions may be necessary to manage stress in patients. This may include medications to manage physical symptoms that contribute to stress, such as pain or discomfort, or medications to address mental health conditions, such as anxiety or depression. It's important to consult with healthcare professionals, such as doctors or nurses, for appropriate and safe use of medications and medical interventions.
6. Engaging in enjoyable activities: Encouraging patients to engage in enjoyable activities can help reduce stress. This can include activities such as listening to music, engaging in hobbies, spending time with loved ones, or participating in activities that bring them joy. Engaging in pleasurable activities can help distract from stressors and promote a sense of well-being.
7. Social support: Social support can be invaluable in managing stress in patients. Encouraging patients to maintain social connections, spend time with loved ones, and participate in group activities can help reduce stress and promote a sense of belonging and support.
8. Collaborating with a healthcare team: Collaborating with a healthcare team, which may include doctors, nurses, social workers, and other healthcare professionals, can help manage stress in patients. They can provide tailored interventions and support based on the patient's specific needs and circumstances.

It's important to consider the unique needs and preferences of each patient and to approach stress management in a person-centered and holistic manner. Consulting with healthcare professionals and incorporating a multi-disciplinary approach can provide the most effective strategies for managing stress in patients.

**Managing emotions for patients with dementia**

1. How do you help someone with dementia emotionally?

Supporting someone with dementia emotionally can be challenging, but it's crucial for their well-being. Here are some ways you can help someone with dementia emotionally:

1. Validate their emotions: Acknowledge and validate the person's emotions, even if their thoughts or feelings may not be based on reality. Avoid arguing or correcting their perceptions, as this can cause frustration and agitation. Instead, offer empathy and reassurance, and try to understand and validate their emotions from their perspective.
2. Provide comfort and reassurance: Offer comfort and reassurance to help soothe their emotional distress. Use gentle touch, calming words, and a calm tone of voice to provide comfort. Reassure the person that they are safe and cared for, and offer words of comfort and support.
3. Engage in calming activities: Engage the person with dementia in calming activities that they enjoy. This can include listening to music, looking at old photos, engaging in sensory activities such as stroking soft fabrics, or engaging in familiar hobbies or activities that they enjoy. These activities can help distract from distressing emotions and promote a sense of calm.
4. Maintain routines and familiar environments: Keeping routines and environments familiar can help reduce emotional distress for someone with dementia. Changes in routine or environment can be confusing and distressing for them. Keeping their living environment familiar and maintaining regular routines can help create a sense of stability and reduce emotional distress.
5. Use positive communication techniques: Use positive communication techniques when interacting with the person with dementia. This includes speaking in a calm and reassuring manner, using simple and clear language, avoiding complex questions, and giving them ample time to process and respond. Positive and respectful communication can help reduce confusion, frustration, and emotional distress.
6. Encourage social interactions: Social interactions and connections are important for emotional well-being. Encourage social interactions with family members, friends, and other supportive individuals, as appropriate. Social engagement, even if it's just through simple conversations or spending time together, can help reduce feelings of isolation and loneliness.
7. Provide caregiver support: Caring for someone with dementia can be emotionally taxing for caregivers as well. It's important for caregivers to prioritize their own emotional well-being and seek support when needed. This can include talking to a trusted friend or family member, joining a caregiver support group, or seeking professional counseling or therapy.
8. Consult with healthcare professionals: Consulting with healthcare professionals, such as doctors, nurses, or social workers, can provide valuable guidance and support for emotional care for someone with dementia. They can provide recommendations, interventions, and strategies tailored to the individual's unique needs and circumstances.

It's important to approach emotional care for someone with dementia with patience, empathy, and understanding. Each person with dementia is unique, and their emotional needs may change over time. It's crucial to adapt and modify your approach based on their individual needs and preferences, and to seek professional help when needed.

1. What are 3 typical emotional responses of a person with dementia?

Three typical emotional responses of a person with dementia may include:

1. Agitation: Agitation is a common emotional response in people with dementia. It can manifest as restlessness, irritability, pacing, or increased anxiety. Agitation may be triggered by various factors such as environmental changes, unfamiliar situations, unmet needs, or physical discomfort.
2. Confusion: Confusion is another common emotional response in people with dementia. Due to the cognitive impairment associated with dementia, individuals may become confused about their surroundings, time, or people. This confusion can lead to frustration, fear, and anxiety.
3. Emotional distress: People with dementia may also experience emotional distress, which can manifest as sadness, fear, anger, or mood swings. Emotional distress may arise from a sense of loss, changes in routine or environment, difficulty in understanding or communicating, or other challenging situations.

It's important to note that emotional responses in people with dementia can vary widely depending on the individual, the stage of dementia, and other factors. Each person with dementia is unique, and their emotional responses may change over time. It's important to approach these emotional responses with empathy, patience, and understanding, and to tailor care and support based on the individual's needs and preferences. Consulting with healthcare professionals, such as doctors, nurses, or social workers, can provide valuable guidance and support in managing emotional responses in people with dementia.

1. What are the coping strategies for dementia patients?

Coping strategies for dementia patients can vary depending on the stage of dementia and the individual's unique needs and preferences. Here are some general coping strategies that may be helpful for dementia patients:

1. Establishing a routine: Maintaining a consistent routine can provide a sense of structure and familiarity, which can help reduce confusion and anxiety in dementia patients. This can include regular meal times, medication schedules, and daily activities.
2. Simplifying the environment: Simplifying the physical environment can help dementia patients navigate their surroundings more easily. This can involve decluttering, reducing noise and distractions, and using clear signage or labels to identify rooms or objects.
3. Providing reassurance and validation: Dementia patients may experience confusion or distress due to memory loss or difficulty understanding their surroundings. Providing reassurance and validation of their feelings and experiences can help alleviate anxiety and build trust.
4. Encouraging engagement in meaningful activities: Engaging in meaningful activities, such as hobbies, crafts, or familiar tasks, can help dementia patients maintain a sense of purpose, accomplishment, and enjoyment. Activities should be tailored to the individual's abilities and interests.
5. Using memory aids: Memory aids, such as calendars, clocks, notes, or reminders, can help dementia patients keep track of time, appointments, and important information.
6. Practicing effective communication: Communication can become challenging for dementia patients as the condition progresses. Using simple and clear language, speaking slowly, and using nonverbal cues, such as facial expressions and gestures, can improve communication with dementia patients.
7. Seeking support: Caregivers and family members of dementia patients may also need to cope with the challenges of caregiving. Seeking support from healthcare professionals, support groups, or counselors can provide emotional support, education, and practical advice.

It's important to work with healthcare professionals, such as doctors, nurses, or occupational therapists, to develop and implement coping strategies that are tailored to the individual needs of the dementia patient. Each person with dementia is unique, and their coping strategies may need to be adjusted over time as the condition progresses.

Top of Form

1. Why is emotional support important for dementia patients?

Emotional support is crucial for dementia patients for several reasons:

1. Promotes emotional well-being: Dementia patients may experience a range of emotions, including confusion, frustration, fear, anxiety, and sadness, due to the cognitive and functional changes associated with the condition. Emotional support can help validate their feelings and emotions, reduce distress, and promote emotional well-being.
2. Enhances quality of life: Emotional support can improve the overall quality of life for dementia patients by helping them feel more secure, comfortable, and understood. It can also foster positive relationships, promote engagement in meaningful activities, and improve their sense of self-worth and dignity.
3. Reduces behavioral symptoms: Dementia patients may exhibit challenging behaviors, such as agitation, aggression, wandering, or withdrawal, which can be distressing for both the individual and their caregivers. Emotional support can help address the underlying emotional needs or triggers that may contribute to such behaviors, leading to better management and reduction of these symptoms.
4. Supports cognitive function: Emotional distress, stress, and anxiety can negatively impact cognitive function in dementia patients. Providing emotional support can help reduce these negative effects and support better cognitive function, including memory, attention, and decision-making.
5. Enhances caregiver well-being: Caregivers of dementia patients also require emotional support to cope with the demands and challenges of caregiving. Providing emotional support to dementia patients can also alleviate caregiver stress, anxiety, and burnout, enabling them to provide better care and support to their loved ones.
6. Promotes positive relationships: Emotional support can foster positive relationships between dementia patients and their caregivers, family members, and friends. It can strengthen the bond between individuals, create a sense of trust, and improve communication and understanding, leading to improved interactions and overall well-being.

In summary, emotional support is essential for dementia patients as it promotes emotional well-being, enhances quality of life, reduces behavioral symptoms, supports cognitive function, and fosters positive relationships. It plays a vital role in helping dementia patients cope with the challenges of their condition and live a better, more fulfilling life.

Top of Form

**Reference**

1. Armstrong, M. J., N. Gamez, S. Alliance, T. Majid, A. S. Taylor, A. M. Kurasz, B. Patel and G. E. Smith (2021). "Clinical care and unmet needs of individuals with dementia with Lewy bodies and caregivers: an interview study." Alzheimer Disease & Associated Disorders **35**(4): 327-334.
2. Boughtwood, D., C. Shanley, J. Adams, Y. Santalucia, H. Kyriazopoulos, D. Pond and J. Rowland (2012). "Dementia information for culturally and linguistically diverse communities: sources, access and considerations for effective practice." Australian Journal of Primary Health **18**(3): 190-196.
3. de Jong, J. D. and F. Boersma (2009). "Dutch psychogeriatric day-care centers: a qualitative study of the needs and wishes of carers." International psychogeriatrics **21**(2): 268-277.
4. Ducharme, F., M.-J. Kergoat, R. Coulombe, L. Lévesque, P. Antoine and F. Pasquier (2014). "Unmet support needs of early-onset dementia family caregivers: a mixed-design study." BMC nursing **13**: 1-10.
5. Edelman, P., D. Kuhn, B. R. Fulton and G. A. Kyrouac (2006). "Information and service needs of persons with Alzheimer's disease and their family caregivers living in rural communities." American Journal of Alzheimer's Disease & Other Dementias® **21**(4): 226-233.
6. Farran, C. J., D. Loukissa, S. Perraud and O. Paun (2003). "Alzheimer's disease caregiving information and skills. Part I: Care recipient issues and concerns." Research in nursing & health **26**(5): 366-375.
7. Forbes, D. A., S. Finkelstein, C. M. Blake, M. Gibson, D. G. Morgan, M. Markle-Reid, I. Culum and E. Thiessen (2012). "Knowledge exchange throughout the dementia care journey by Canadian rural community-based health care practitioners, persons with dementia, and their care partners: an interpretive descriptive study." Rural and Remote Health **12**(4): 1-15.
8. Galvin, J. E., J. E. Duda, D. I. Kaufer, C. F. Lippa, A. Taylor and S. H. Zarit (2010). "Lewy body dementia: caregiver burden and unmet needs." Alzheimer Disease & Associated Disorders **24**(2): 177-181.
9. Georges, J., S. Jansen, J. Jackson, A. Meyrieux, A. Sadowska and M. Selmes (2008). "Alzheimer's disease in real life–the dementia carer's survey." International journal of geriatric psychiatry **23**(5): 546-551.
10. Hirakawa, Y., M. Kuzuya, H. Enoki and K. Uemura (2011). "Information needs and sources of family caregivers of home elderly patients." Archives of gerontology and geriatrics **52**(2): 202-205.
11. Huis in het Veld, J. G., R. Verkaik, B. van Meijel, P.-J. Verkade, W. Werkman, C. M. Hertogh and A. L. Francke (2018). "Self-management support and ehealth when managing changes in behavior and mood of a relative with dementia: an asynchronous online focus group study of family caregivers' needs." Research in gerontological nursing **11**(3): 151-159.
12. Iribarren, S., S. Stonbraker, N. Suero-Tejeda, M. Granja, J. A. Luchsinger, M. Mittelman, S. Bakken and R. Lucero (2019). "Information, communication, and online tool needs of Hispanic family caregivers of individuals with Alzheimer's disease and related dementias." Inform Health Soc Care **44**(2): 115-134.
13. Jensen, C. J. and J. Inker (2015). "Strengthening the dementia care triad: identifying knowledge gaps and linking to resources." American Journal of Alzheimer's Disease & Other Dementias® **30**(3): 268-275.
14. Killen, A., D. Flynn, A. De Brún, N. O’Brien, J. O’Brien, A. J. Thomas, I. McKeith and J.-P. Taylor (2016). "Support and information needs following a diagnosis of dementia with Lewy bodies." International Psychogeriatrics **28**(3): 495-501.
15. Koenig, K. N., V. Steiner and L. L. Pierce (2011). "Information needs of family caregivers of persons with cognitive versus physical deficits." Gerontology & Geriatrics Education **32**(4): 396-413.
16. Rosa, E., G. Lussignoli, F. Sabbatini, A. Chiappa, S. Di Cesare, L. Lamanna and O. Zanetti (2010). "Needs of caregivers of the patients with dementia." Archives of gerontology and geriatrics **51**(1): 54-58.
17. Scharett, E., K. C. Madathil, S. Lopes, H. Rogers, S. Agnisarman, S. Narasimha, A. Ashok and C. Dye (2017). "An investigation of the information sought by caregivers of Alzheimer's patients on online peer support groups." Cyberpsychology, behavior, and social networking **20**(10): 640-657.
18. Shreve, J., R. Baier, G. Epstein-Lubow and R. Gardner (2016). "Dementia caregivers’ technology preferences: Design insights from qualitative interviews." Gerontechnology **14**(2): 89-96.
19. Thomas, P., S. Chantoin‐Merlet, C. Hazif‐Thomas, J. Belmin, B. Montagne, J. P. Clément, M. Lebruchec and R. Billon (2002). "Complaints of informal caregivers providing home care for dementia patients: the Pixel study." International journal of geriatric psychiatry **17**(11): 1034-1047.
20. Turner, S. and H. Street (1999). "Assessing carers' training needs: a pilot inquiry." Aging & Mental Health **3**(2): 173-178.
21. Vaingankar, J. A., M. Subramaniam, L. Picco, G. K. Eng, S. Shafie, R. Sambasivam, Y. J. Zhang, V. Sagayadevan and S. A. Chong (2013). "Perceived unmet needs of informal caregivers of people with dementia in Singapore." International Psychogeriatrics **25**(10): 1605-1619.
22. Wackerbarth, S. B. and M. M. Johnson (2002). "Essential information and support needs of family caregivers." Patient education and counseling **47**(2): 95-100.
