# Supplementary material for: Evaluation of ChatGPT’s responses to information needs and information seeking of dementia patients
Source: Sci Rep. 2024 May 4;14:10273. doi: 10.1038/s41598-024-61068-5 (PMC11069588; doi:10.1038/s41598-024-61068-5)
Supplement: Supplementary file 3 — Supplementary Information 3. [file 41598_2024_61068_MOESM3_ESM.docx]

**Supplementary file 3**

Questionnaire using a 5-point Likert scale (5 = “I agree very much” and 1 = “I don't agree at all”)

|  | ChatGPT was very good at answering my informational needs (see items below). Really met my information needs. | 1 = I don't agree at all | 2= I don't agree | 3= I partially agree | 4= I agree | 5 = I agree very much |
| --- | --- | --- | --- | --- | --- | --- |
| 1 | Treatment |  |  |  |  |  |
| 2 | Prognosis |  |  |  |  |  |
| 3 | Current Medication |  |  |  |  |  |
| 4 | Current Research |  |  |  |  |  |
| 5 | Genetic Aspects |  |  |  |  |  |
| 6 | Hygiene |  |  |  |  |  |
| 7 | Controlling Emotions |  |  |  |  |  |
| 8 | Stress Controlling |  |  |  |  |  |
| 9 | Modes of Transportation |  |  |  |  |  |
| 10 | Home Help |  |  |  |  |  |
| 11 | Insurance Issues |  |  |  |  |  |
| 12 | Legal Issues |  |  |  |  |  |
| 13 | Financial Contribution and Services |  |  |  |  |  |
| 14 | Use Services |  |  |  |  |  |
| 15 | Move to the Hospital |  |  |  |  |  |
| 16 | Advocate for Patients with Dementia |  |  |  |  |  |
| 17 | How to deal with the Family and Friends of Patients |  |  |  |  |  |
| 18 | Negative Effects on Family and Community |  |  |  |  |  |
| 19 | Information appropriate to different levels |  |  |  |  |  |
| 20 | Chance of Recovery |  |  |  |  |  |
| 21 | Memory Skills Patients with Dementia |  |  |  |  |  |
| 22 | Food and Nutritional Information |  |  |  |  |  |
| 23 | Behavior and Safety issues |  |  |  |  |  |
| 24 | Coping with hallucinations |  |  |  |  |  |
| 25 | Communication difficulties and how to manage |  |  |  |  |  |
| 26 | Daily Activities for Patients with Dementia |  |  |  |  |  |
| 27 | First aid for Patients with Dementia |  |  |  |  |  |
| 28 | Emergency situations |  |  |  |  |  |
| 29 | Conflict resolution for Patients with Dementia |  |  |  |  |  |
| 30 | Patient Ethics |  |  |  |  |  |
| 31 | Helpful Experiences of Other Caregivers |  |  |  |  |  |
